# Proton Radiation Hardness of Perovskite Tandem Photovoltaics

**DOI:** 10.1016/j.joule.2020.03.006

**Published:** 2020-05-20

**Authors:** Felix Lang, Marko Jošt, Kyle Frohna, Eike Köhnen, Amran Al-Ashouri, Alan R. Bowman, Tobias Bertram, Anna Belen Morales-Vilches, Dibyashree Koushik, Elizabeth M. Tennyson, Krzysztof Galkowski, Giovanni Landi, Mariadriana Creatore, Bernd Stannowski, Christian A. Kaufmann, Jürgen Bundesmann, Jörg Rappich, Bernd Rech, Andrea Denker, Steve Albrecht, Heinz-Christoph Neitzert, Norbert H. Nickel, Samuel D. Stranks

**Affiliations:** 1 Cavendish Laboratory, Department of Physics, University of Cambridge, Cambridge CB30HE, UK; 2 Young Investigator Group Perovskite Tandem Solar Cells, Helmholtz-Zentrum Berlin für Materialien und Energie GmbH, Berlin 12489, Germany; 3 PVcomB, Helmholtz-Zentrum Berlin für Materialien und Energie GmbH, Berlin 12489, Germany; 4 Plasma and Materials Processing, Department of Applied Physics, Eindhoven University of Technology, (TU/e), Eindhoven, 5600 MB, the Netherlands; 5 Institute of Physics, Faculty of Physics, Astronomy and Informatics, Nicolaus Copernicus University, Toruń, 87-100, Poland; 6 Department of Industrial Engineering, (DIIn), Salerno University, Fisciano, SA 84084, Italy; 7 Helmholtz-Zentrum Berlin für Materialien und Energie GmbH, und Energie GmbH, Protonen für die Therapie, Berlin 14109, Germany; 8 Institute for Silicon Photovoltaics, Helmholtz-Zentrum Berlin für Materialien und Energie GmbH, Berlin 12489, Germany; 9 Technical University Berlin, Faculty IV, Electrical Engineering and Computer Science, Berlin, Germany; 10 Beuth Hochschule für Technik Berlin, Fachbereich II – Mathematik – Physik – Chemie, Luxemburgerstr. 10, Berlin 13353, Germany; 11 Department of Chemical Engineering & Biotechnology, University of Cambridge, Cambridge CB3 0AS, UK

**Keywords:** perovskite, tandem solar cell, multijunction solar cell, radiation hardness, space photovoltaics, radiation-induced defects, degradation, perovskite/CIGS, perovskite/silicon, perovsktite tandem

## Abstract

Monolithic [Cs_0.05_(MA_0.__17_FA_0.__83_)_0.95_]Pb(I_0.83_Br_0.17_)_3_/Cu(In,Ga)Se_2_ (perovskite/CIGS) tandem solar cells promise high performance and can be processed on flexible substrates, enabling cost-efficient and ultra-lightweight space photovoltaics with power-to-weight and power-to-cost ratios surpassing those of state-of-the-art III-V semiconductor-based multijunctions. However, to become a viable space technology, the full tandem stack must withstand the harsh radiation environments in space. Here, we design tailored *operando* and *ex situ* measurements to show that perovskite/CIGS cells retain over 85% of their initial efficiency even after 68 MeV proton irradiation at a dose of 2 × 10^12^ p^+^/cm^2^. We use photoluminescence microscopy to show that the local quasi-Fermi-level splitting of the perovskite top cell is unaffected. We identify that the efficiency losses arise primarily from increased recombination in the CIGS bottom cell and the nickel-oxide-based recombination contact. These results are corroborated by measurements of monolithic perovskite/silicon-heterojunction cells, which severely degrade to 1% of their initial efficiency due to radiation-induced recombination centers in silicon.

## Highlights

• Halide perovskite sub-cells exhibit strong proton irradiation resiliency
• Novel *operando* characterization distinguishes degradation of individual sub-cells
• Perovskite/CIGS tandem solar cells retain 85% of their initial efficiency after irradiation
• Perovskite/SHJ tandem solar cells degrade to 1% of their initial efficiency after irradiation

## Context & Scale

Monolithic perovskite/silicon and perovskite/CIGS tandem solar cells could facilitate large-scale decarbonization of the power sector, provided their long-term stability is proven. In this work, we test the stability of both technologies under high-energy proton irradiation. While this mimics the radiation environment in space, our versatile *operando* and *ex situ* methodology is also suitable for studying the long-term stability of multijunction solar cells for terrestrial applications. We find that perovskite/silicon tandem solar cells are unsuitable for space, whereas perovskite/CIGS tandems are radiation-hard, promising cheap, flexible, and ultra-lightweight space photovoltaics. Both the growing demand for smaller, cheaper satellites and the privatization of space exploration are revolutionizing space economics, providing an ideal niche for the commercialization of this new technology until the levelized cost-of-electricity can compete with current terrestrial photovoltaics.

## Graphical Abstract

**Figure:**
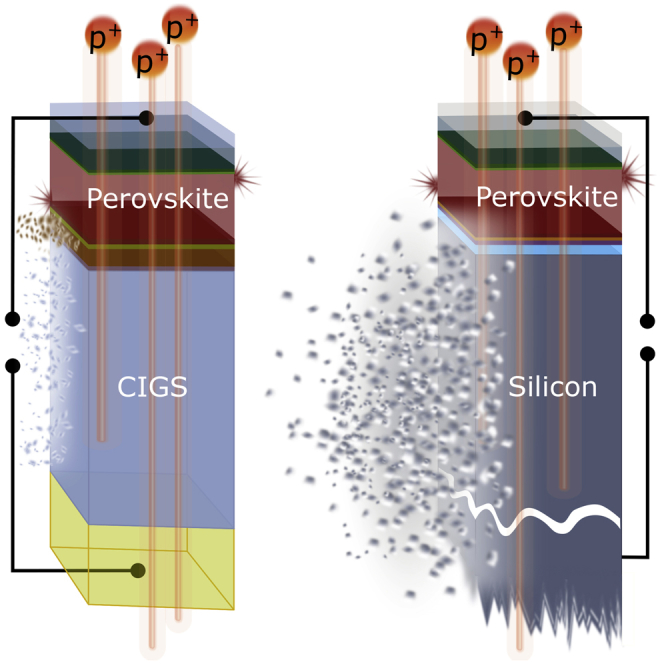

## Teaser

We propose and test monolithic perovskite/CIGS tandem solar cells for readily stowable, ultra-lightweight space photovoltaics. We design *operando* and *ex situ* measurements to show that perovskite/CIGS tandem solar cells retain over 85% of their initial power-conversion efficiency after high-energy proton irradiation. While the perovskite sub-cell is unaffected after this bombardment, we identify increased non-radiative recombination in the CIGS bottom cell and nickel-oxide-based recombination layer. By contrast, monolithic perovskite/silicon-heterojunction cells degrade to 1% of their initial efficiency due to radiation-induced defects in silicon.

## Introduction

Multijunction solar cells that combine complementary absorber materials to selectively harvest the available solar spectrum with minimal thermalization losses power modern energy demanding satellites, spacecraft, and exploration rovers.^1^ While terrestrial photovoltaic (PV) systems require high-power-area (W/m^2^) ratios, space PV systems also require high specific power (W/g) to minimize the stowed volume, weight, inertia, and atmospheric drag of the spacecraft.^2^ In addition, the cost of space PV modules ($/W) is becoming increasingly important given the growing demand for smaller, cheaper satellites^3^ and the emerging privatization of space exploration,^4^ both of which are revolutionizing space economics. Furthermore, only lower costs will allow large-scale space explorations, including planned habitats on the Moon and Mars.^4^ Triple- and quadruple-junction solar cells comprised of GaInP/GaAs/Ge or AlInGaP/AlInGaAs/InGaAs/Ge absorbers are today’s state-of-the-art commercially available systems, reaching power-conversion efficiencies of 32%^5^^,^^6^ under space solar illumination conditions (AM0). However, slow epitaxial absorber growth and high material costs render such III-V-based multijunction solar cells expensive and their mass production challenging.^7^ Less expensive space-tested single-junction technologies based on crystalline silicon (c-Si),^8^^,^^9^ Cu(In,Ga)Se_2_ (CIGS),^10^ halide perovskites,^11^, ^12^, ^13^ or organic absorbers^14^ do not meet the performance requirements of sufficiently high W/g and W/m^2^ to compete with the III-V multijunction technologies. Compositionally engineered perovskites, with a band gap (E_G_) of 1.6–1.8 eV can be processed on top of c-Si (E_G_ = 1.1 eV)^15^, ^16^, ^17^, ^18^, ^19^ and CIGS (E_G_ ∼1.1 eV)^20^, ^21^, ^22^ absorbers, enabling monolithic tandem solar cells with efficiencies that surpass the limiting values of individual sub-cells. The first technology is close to commercialization for terrestrial applications,^23^ while the latter technology can be processed on flexible foils to enable high W/g and W/m^2^ values at low cost. Thus, perovskite-based multijunction PV has the potential to be a disruptive technology both on the Earth and in Space.

A crucial requirement for adoption is that the cells can withstand the harsh radiation environment of space without additional engineering solutions that add cost and compromise performance metrics. Accelerated by coronal mass ejections and solar flares, solar energetic particles consist mainly of protons (p^+^) and electrons with kinetic energies ranging from keV to GeV.^24^ High-energetic protons are about two orders of magnitude more damaging than highly energetic electrons.^12^ Moreover, protons with energies above 1 MeV cannot be easily shielded and consequently damage electronic devices,^24^ such as solar cells, which eventually leads to device failure. Promising test results from perovskite-based single-junction solar cells have revealed that devices under proton irradiation retained over 90% of their initial performance even after high proton fluences of 10^12^ p^+^/cm^2^ ^13^ and 10^14^p^+^/cm^2^ ^12^ with proton energies of 0.05–68 MeV.^11^, ^12^, ^13^^,^^25^^,^^26^ However, monolithic tandem solar cells are connected in series and, hence, radiation-induced damage in just one of the sub-cells can degrade the performance of the entire tandem solar cell. Therefore, to validate these technologies, studies are required on the entire tandem systems during operation. Here, we reveal the suitability of state-of-the-art monolithic perovskite/CIGS tandem solar cells to power satellites and spacecraft by testing their radiation hardness *in operando* under 68 MeV proton (p^+^) irradiation.

## Results

### Perovskite/CIGS and Perovskite/Silicon Tandem Solar Cells

**Figure 1:**
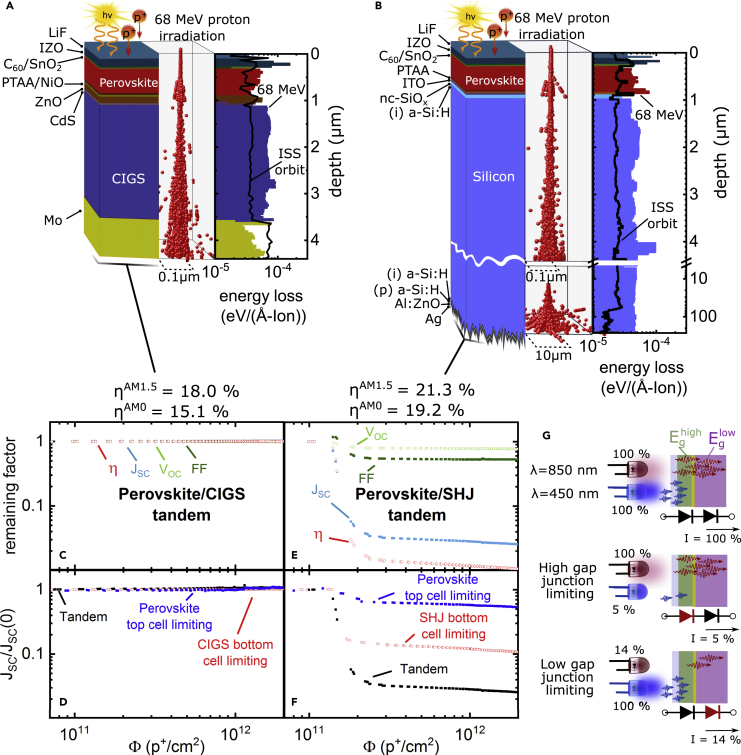
Probing the Radiation Hardness of Perovskite/SHJ and Perovskite/CIGS Tandem Solar Cells *In Operando* during Proton Irradiation (A and B) 3D scatter plots of the straggling of 68 MeV protons within the perovskite/CIGS (A) and perovskite/SHJ (B) tandem solar cells. The corresponding energy loss of the incident 68 MeV protons to recoils is plotted as a function of depth based on SRIM simulations with a total of 5 × 10 ^7^ protons. The damage of a real space environment at the orbit of the international space station (ISS) is shown as black line considering polyenergetic and omnidirectional proton irradiation (see Supplemental Information for further details). (C and E) *Operando* measurements of V_OC_, J_SC_, FF, and η of the investigated perovskite/CIGS (C) and perovskite/SHJ (E) tandem solar cell as a function of the accumulated proton dose Φ. All values are normalized to their initial value. The proton energy amounted to 68 MeV. (D–G) Normalized short-circuit current of perovskite/CIGS (D) and perovskite/SHJ (F) tandem solar cell under illumination with NIR (λ = 850 nm) and blue LEDs (λ = 450 nm) that were alternatingly set to either 100% or 5/14% ( see Supplemental Information for further details) to mimic current matching under AM0 or forcing one sub-cell into limitation as illustrated in (G).

The investigated perovskite/CIGS and perovskite/silicon tandem solar cells utilize triple cation perovskite absorber layers [Cs_0.05_(MA_0__.__17_FA_0__.__83_)_0.95_]Pb(I_0.83_Br_0.17_)_3_ with a band gap of E_G_ = 1.62 eV (Figures 1A and 1B). In both cases, we employ an inverted p-i-n configuration and sandwich the perovskite absorber between poly[bis(4-phenyl)(2,4,6-trimethylphenyl)amine] (PTAA) and C_60_ layers that act as hole- and electron-selective layers, respectively. To avoid the influence of oxygen and moisture^18^ all tandems were air-to-N_2_ encapsulated using a radiation-hard quartz substrate, which leads to additional reflection losses of ∼7% that could be ultimately removed using more suitable encapsulation techniques. The stabilized efficiency and power output of the quartz-encapsulated perovskite/CIGS solar cells here, thus, amounts to 18% and ∼180 W/m^2^, respectively, under irradiation with a terrestrial solar spectrum AM1.5G (1,000 W/m^2^). The stabilized power output increases to ∼202 W/m^2^ with an efficiency of 15.1% under space AM0 spectral conditions (1,350 W/m^2^). The perovskite/CIGS tandem solar cells have a combined active layer thickness of 4.38 μm and a very low specific weight of just 2.8 mg/cm^2^, yielding an excellent specific-power of 7.4 W/g. We note that these values do not account for commonly employed encapsulation glasses and substrates. Assuming a 25-μm thick substrate and encapsulation foil often used for flexible CIGS and perovskite solar cells,^27^ the specific power is 2.1 W/g, a factor of ∼3 times larger than those of typically used GaInP/GaAs/Ge absorbers at 0.8 W/g^5^ and expected improvements in efficiency will increase this factor further. For the monolithic perovskite/silicon tandem solar cells, we utilize a rear emitter c-Si (n) silicon heterojunction (SHJ) with planar front and textured backside. The stabilized efficiency and power output of the quartz-encapsulated perovskite/SHJ cells reaches 21.3% and ∼213 W/m^2^, respectively, under AM1.5G irradiation, increasing to ∼257 W/m^2^ with an efficiency of 19.2% under AM0. The perovskite/SHJ tandem solar cell is based on an active layer with a combined thickness of 261.5 μm and a specific weight of 61 mg/cm^2^, yielding a specific-power of 0.42 W/g (excluding encapsulation glass), which is comparable to the triple-junction technologies in terms of specific power while also promising much lower power module costs ($/W), albeit without the flexible form factor that CIGS and perovskites offer.

### Proton-Irradiation-Induced Damage Profile

We employ 68 MeV proton irradiation to represent a key problematic energy range that will penetrate through sufficiently inexpensive encapsulating engineering solutions, standardly employed to shield low-energy protons (E < 1 MeV), including the quartz substrate used here. To ascertain damage in the layer stacks due to electronic and nuclear scattering, we used a Monte Carlo based simulation of the stopping and range of ions in matter (SRIM).^28^ Electronic scattering of the incident proton ionizes the target material and can lead to the disruption of C–H and N–H bonds in the organic and hybrid perovskite layers in the device stacks.^29^^,^^30^ Nuclear scattering of the incident proton causes the target nuclei to recoil and be displaced, which instigates a cascade of damage events that generate defects in the material. In Figures 1A and 1B, we plot the simulated straggling as well as the energy transferred to the recoiling nuclei that is typically used as a measure for the degradation of PV parameters.^31^ In both the investigated perovskite/SHJ and perovskite/CIGS stacks, we observe a uniform damage profile throughout the layer stacks, therefore, allowing us to probe the impact of irradiation on the entire tandem stack with this proton energy. We also compare the damage profile of our monoenergetic and monodirectional 68 MeV proton irradiation to the damage expected from poly-energetic omnidirectional proton irradiation in the orbit of the International Space Station (ISS) (Figures 1A and 1B, black line; see Figure S1 in the Supplemental Information for details), revealing a similarly uniform profile. Thus, these experiments replicate a real space environment in which both sub-cells and all involved interlayers are damaged comparably. Non-uniform damage confined to the topmost layers from low-energy proton testing (E< 1 MeV) will not be seen in orbit,^32^ while as we identify later, some of the most problematic layers are buried recombination contacts. Our SRIM simulations further suggest that the proton dose of 2 × 10^12^p^+^/cm^2^ is equivalent to the accumulated dose on the tandem solar cells after more than 50 years in an ISS orbit (see Supplemental Information for details).

### Probing the Radiation Hardness *In Operando*

To assess the radiation hardness of the investigated tandem solar cells, we tracked *in operando* the evolution of the PV parameters during 68 MeV proton irradiation as a function of the accumulated proton dose Φ. Using an incident fluence of ∼7 × 10^8^ p^+^/cm^2^/s the highest dose of Φ = 2 × 10^12^ p^+^/cm^2^ was reached after ∼1 h of operation. For the *operando* measurements, the active area of 0.81 cm^2^ was homogeneously illuminated by two high-intensity LEDs equivalent to ∼¼ AM0 with wavelengths tailored to selectively illuminate the high gap or the low gap sub-cells, respectively ($\lambda_{LED}^{1}$ = 450 nm and $\lambda_{LED}^{2}$ = 850 nm). In contrast to conventional white light LEDs, this allowed us to mimic current matching between the two sub-cells equivalent to them being under AM1.5G or AM0 conditions (Figure 1G, top). In Figure 1C, we display the evolution of the open-circuit voltage (V_OC_), fill factor (FF), short-circuit current density (J_SC_), and power-conversion efficiency (η) of the perovskite/CIGS tandem solar cell, with each metric normalized to its initial value (see Figures S3 and S4 for un-normalized JV characteristics). The *operando* data reveal only minor degradation in V_OC_ to about 99% of its initial value and no significant degradation in FF, J_SC_, or η for a proton dose of 2 × 10^12^ p^+^/cm^2^. By contrast, we observe that the perovskite/SHJ tandem solar cell (Figure 1E) degrades rapidly to an efficiency of only 1% of its initial value after an accumulated proton dose of only Φ ∼10^11^ p^+^/cm^2^. Notably, the degradation of the perovskite/SHJ tandem is dominated by the J_SC_ that decreases to about 2% of its initial value, and the performance parameters do not recover after removing the proton irradiation (Figure S2).

To further investigate each sub-cell during proton irradiation, we periodically decreased the intensity of one LED to ∼5% or 14% of its output power such that one of the sub-cells limits the overall current (Figure 1G). In the case of the perovskite/SHJ tandem solar cell limited by the SHJ bottom cell, the J_SC_ decreases rapidly (Figure 1F), strongly suggesting that the degradation of the perovskite/SHJ tandem is dominated by the SHJ sub-cell. However, the data also suggest some losses within the perovskite sub-cell. By contrast, neither the perovskite nor the CIGS sub-cell degrades under the same irradiation dose in the case of the perovskite/CIGS tandem solar cell (Figure 1D).

### Radiation-Hard Perovskite/CIGS Tandem Solar Cells

**Figure 2:**
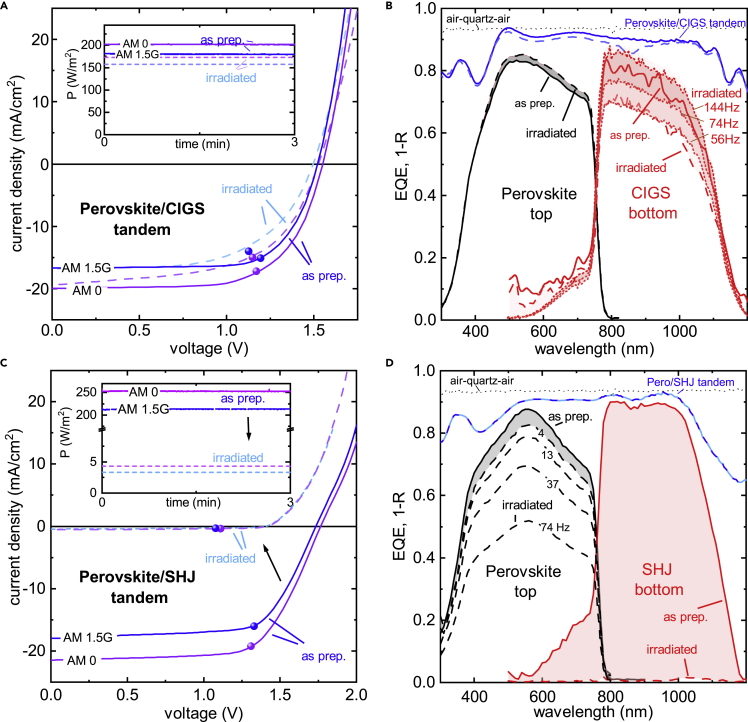
Proton-Irradiated Perovskite/SHJ and Perovskite/CIGS Tandem Solar Cells (A and C) Current-voltage characteristics of as-prepared (solid lines) and proton irradiated (dashed lines, Φ = 2 × 10 ^12^ p^+^/cm^2^, E_p_ = 68 MeV) perovskite/CIGS (A) and perovskite/SHJ (C) tandem solar cell under AM1.5G and AM0 illumination. The full circles indicate the mean maximum power point (MPP), and the inset depicts the power output at MPP as a function of time. (B and D) External quantum efficiency of the perovskite and the CIGS sub-cell (B) before (solid lines) and after proton irradiation (dashed lines). The EQE was measured using a chopper frequency of 74 Hz and appropriate LEDs to light bias the tandem. In the case of the CIGS bottom cell, EQE measurements were also performed employing higher chopper frequencies and stronger light biasing from a halogen lamp equipped with appropriate filters as indicated. In case of the perovskite/SHJ tandem (D), the irradiated perovskite top cell was also measured employing lower chopper frequencies as indicated. In both cases, the reflection of the tandem solar cells is shown by the blue solid (as-prepared) and dashed (irradiated) lines. The dotted lack line depicts the reflection of the used air-quartz-air encapsulation.

In order to understand the behavior of the irradiated cells, the devices were characterized prior to and after the proton irradiation. We note that the post-irradiation measurements were performed after the activity of generated short-living isotopes in the irradiated samples dropped to a safe level of less than 10^3^ Bq (∼10 days of storage). In Figure 2A, we show current-voltage (JV) measurements of the perovskite/CIGS tandem under AM0 and AM1.5G conditions taken prior to and after 68 MeV proton irradiation. Notably, the power output remains high, and the perovskite/CIGS tandem solar cell retains ∼85% of its initial performance under AM0 illumination, with the power output decreasing from ∼202 to ∼173 W/m^2^. Under AM1.5G illumination, the perovskite/CIGS tandem solar cell retains over ∼83% of its initial performance (see Table S1 for parameters). The JV curves show that the small but detectable radiation-induced performance losses primarily originate from a reduction in V_OC_ and FF. Notably, J_SC_ remains high. The findings are corroborated by measuring the spectral response of both sub-cells using appropriate light and voltage biases (Figure 2B). The external quantum efficiency (EQE) of the perovskite sub-cell increases slightly, from a maximum value of 83% before irradiation to 85% after proton irradiation, which we discuss further below. On the other hand, the EQE of the CIGS sub-cell decreases from a maximum value of 84% before irradiation (solid red line) to 77% after proton irradiation (red dashed line) when measured under identical light biasing conditions. By employing stronger light biasing and higher chopper frequencies, however, the CIGS sub-cell EQE could be recovered up to a maximum value of 86% (red dotted lines). We propose that this dependence on the frequency and light biasing is a combination of radiation-induced trap states and a low shunt resistance of the CIGS bottom cell (see Figures S19 and S4 for JV characteristics in the dark and under 14% 850 nm, 100% 450 nm LED illumination), and we also note that spectral response measurements of multijunction cells are challenging after particle irradiation or when one sub-cell under test features non-ideal properties.^33^^,^^34^ This is exemplified by the fact there is no loss in EQE in identically prepared and irradiated CIGS single-junction solar cells (Figure S11). The radiation-induced trap states in the perovskite/CIGS tandem solar cell lead to a decrease in the open-circuit voltage of ΔV_OC_ = 0.02 V.

By contrast, the JV measurements of the perovskite/SHJ cell shown in Figure 2C reveal a large decrease in all PV parameters upon proton irradiation. While the V_OC_ and FF decrease by ∼20% relative to their initial value, it is the short-circuit current density *J*_SC_ that shows a dramatic reduction from 21.5 to only 0.3 mA/cm^2^ (under AM0 conditions) and, consequently, the stabilized power output is reduced from ∼257 to only 4 W/m^2^. These findings are also reflected in the changes of the maximum EQE of the SHJ sub-cell (Figure 2D), which drops from 90% to 1.5% after proton irradiation. This shows unambiguously that the SHJ bottom cell is heavily damaged, consistent with previous works on single-junction c-Si technologies,^9^^,^^35^, ^36^, ^37^ with the SHJ cell thereby limiting the overall perovskite/SHJ tandem solar cell. However, we observe that the maximum EQE of the perovskite sub-cell also drops from 87% to 50%, though the maximum EQE can be recovered to 82% as the optical chopper frequency for the measurement is reduced from 74 to 4 Hz. We propose that this frequency dependence is an artifact generated by the heavily damaged SHJ sub-cell, which limits the overall current and, thus, the spectral response of the perovskite sub-cell despite using appropriate light biases during fast measurements, but this effect can be mitigated during slower (lower frequency) measurements. Indeed, the photoconductivity of silicon can be sensitive to the emptying and filling of shallow and deep traps, occurring on timescales between 10^−2^ and 10^1^ s, respectively.^38^ Further evidence from selectively probed photoluminescence measurements that allows one to exclude the radiation-induced degradation of the perovskite sub-cell in both the perovskite/CIGS and perovskite/SHJ tandem is discussed further below and summarized in Figure 5.

### Optical Characterization of the Radiation-Induced Damage

**Figure 3:**
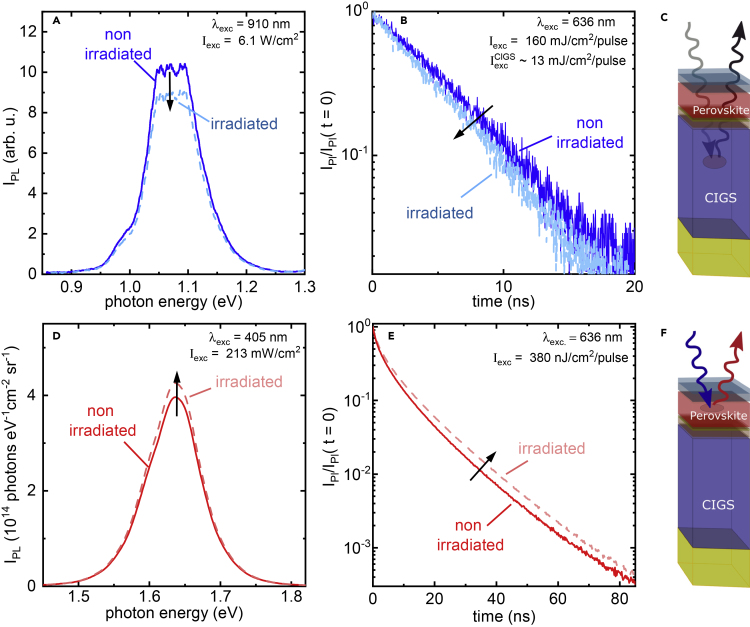
Identification of Radiation-Induced Recombination Pathways in Perovskite/CIGS Tandem Solar Cells after Proton Irradiation (A–C) Photoluminescence spectra (A) and decay (B) of the non-irradiated and irradiated CIGS bottom absorber. As sketched in (C), selective excitation in the CIGS layer was performed through the perovskite top absorber employing either a NIR cw laser at λ = 910 nm (in A) or a pulsed λ = 636 nm laser (in B) at a fluence of 160 mJ/cm^2^ of which 13 mJ/cm^2^ are absorbed within the CIGS in combination with appropriate long-pass filters to detect the emission. (D–F) Photoluminescence spectra (D) and decay (E) of the non-irradiated and irradiated perovskite top absorber. Excitation was performed using cw 405 nm (in D) or pulsed 636 nm (in E) illumination at 380 nJ/cm^2^, as shown in (F).

To assess the V_OC_ losses of the perovskite/CIGS tandem solar cell, we selectively probed both sub-cells using steady-state and time-resolved photoluminescence (PL and TRPL) measurements that are sensitive to unwanted non-radiative recombination power loss pathways, for example, due to the presence of radiation-induced recombination centers. These optical measurements were carried out on proton-irradiated regions and non-irradiated regions on the same devices. The PL from the CIGS sub-cell, measured by selectively photo-exciting the CIGS layer using a NIR excitation at λ = 910 nm that passes through the perovskite top cell as displayed in Figure 3C, shows a lower PL intensity than before irradiation (Figure 3A), consistent with the presence of radiation-induced defects within the CIGS sub-cell. This is corroborated by a slightly faster TRPL decay after irradiation, which we measure using a pulsed λ = 636 nm excitation and appropriate long-pass filters to selectively detect PL from the CIGS sub-cell (Figure 3B). The perovskite top-cell PL and TRPL were probed using excitation wavelengths of λ = 405 nm and λ = 636 nm, respectively, that are predominantly absorbed in the perovskite sub-cell, as illustrated in Figure 3F. Surprisingly, we observe a slight increase in PL intensity (Figure 3E) and a prolongation of the TRPL decay (Figure 3D) after proton irradiation. Hence, the collective optical and device data suggest that the degradation of the perovskite/CIGS tandem under proton irradiation originates from damage in the CIGS sub-cell. We performed similar optical measurements on perovskite/SHJ tandem solar cells and found a vast decrease of PL intensity paired with a quenched TRPL decay in the SHJ sub-cell after proton irradiation (Figure S9), reaffirming that proton irradiation primarily causes damage in the SHJ bottom cell.

### Photoluminescence Mapping of the Perovskite Sub-cell

**Figure 4:**
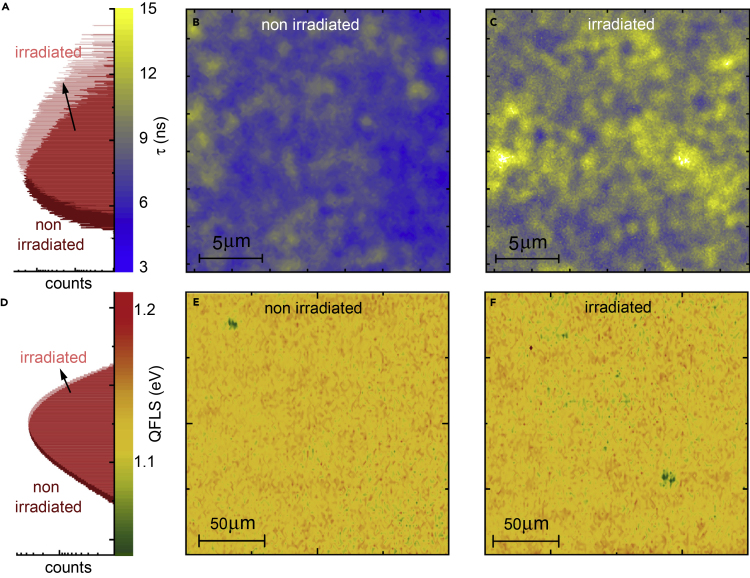
Photoluminescence Lifetime and Quasi-Fermi-Level-Splitting Mapping of the Perovskite (A–C) (A) Photoluminescence lifetime histogram and (B and C) TRPL lifetime maps of the perovskite top absorber in the as-prepared and proton-irradiated perovskite/CIGS tandem solar cell under excitation with a 636 nm pulsed laser (5 MHz repetition rate, 380 nJ/cm^2^/pulse fluence). Lifetimes were extracted using single-exponential fitting. (D–F) (D) QFLS histogram and (E and F) QFLS maps of the perovskite top absorber in the as-prepared and proton-irradiated perovskite/CIGS tandem solar cell measured under 405 nm cw laser illumination with an intensity equivalent to 1 sun (see Supplemental Information for details).

In contrast to single-crystalline absorbers, spatial heterogeneities in composition, crystallinity, defect density, and optoelectronic properties pervade solution-processed perovskites.^39^ To assess the impact of proton irradiation on the local properties of the perovskite sub-cell, we employed confocal photoluminescence lifetime mapping with high spatial resolution (∼300 nm). In Figure 4B, we depict a lifetime map of a non-irradiated cell under pulsed (∼10 sun equivalent) excitation, revealing an average lifetime of τ = 7.5 ± 1.0 ns and heterogeneous features at length scales on the order of 1 μm, a length scale about 2–3 times larger compared to typical grain sizes estimated from scanning electron micrographs.^20^ In accordance with TRPL data shown in Figure 3E, we identify a prolongation of the mean lifetime after irradiation to τ = 9.9 ± 1.3 ns accompanied by a slight increase in heterogeneity (Figure 4C), as seen in the right-skewed section of the histogram (Figures 4A and S13C). As the PL decay of halide perovskites is strongly quenched when sandwiched between charge selective contacts, the observed prolongations likely arise from radiation-induced changes at these interfaces, which we discuss further below.^40^

The spontaneous emission of photons from a direct semiconductor is, according to Würfels’ generalized Planck law,^41^ a function of the chemical potential of the non-equilibrium charge carrier concentration. This concentration corresponds to the quasi-Fermi-level splitting (QFLS) of photo-excited electrons and holes, a quantity that translates to open-circuit voltage in a solar cell. By probing the absolute PL spectrum, we can thus extract the QFLS.^42^ Here, we employ absolute hyperspectral PL imaging with high spatial resolution (∼1 μm) to record the local QFLS of the perovskite sub-cell under equivalent excitation carrier densities to 1 sun (AM1.5G, see Supplemental Information for further details). In Figures 4E and 4F, we show QFLS maps of an irradiated and a non-irradiated device, respectively, along with their associated number histograms in Figure 4D (see Figure S14 for the corresponding PL maps and local PL spectra of selected locations). We find that there is an insignificant shift of the mean QFLS from 1.120 ± 0.022 eV (non-irradiated) to 1.124 ± 0.024 eV (irradiated), reiterating the excellent radiation hardness of the perovskite sub-cell.

### Origin of Radiation-Induced V_OC_ Losses

**Figure 5:**
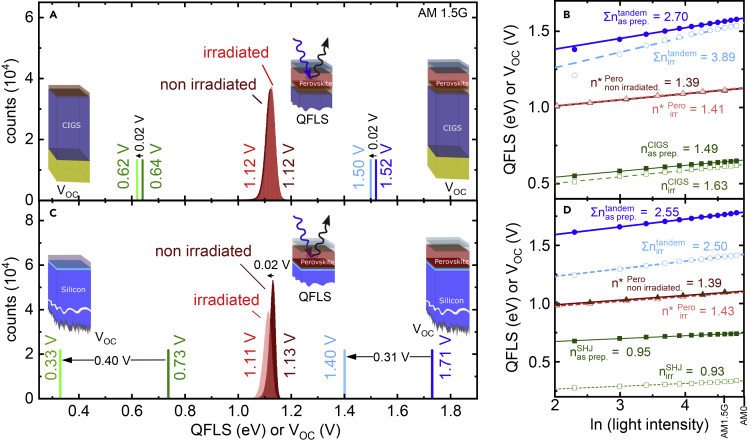
Radiation-Induced V_OC_ Losses in Perovskite/CIGS and Perovskite/SHJ Tandem Solar Cells (A and C) Comparison of perovskite top cell QFLS statistics with the V _OC_ of perovskite/CIGS (A) and perovskite/SHJ (C) tandem and identically prepared CIGS and SHJ single-junction solar cells before and after irradiation, respectively. (B and D) V_OC_ as a function of light intensity for as-prepared and proton-irradiated perovskite/CIGS (B) and perovskite/SHJ (D) tandem solar cells, as well as CIGS and SHJ single-junction solar cells, respectively. Open and closed triangles depict the QFLS of the perovskite sub-cell as a function of excitation fluence for the perovskite/CIGS and perovskite/SHJ tandem solar cells. n^∗^ denotes the internal ideality factor derived from Suns-QFLS statistics.

To connect these results with the device measurements, we compare in Figure 5A the perovskite sub-cell QFLS to measured device V_OC_ values of the perovskite/CIGS tandem and a CIGS single-junction cell; both measured before and after proton irradiation. The V_OC_ loss of the perovskite/CIGS tandem of Δ V_OC_ = 0.02 V matches the V_OC_ loss observed in a CIGS single-junction device. This comparison confirms that the V_OC_ losses in the tandem arise from losses in the CIGS bottom cell, consistent with the decrease in PL intensity (cf. Figure 3A), reiterating the resilience of the perovskite sub-cell to proton irradiation. To investigate this further, we perform intensity-dependent-V_OC_ measurements (Suns-V_OC_) of the perovskite/CIGS tandem and the CIGS single-junction cells and extract the ideality factor *n* that is indicative of the dominant recombination mechanisms, i.e., *n* = 1 for ideal band-to-band recombination, *n* = 2 in the presence of deep recombination centers causing Shockley-Read-Hall recombination and *n* < 1 for Auger recombination under high injection conditions.^43^ The Suns-V_OC_ slope of a monolithic tandem solar cell approximately equals the sum of the sub-cell ideality factors, $S=\sum_{i}n_{i}k_{B}T_{i}\approx k_{B}T\sum_{i}n_{i}$, assuming the individual sub-cells are at the same temperature of T = 300 K as set using a temperature-controlled stage (see Supplemental Experimental Procedures for details). Here k_B_ is the Boltzmann constant, and T is the temperature. As shown in Figure 5B, we estimate an $\sum_{i}n_{i}$ of 2.7 and 3.89 on as-prepared and proton-irradiated perovskite/CIGS tandem solar cells, respectively. This significant increase in the summed ideality factor suggests the presence of radiation-induced recombination centers in at least one of the sub-cells. Intensity-dependent-V_OC_ measurements on identically prepared and irradiated CIGS single-junction solar cells indicate an increase in the ideality factor from 1.43 to 1.69 after proton irradiation. This observation indicates the presence of radiation-induced recombination centers, which is consistent with the observed decrease in V_OC_ and PL intensity (see Figures 2A and 3A). However, this increase in ideality factor is still somewhat smaller compared to the increase observed in the perovskite/CIGS tandem solar cell, which indicates the existence of an additional recombination pathway in the perovskite/CIGS tandem leading to FF loss after irradiation that is not present in the equivalent single-junction cells. We performed absolute PL measurements of the perovskite sub-cell while varying the excitation intensity, which allows us to extract the intensity-dependent QFLS (Suns-QFLS). Following Caprioglio et al.,^44^ an internal ideality factor, which is dominated by the perovskite bulk and mostly unaffected by interfacial losses, can be derived. As shown in Figure 5B, the internal ideality of the perovskite sub-cell increases slightly from 1.39 to 1.41. This again highlights the minimal impact of radiation-induced defects on the perovskite bulk properties.

To generate an overall picture, we compare insights derived on perovskite/CIGS tandems to the perovskite/SHJ case, which we show in Figure 5C (see similar analyses to produce these values in Figures S5–S7). Similar to the perovskite on CIGS, the mean QFLS of the perovskite on SHJ remains high and reduces only slightly from 1.127 ± 0.013 eV (non-irradiated) to 1.109 ± 0.016 eV (irradiated). The QFLS reduction of ΔQFLS = 0.02 eV is an order of magnitude smaller than the V_OC_ loss observed in the perovskite/SHJ tandem of ΔV_OC_ = 0.31 V, clearly showing that the perovskite/SHJ tandem is limited by the SHJ bottom cell after irradiation. Surprisingly, we observe only minor changes in the ideality factor of the perovskite/SHJ tandem solar cell with irradiation (Figure 5D). In as-prepared devices, we estimate $\sum_{i}n_{i}$ = 2.55 for the perovskite/SHJ tandem and n_SHJ_ = 0.95 for an identically prepared SHJ single junction, the latter being due to Auger recombination typically dominating recombination in silicon cells.^45^ The value remains <1 after irradiation (n_SHJ_ = 0.93), indicating that Auger processes still dominate the recombination mechanisms. In the perovskite/SHJ tandem, we estimate $\sum_{i}n_{i}$ = 2.50, which is close to the value before irradiation, and once again corroborates the radiation hardness of the perovskite top cell. This conclusion is further supported by Suns-QFLS measurements of the perovskite sub-cell that indicate an unchanged internal ideality factor of 1.39 (non-irradiated) and 1.41 (irradiated).

## Discussion

Taking both the electrical and optical characterization results into account, it is clear that proton-irradiation-induced trap states are formed within the CIGS bottom cell. Consequently, the CIGS bottom cell features a reduced V_OC_ and FF, which leads to reduced performance of the overall tandem. From the FF and V_OC_ losses of the CIGS single junction, we estimate that this constitutes ∼60% of the performance loss observed in the perovskite/CIGS tandem solar cell after proton irradiation. Thus, the radiation hardness could be improved further by utilizing a more radiation-hard bottom cell, such as Cu_2_ZnSn(S,Se)_4_ (CZTSSe). Recent experiments revealed a promising radiation hardness for CZTSSe outperforming CIGS absorbers by a factor of two, albeit with their power-conversion efficiency still being a factor of two smaller than those of typical CIGS single-junction devices.^10^ Other promising candidates are low-band-gap perovskite cells based on Sn-Pb mixtures^46^, ^47^, ^48^; however, their radiation hardness has not been investigated yet.

We have further established an additional recombination pathway that impacts FF and constitutes ∼40% of the performance loss of the entire perovskite/CIGS tandem solar cell. Our optical and electrical measurements on the perovskite/SHJ tandem solar cell, as well as previously published perovskite single-junction results,^13^ allow us to exclude degradation of the perovskite absorber layer itself. The unaffected values for QFLS and internal ideality factor of the perovskite sub-cell in the perovskite/CIGS tandem further suggest that the perovskite bulk is largely unaffected while pointing toward increased interfacial recombination. In contrast to perovskite/SHJ and perovskite single-junction solar cells, perovskite/CIGS tandem solar cells, require a 10-nm thick NiO layer conformally grown by plasma-assisted atomic layer deposition (ALD) between ZnO and PTAA to mitigate shunting of the perovskite top cell on the rough CIGS bottom cell.^20^ Proton, γ- and UV-irradiation is known to induce defects in NiO that increase its conductivity while also leading to conversion from p-type to n-type behavior.^49^, ^50^, ^51^ This deteriorates the energetic alignment between PTAA and ALD NiO, leading to less efficient extraction of charge carriers, thereby affecting FF and maximum power point (MPP) of the perovskite top cell. The luminescence of perovskite absorbers sandwiched between two selective contacts is known to be heavily influenced by surface recombination and charge carrier extraction into the selective contacts.^40^^,^^52^ Changes in energetic alignment of PTAA/NiO will hence simultaneously impact charge extraction & luminescence properties explaining the observed PL enhancements and TRPL prolongations of the perovskite sub-cell (reduced PL quenching) in the perovskite/CIGS tandem after proton irradiation (see Figures 3 and 4); in perovskite/SHJ tandem solar cells that do not require an ALD NiO interlayer, we observe a slight reduction of the PL and shortening of the TRPL lifetime of the perovskite sub-cell (Figures S7 and S8). We note that the problematic NiO layer, as well as the degradation in the CIGS and SHJ bottom cells, would have been overlooked using low-energy proton irradiation (E < 1 MeV) that only impinges the topmost layers.

All in all, the perovskite/CIGS tandems possess a high radiation hardness and retain over ∼85% of their initial performance even after 68 MeV proton irradiation and a dose of Φ = 2 × 10^12^ p^+^/cm^2^, which is comparable to conventional GaInP/GaAs/Ge absorbers that retain ∼82% of their initial performance at an identical displacement damage.^53^ These irradiation conditions correspond to more than 50 years in space at the ISS orbit, and consequently, perovskite-based multijunction PV has the potential to become a disruptive space PV technology. Assuming further improvements in power-conversion efficiency approaching η = 30%, the specific-power of perovskite/CIGS would be increased to 14 W/g (4 W/g if assuming a 25 μm thick substrate and encapsulation foil). Both values vastly exceed those of conventional used GaInP/GaAs/Ge absorbers at 0.8 W/g.^5^ Perovskite/CIGS tandem solar cells are currently optimized for terrestrial PVs and have shown rapid progress,^20^^,^^22^ with recent demonstration of flexible perovskite/CIGS tandem solar cells,^54^ thereby rendering the above outlook achievable. Promising results by Barbé et al.^55^ and Brown et al.^56^ further suggest that perovskite single-junction devices can tolerate extreme temperature changes and operate well under low-intensity and low-temperature environments, both of which can be found in some space environments, albeit this needs to be verified for perovskite/CIGS tandems and their more complex layer stack. Thermo-mechanical stress from temperature cycling, diurnal cycles, and/or partial shading can cause additional degradation pathways, conditions that are equally present in space, high-altitude, and terrestrial environments. We therefore encourage dedicated investigations of emerging and established PV technologies using our described operando methodology to decouple degradation of the individual sub-cells by sequentially forcing one sub-cell into limitation conditions.

### Conclusions

In summary, we have evaluated perovskite/CIGS and perovskite/SHJ tandem solar cells for their suitability to withstand the harsh radiation environment in space using tailored *in-operando* and *ex-situ* measurements during and after high-energetic proton irradiation. Our results show that perovskite/SHJ tandem solar cells degrade severely to 1% of their initial efficiency while perovskite/CIGS tandem solar cells retain over 85% of their initial efficiency under AM0 solar illumination even after 68 MeV proton irradiation at a dose of 2 × 10^12^ p^+^/cm^2^. Using high spatial resolution photoluminescence microscopy, we further showed that the open-circuit voltage potential of the perovskite top cell is unaffected after high-dose proton irradiation. Combining insights from selectively probed photoluminescence and intensity-dependent V_OC_ measurements, we isolated the layers responsible for the efficiency losses of the tandem solar cells. We find that the losses primarily arise from increased recombination in the CIGS bottom cell, and the atomic layer deposited nickel-oxide-based recombination contact. With a radiation hardness that rivals state-of-the-art III-V semiconductor-based space PV, our work identifies perovskite/CIGS tandem solar cells that can be processed on flexible foils, as a cheap, readily stowable and ultra-lightweight space PV technology with power-to-weight and power-to-cost ratios surpassing those of state-of-the-art III-V semiconductor-based triple- and quadruple-junction absorbers. While our proton irradiation mimics the damage in space and high-altitude environments, our insights and *in operando* methodology provide a new perspective to improve and investigate the long-term stability of emerging tandem solar cell technologies for terrestrial, high-altitude, and space applications.

## Experimental Procedures

Full details of experimental procedures can be found in the Supplemental Information.

## Supplemental Information

Document S1. Figures S1–S15, Table S1, Note S1, Supplemental Experimental Procedures, and Supplemental References

Document S2. Article plus Supplemental Information

## Footnotes

Supplemental Information can be found online at https://doi.org/10.1016/j.joule.2020.03.006.
