# Supplementary material for: Proton Radiation Hardness of Perovskite Tandem Photovoltaics
Source: Joule. 2020 May 20;4(5):1054–69. doi: 10.1016/j.joule.2020.03.006 (PMC7238692; doi:10.1016/j.joule.2020.03.006)
Supplement: Document S1. Figures S1–S15, Table S1, Note S1, Supplemental Experimental Procedures, and Supplemental References [file mmc1.pdf]

JOUL, Volume *4*

Supplemental Information

Proton Radiation Hardness of Perovskite Tandem Photovoltaics

Felix Lang, Marko Jošt, Kyle Frohna, Eike Köhnen, Amran Al-Ashouri, Alan R. Bowman, Tobias Bertram, Anna Belen Morales-Vilches, Dibyashree Koushik, Elizabeth M. Tennyson, Krzysztof Galkowski, Giovanni Landi, Mariadriana Creatore, Bernd Stannowski, Christian A. Kaufmann, Jürgen Bundesmann, Jörg Rappich, Bernd Rech, Andrea Denker, Steve Albrecht, Heinz-Christoph Neitzert, Norbert H. Nickel, and Samuel D. Stranks

Supplemental Data

Proton fluence at ISS orbit

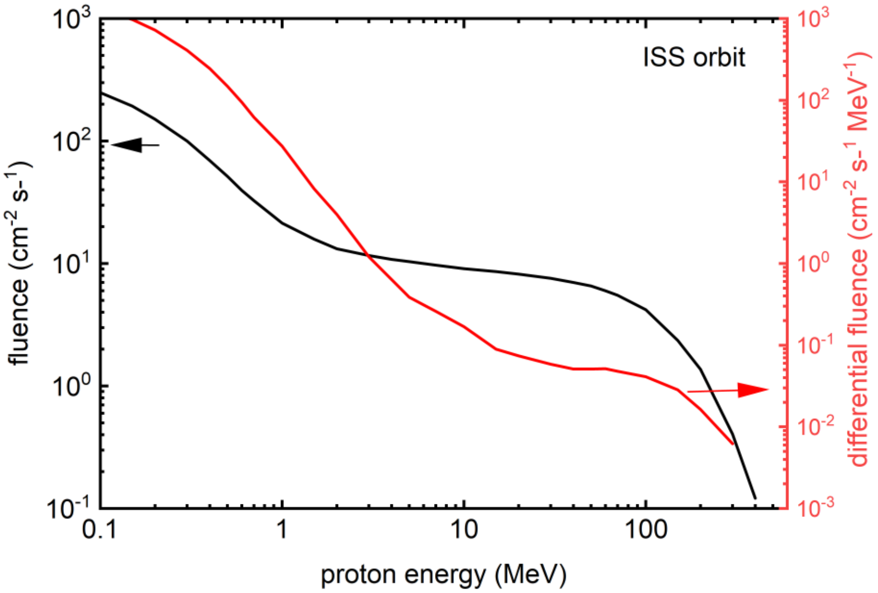

Figure S1: Proton spectrum in ISS orbit: Fluence and differential fluence of the proton spectrum in an ISS orbit as simulated using SPENVIS [18] This spectrum was used as input to simulate the damage from polyenergetic omnidirectional proton irradiation in the orbit of the ISS, as shown in Figure 1. See supplementary materials for computational details.

In-operando measurements after stopping the proton irradiation

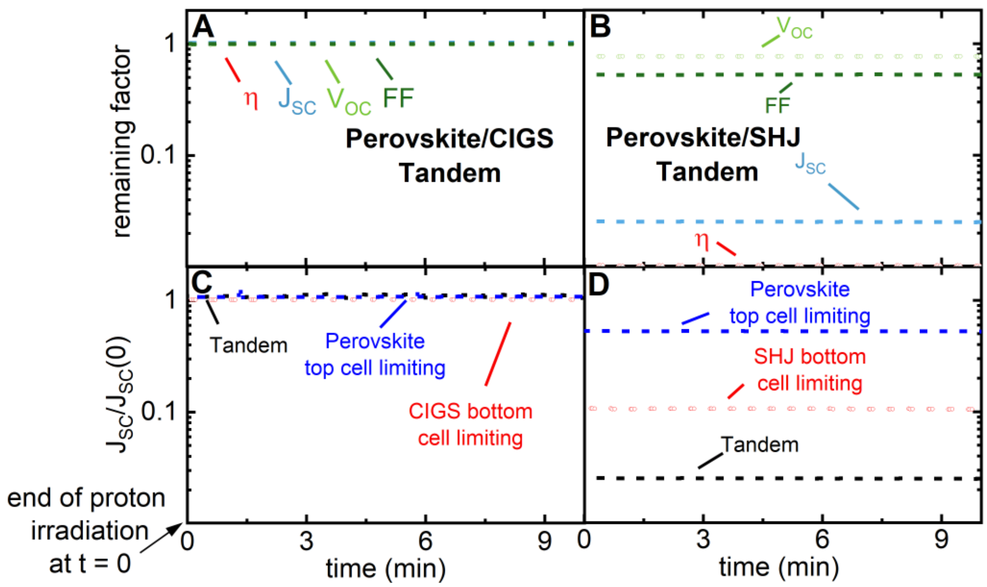

Figure S*2*: (A**,** B) *In-operando* measurements of V_{OC}, J_{SC}, FF, and η normalized to their initial value of the perovskite/CIGS and perovskite/SHJ tandem solar cells, related to Figure 1. Measurements were started directly after stopping the proton irradiation (after $\Phi = 2{\cdot}10^{12}$ p^+/cm^2). (C, D) Normalized short-circuit current of the perovskite/CIGS and perovskite/SHJ tandem solar cell under illumination with NIR and blue LED's at a wavelength of λ = 450 nm/850 nm alternatingly set to either 100 % or 14/5 %, again directly after stopping the proton irradiation. The employed illumination regimes are described in the main manuscript in more detail. (Fig 1G)

Operando Perovskite/SHJ JV characteristics

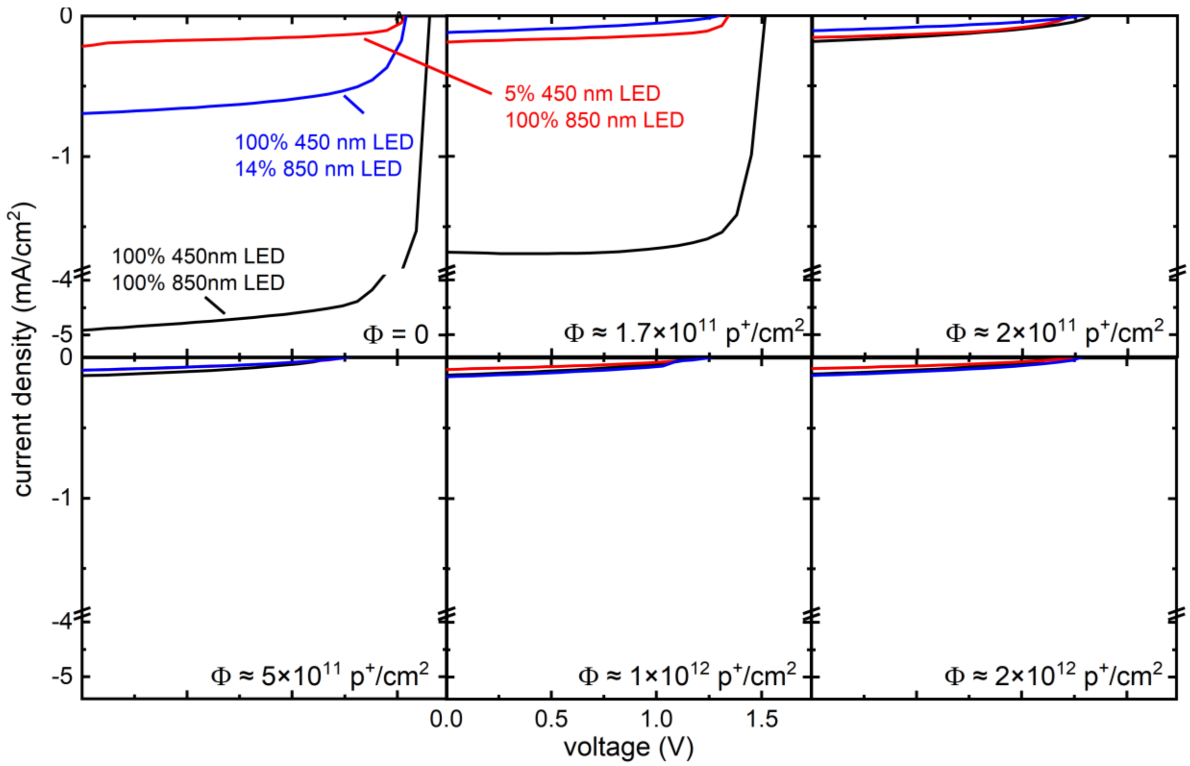

Figure S3: *Operando* JV characteristics under 450 and 850 nm LED illumination set to I^{450nm} = 100% & I^{850nm} = 100% (black), I^{450nm} = 100% & I^{850nm} = 5% (blue), I^{450nm} = 14% & I^{850nm} = 100% (red) of the perovskite/SHJ tandem solar cells under 68 MeV proton irradiation for accumulated doses of Φ ≈ 0, 1.7×10^{11}, 2×10^{11}, 5×10^{11}, 1×10^{12}, and 2×10^{12} p^+/cm^2., related to Figure 1.

Operando Perovskite/CIGS JV characteristics

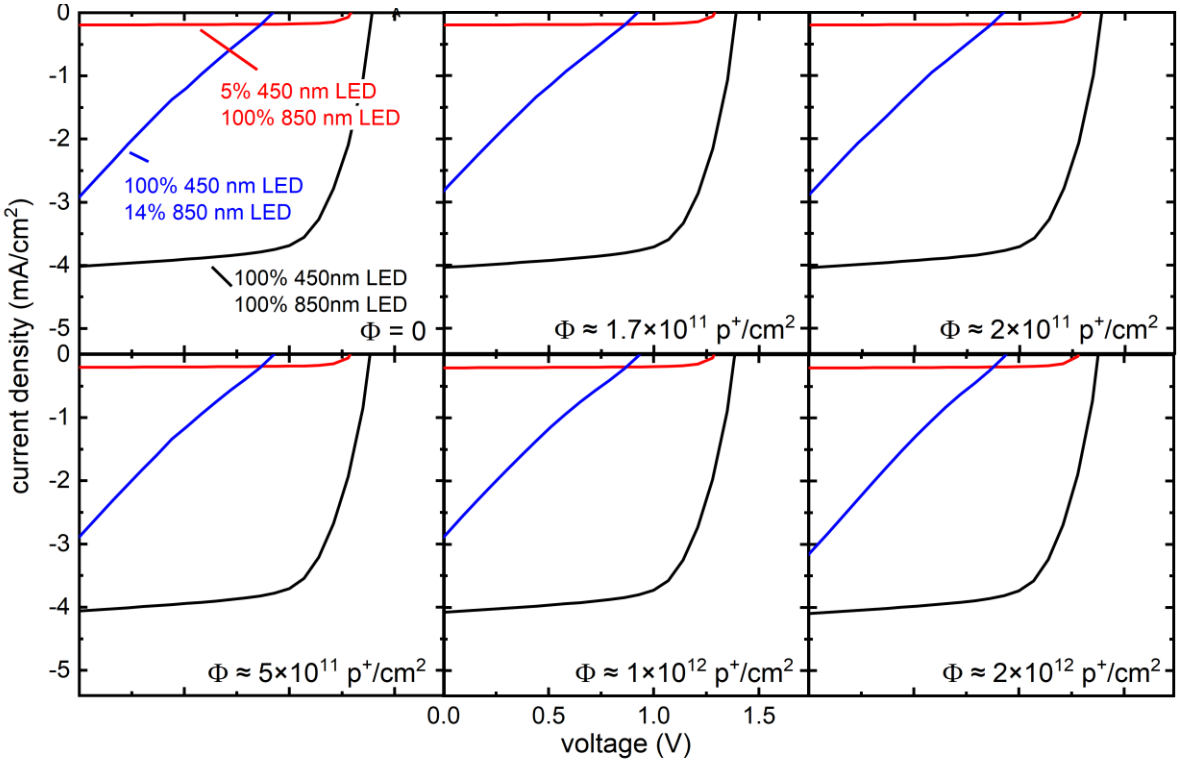

Figure S4: *Operando* JV characteristics under 450 and 850 nm LED illumination set to I^{450nm} = 100% & I^{850nm} = 100% (black), I^{450nm} = 100% & I^{850nm} = 5% (blue), I^{450nm} = 14% & I^{850nm} = 100% (red) of the perovskite/CIGS tandem solar cells under 68 MeV proton irradiation for accumulated doses of Φ ≈ 0, 1.7×10^{11}, 2×10^{11}, 5×10^{11}, 1×10^{12}, and 2×10^{12} p^+/cm^2, related to Figure 1.

Interestingly, the perovskite/SHJ and perovskite/CIGS tandem solar cells behave quite differently when the bottom cell is limiting the overall current (under illumination with 14% 850nm, 100% 450nm). We believe that this is caused by the lower shunt resistance of the CIGS bottom cell compared to the SHJ bottom cell. Evidence for this is found in Figure S10 and S19, where we provide the dark JV characteristics and derive a 10x higher shunt resistance in the case of SHJ bottom cells. A similar situation is often seen in commercial InGaP/GaAs/Ge triple-junction solar cells, where the InGaP and Ge sub-cells exhibit JV characteristics that are similar to the CIGS case here when forced into limitation by appropriate light biasing. Often this then complicates spectral response measurements. [19]

Summarized photovoltaic parameters

Table S1: Summarized photovoltaic parameters. In all cases, JV measurements were recorded from J_{SC}-to-V_{OC} and V_{OC}-to-J_{SC}. Given that the MPP tracking indicates similar results, we summarize parameters extracted from J_{SC}-to-V_{OC} scans only.

		V_{OC} (V)	J_{SC} mA/cm^2	FF %	η %	$η_{MPP}$ %	P_{MPP} W/m^2	J_{SC-EQE}^{top} mA/cm^2	J_{SC-EQE}^{bottom} mA/cm^2
**Perovskite/SHJ**									
as prep	AM1.5G	1.73	18.0	67.9	21.1	21.3	213	21.9	18.0
as prep	AM0	1.76	21.5	66.7	18.8	19.2	257	23.5	21.5
irradiated	AM1.5G	1.40	0.26	50.8	0.18	0.33	3.3	17.9	0.26
irradiated	AM0	1.41	0.3	52.8	0.16	0.31	4.0	22.0*	0.30
								*f = 4Hz	
**SHJ single**									
as prep	AM1.5G	0.74	36.3	77.9	20.9	-	-	36.3	
irradiated	AM1.5G	0.33	1.0	52.7	0.18	-	-	0.35	
**Perovskite/CIGS**									
as prep	AM1.5G	1.52	16.7	70.8	18.0	18.0	180	17.9	16.7
as prep	AM0	1.55	20.0	65.7	15.2	15.1	202	21.9	20.0
irradiated	AM1.5G	1.50	16.8	59.4	14.9	15.77	158	18.3	15.2/16.6*
irradiated	AM0	1.52	19.4	57.4	12.6	12.90	173	22.5	18.1/19.9*
									*f = 144Hz
**CIGS single**									
as prep	AM1.5G	0.64	34.2	70.9	15.5	-	-	34.2	
irradiated	AM1.5G	0.61	34.5	65.4	13.8	-	-	34.3	

Proton Irradiation of Identically Prepared SHJ Single Junction Solar Cells

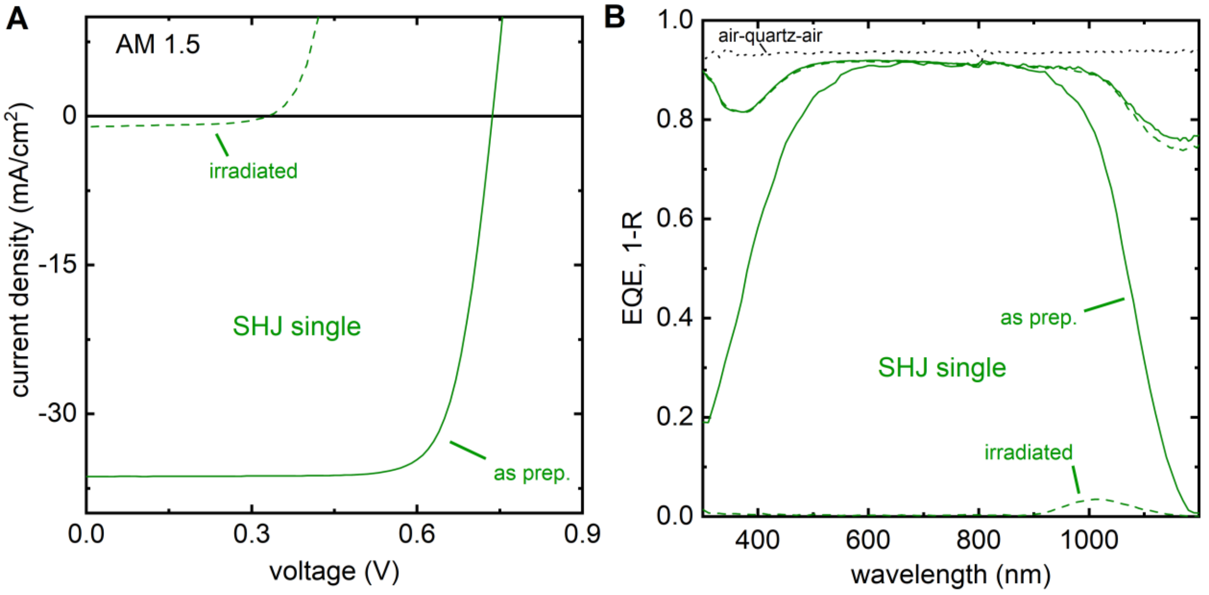

Figure S5: (A) Current-voltage characteristics of SHJ single junction solar cells under AM1.5G illumination conditions. Solid lines refer to measurements performed on as prepared solar cells, while dashed lines refer to measurements after 68 MeV proton irradiation at a total dose of $\Phi = 2 \cdot 10^{12}$ p^+/cm^2. (B) External quantum efficiency of the SHJ single junction solar cells before and after proton irradiation. The reflection is shown as 1-R. Related to Figures 2 and 5.

Perovskite/SHJ tandem solar cells

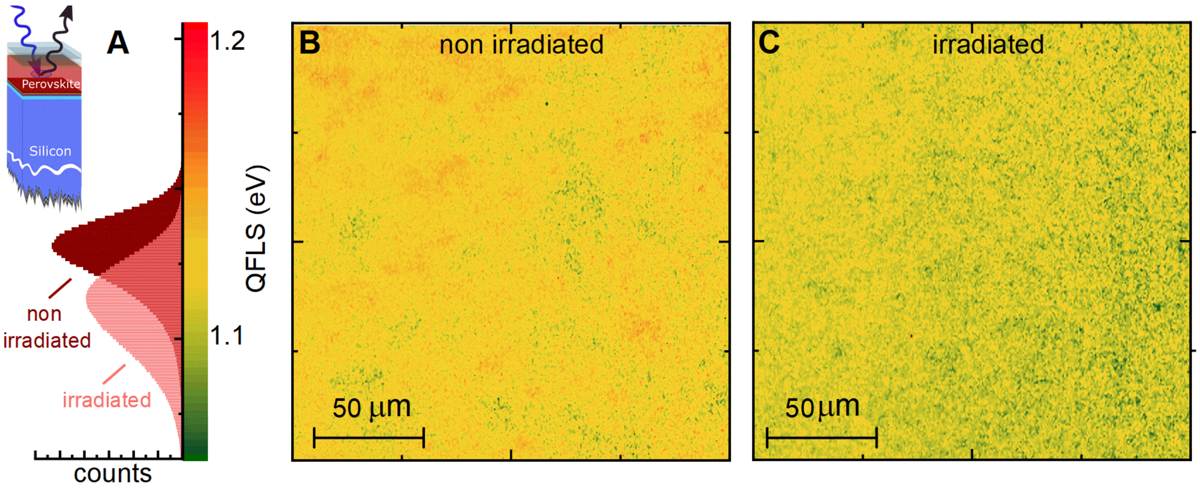

Figure S6: (A) Quasi-Fermi-Level-Splitting (QFLS) histogram and (B, C) QFLS maps of the perovskite top absorber in the as-prepared and proton-irradiated perovskite/CIGS tandem solar cell measured under 405 nm laser illumination with an intensity equivalent to 1 sun (see SM for details). Related to Figure 4.

Absolute photoluminescence imaging with high spatial resolution of the perovskite sub-cell in perovskite/SHJ tandem solar cells

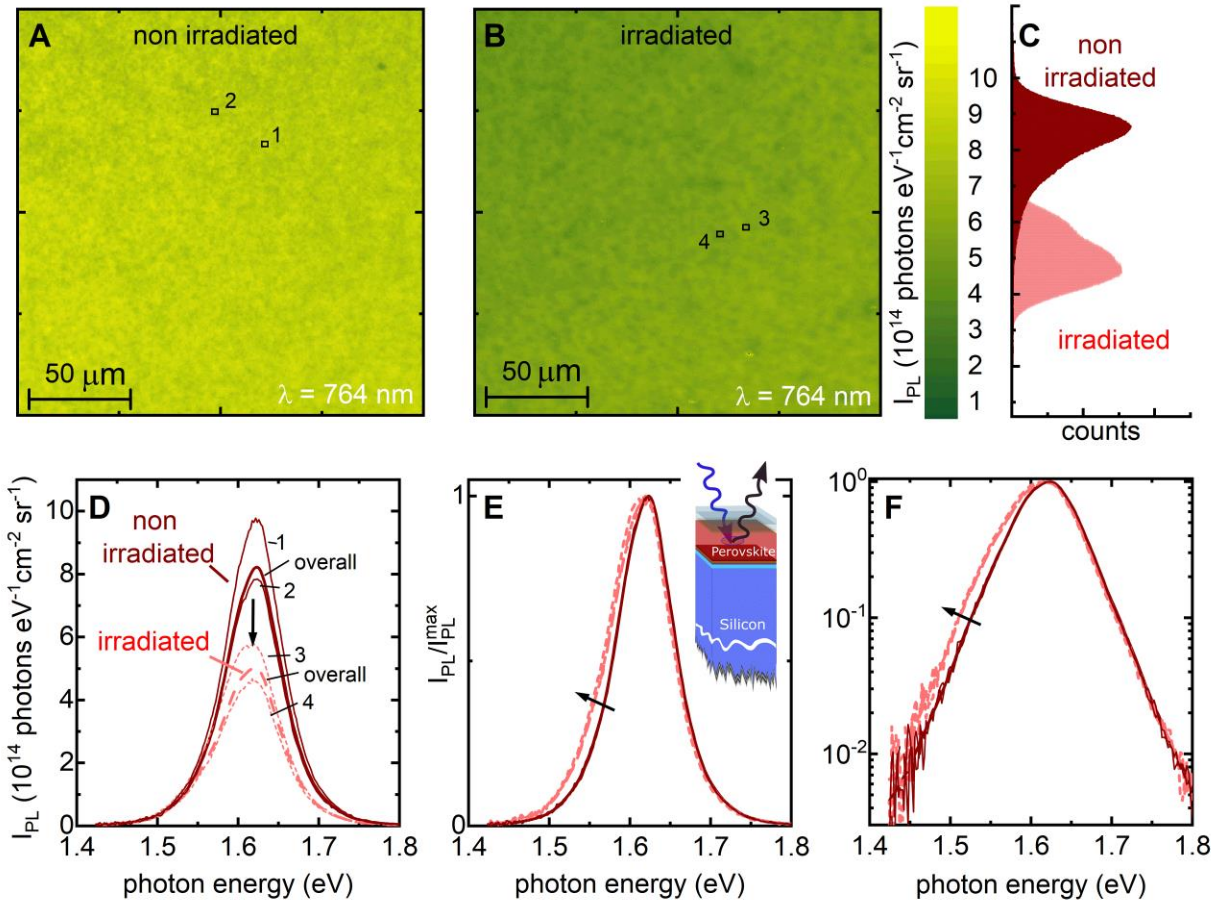

Figure S7: (A, B) Absolute photoluminescence images of the non-irradiated and irradiated (E_p = 68 MeV, $\Phi = 2 \cdot 10^{12}$ p^+/cm^2) perovskite sub-cell in the perovskite/SHJ tandem solar cell at a wavelength of λ = 764 nm. (C) Corresponding photoluminescence histogram. (D) Corresponding photoluminescence spectra. Data obtained on the non-irradiated and irradiated specimens are plotted as solid and dashed lines, respectively. Thick lines refer to the overall photoluminescence spectra, while thin lines refer to bright and dark regions, as indicated in (A, B) As described in the main text, the overall PL intensity reduces by a factor of two after proton irradiation. (E) and (F) depict the same data-set, now normalized, on a linear and semi-logarithmic scale. Evidently, both bright and dark regions feature a red-shifted shoulder after proton irradiation. Related to Figure 3.

Photoluminescence lifetime mapping with high spatial resolution of the perovskite/SHJ tandem solar cells

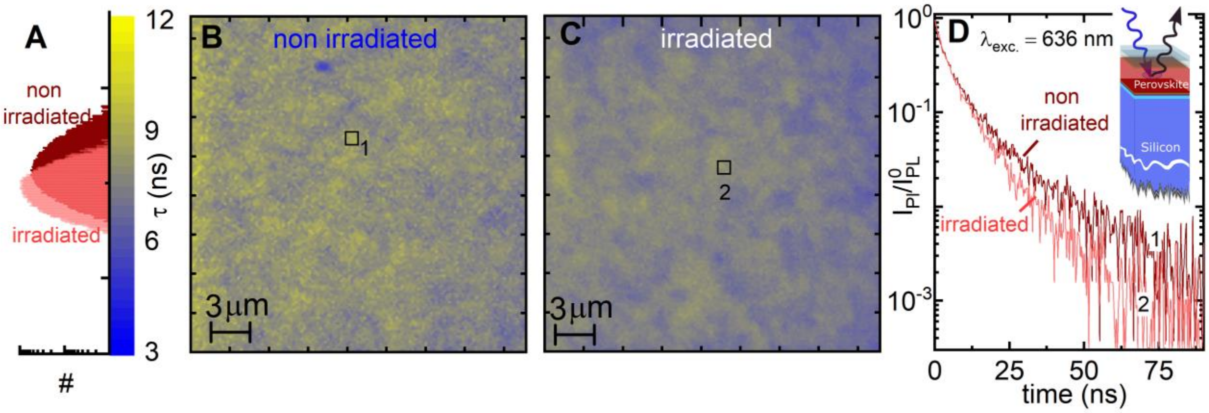

Figure S8: (A, B, C) Photoluminescence lifetime histogram and maps of the non-irradiated and irradiated (E_p = 68 MeV, Φ = 2·10^{12} p^+/cm^2) perovskite/SHJ tandem solar cells under excitation with a wavelength of λ_{exc} = 636 nm. Lifetimes were obtained by fitting the photoluminescence decay on each pixel with a bi-exponential decay. In general, slightly shorter lifetimes are observed after proton irradiation. Moreover, heterogeneity increases similar to the trend derived from absolute PL maps shown in Fig S3. (D) Selected TRPL decays of areas indicated in B and C. All measurements were performed on the complete device stack. Selective contacts are known to quench the PL decay in hybrid perovskites significantly, due to rapid charge carrier extraction into the contact layers and/or additional surface recombination at the interface between perovskite and the employed selective contacts. [20] Related to Figure 3.

Optical spectroscopy of the SHJ bottom cell

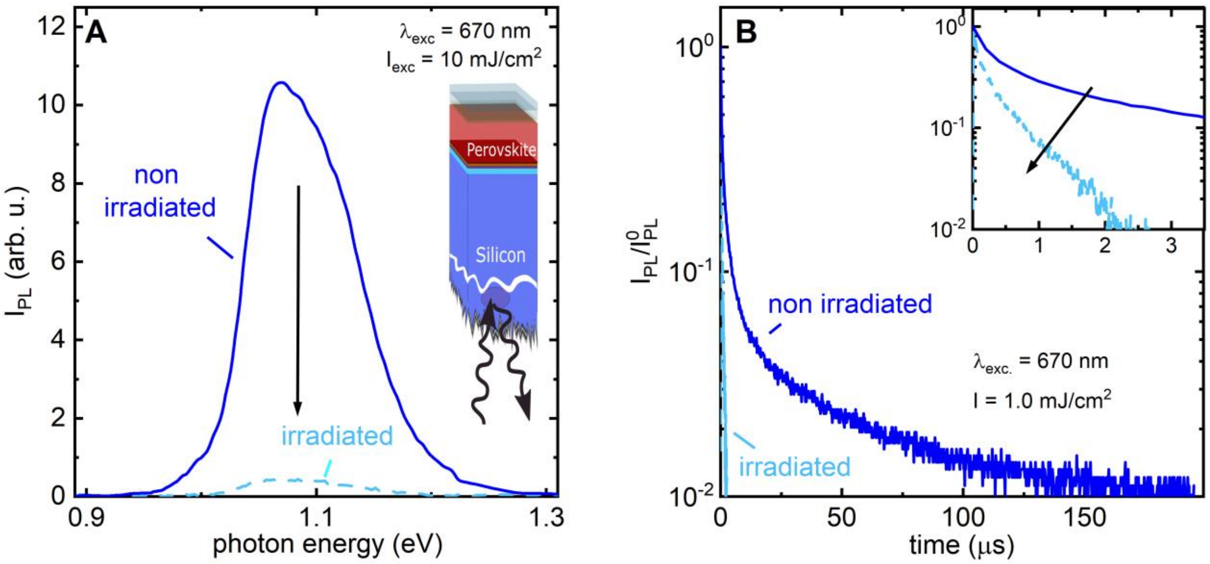

Figure S9: Photoluminescence spectra (A) and transient photoluminescence (TRPL) (B) of the SHJ bottom absorber before and after proton irradiation. In both cases, excitation was performed from the backside on regions that were not covered with Ag. Related to Figure 3.

Dark current-voltage characteristics of the perovskite/SHJ tandem solar cells

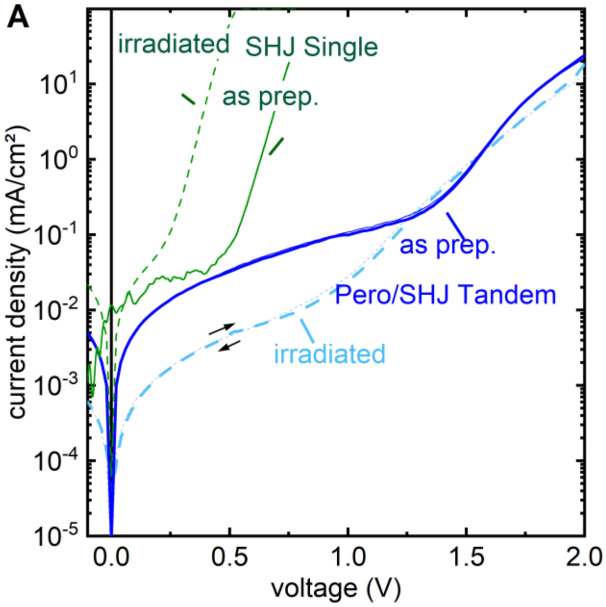

Figure S10: (A) Dark current-voltage and (B) differential-resistance-voltage characteristics of investigated Perovskite/SHJ Tandem and SHJ Single junction solar cells. Solid and dashed lines refer to as prepared and irradiated specimens, respectively. (E_p = 68 MeV, $\Phi = 2 \cdot 10^{12}$ p^+/cm^2) Interestingly, we observe an increase in rectification and differential resistance for the perovskite/SHJ tandem solar cells. Similar increases have been found for perovskite single junction solar cells.[21] SHJ single junction solar cells feature a substantial decrease of the built-in-voltage after irradiation that corroborates the V_{OC} losses after irradiation. Related to Figure 2.

Supplementary Note 1

To further assess the impact of the proton irradiation on the perovskite sub-cell, we selectively probed the perovskite top cell using steady-state and time-resolved photoluminescence (PL and TRPL) measurements. Similar to the perovskite/CIGS tandem solar cells, we employ absolute hyperspectral PL imaging with high spatial resolution to record the local QFLS of the perovskite sub-cell with 405 nm excitation generating equivalent current densities to 1 sun. In Figure S3, we show QFLS maps of an irradiated (E) and a non-irradiated device (D), along with the number histograms from the measured pixels (C). The mean QFLS remains high and reduces only slightly from 1.13 ± 0.01 eV (non-irradiated) to 1.11 ± 0.02 eV (irradiated). Similar to the perovskite/CIGS case, an increase in heterogeneity can be identified, as seen in the slightly negatively skewed histogram. Interestingly, we observe a low energy shoulder in PL emission throughout the entire specimen after irradiation, which indicates the presence of radiation-induced tail states. (Figure S7 E & F)) Concomitant TRPL maps reveal an increase in heterogeneity as well as an overall decreased lifetime (Figure S8), consistent with the generation of radiation-induced tail states. Note that we do not observe similar changes of the perovskite PL when deposited on CIGS (Figure S14 E), which we attribute to radiation-induced changes of NiO that increases and prolongs the PL and TRPL of the perovskite sub-cell in the perovskite/CIGS tandem stack. (see the main manuscript for further details). To compare these subtle changes in perovskite sub-cell luminescence to the SHJ bottom-cell, we selectively probed the SHJ PL and TRPL in the tandem stack by excitation from the bottom. PL and TRPL data plotted in Figure S9B & C reveal large differences. Most importantly, the PL intensity is quenched by an order of magnitude after proton irradiation. Furthermore, the PL lifetime is drastically reduced after irradiation. Both effects indicate the presence of radiation-induced recombination centers in the SHJ bottom cell. This is corroborated by identically prepared and irradiated SHJ single junction devices, which show a vast reduction in J_{SC} and EQE from 36.3 mA/cm^2 and 92 % (max) to 1 mA/cm^2 and 3.4 % (max) after proton irradiation. (Note that the change in J_{SC} of 36.3 to 0.34 mA/cm^2 and 19.9 to 0.26 mA/cm^2 is larger for the SHJ single junction device compared to the SHJ bottom cell, resulting in a larger radiation-induced V_{OC} loss in SHJ single junctions compared to SHJ bottom cells)

Radiation-induced Si-vacancies (V_{Si}) and di-vacancies are known to diminish the minority carrier lifetime of crystalline silicon [22–28], and the associated gradual decrease in performance during proton irradiation has been studied extensively. [26,27,29–33] Abrupt degradation of the short-circuit current and performance of silicon solar cells during high-energy high-fluence proton irradiation has further been observed and explained by an exponential decrease in majority carrier concentration, which provokes an abrupt increase in base resistance. [23] The perovskite/SHJ tandem solar cell tested here relies - like the majority of perovskite/SHJ tandem solar cells to date [1,34–36] - on a phosphorous doped n-type float zone (FZ) Si base. Positron annihilation spectroscopy has recently shown that high energetic proton irradiation of n-type silicon forms thermally stable phosphorous-divacancy complexes that are majority carrier traps. [22]

This can de-activate the phosphorous doping and reduce the J_{SC} reduces rapidly. While dissociation of Si di-vacancies and phosphorous-divacancy complexes can restore the minority carrier lifetime and the majority carrier concentration, the required temperatures of 450 K and above would inevitably deteriorate the perovskite sub-cell with its organic contact layers. [23,25] Optimized Si space solar cells, often rely on a boron-doped p-type base and a very thin wafer thickness of 50 μm to withstand about 10^{14} p/cm^2 at 10 MeV. [23] Radiation- and light-induced boron-oxygen complexes, however, are efficient minority carrier traps and, hence, it will be crucial to use float zone silicon wafers with low oxygen content. [37,38] Using a p-type FZ silicon base for perovskite/SHJ tandem solar cells is not straight forward as the required regular perovskite top cell, often relies on high-temperature TiO_2 contact layers which would deteriorate employed hydrogenated amorphous silicon layers. [39] Further, inverted perovskite top cells, as used in this study, are often found to be more reliable and robust against UV, oxygen, and moisture-induced degradation. [40] Using a thin silicon base, ultimately, will require advanced light management strategies to ensure the efficient collection of NIR light. [41]

The most promising measures to strengthen the radiation hardness of perovskite/SHJ tandem solar cells, therefore, comprise an increased perovskite bandgap (forcing the tandem into limitation by the more radiation hard perovskite, thereby masking radiation-induced degradation of the SHJ bottom cell) and/or changes of the SHJ architecture (to become more robust to the inevitable radiation-induced lifetime loss, i.e., by employing a thinner silicon base). Both measures will, however, sacrifice some of the excellent initial performance for improved radiation hardness. Further, the vast and inherent impact of radiation-induced defects on the electrical properties of crystalline silicon makes such approaches challenging and ultimately more expensive.

Proton Irradiation of Identically Prepared CIGS Single Junctions Solar Cells

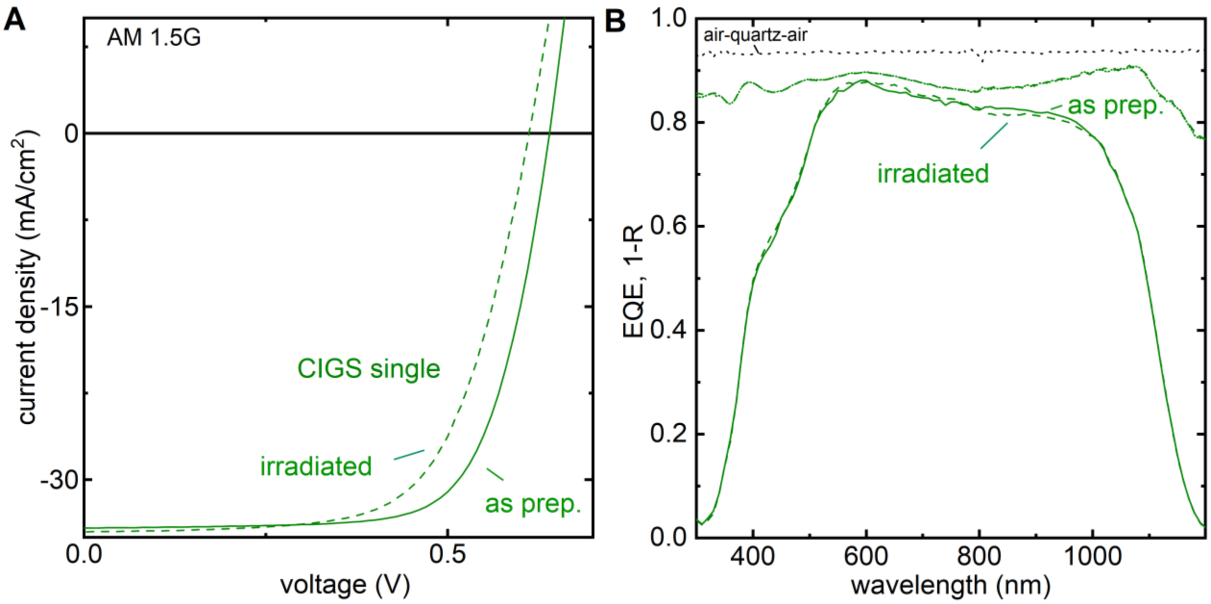

Figure S11: (A) Current-voltage characteristics of CIGS single junction solar cells under AM1.5G illumination conditions. Solid lines refer to measurements performed on as prepared solar cells, while dashed lines refer to measurements after 68 MeV proton irradiation at a total dose of $\Phi = 2 \cdot 10^{12}$ p^+/cm^2. (B) External quantum efficiency of the CIGS single junction solar cells before and after proton irradiation. The reflection is shown as 1-R. Related to Figure 2.

Dark current-voltage characteristics for perovskite/CIGS tandem solar cells

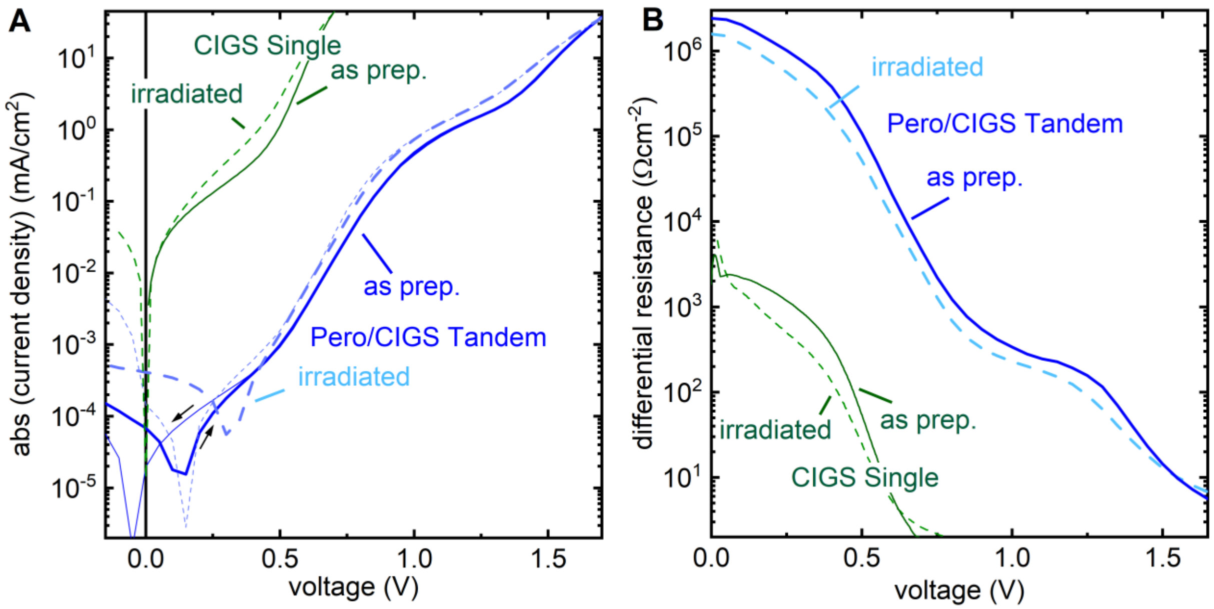

Figure S12: (A) Dark current-voltage and (B) differential resistance-voltage characteristics of investigated perovskite/CIGS tandem and CIGS single junction solar cells. Solid and dashed lines refer to as prepared and irradiated specimens, respectively. (E_p = 68 MeV, $\Phi = 2{\cdot}10^{12}$ p^+/cm^2) In contrast to the perovskite/SHJ tandem solar cell no increase in rectification and differential resistance visible. Interestingly, the perovskite/CIGS tandem solar cell features some hysteresis, which is visible only in the dark JV curves and not under AM0 or AM1.5G illumination. Related to Figure 2.

Photoluminescence lifetime mapping with high spatial resolution of the perovskite sub-cell in the perovskite/CIGS tandem solar cell

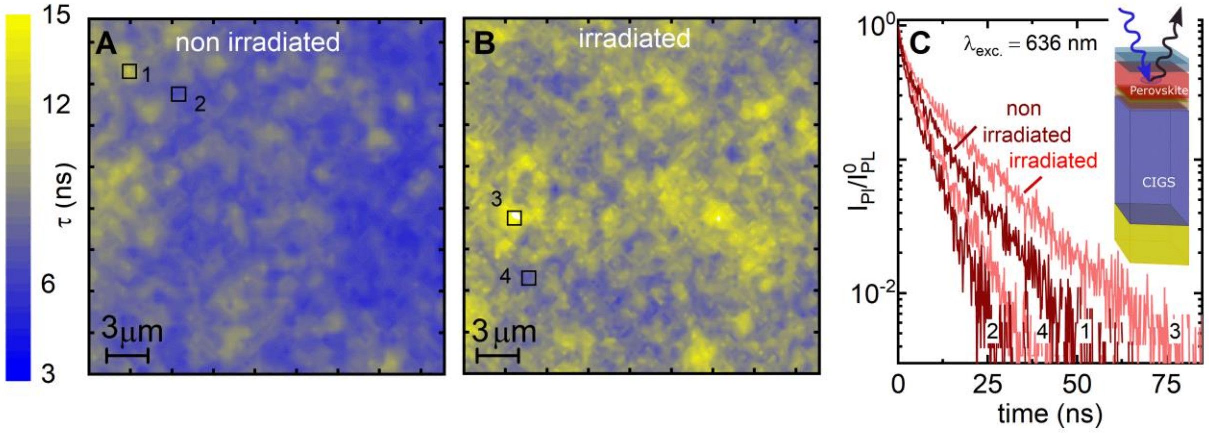

Figure S13: (A, B) Photoluminescence lifetime mapping of the non-irradiated and irradiated (E_p = 68 MeV, $\Phi = 2 \cdot 10^{12}$ p^+/cm^2) perovskite sub-cell in the perovskite/CIGS tandem solar cell under excitation with a wavelength of $\lambda_{ex.}$ = 636 nm, based on a single exponential fitting of the photoluminescence decay on each pixel. (C) Photoluminescence decay of selected short and long lifetime regions on the non-irradiated and irradiated specimens. The PL lifetime maps show an increased heterogeneity and more long-lifetime areas after irradiation. Overall this results in a longer lifetime. Related to Figure 4.

Absolute photoluminescence imaging with high spatial resolution of the perovskite sub-cell in the perovskite/CIGS tandem solar cell

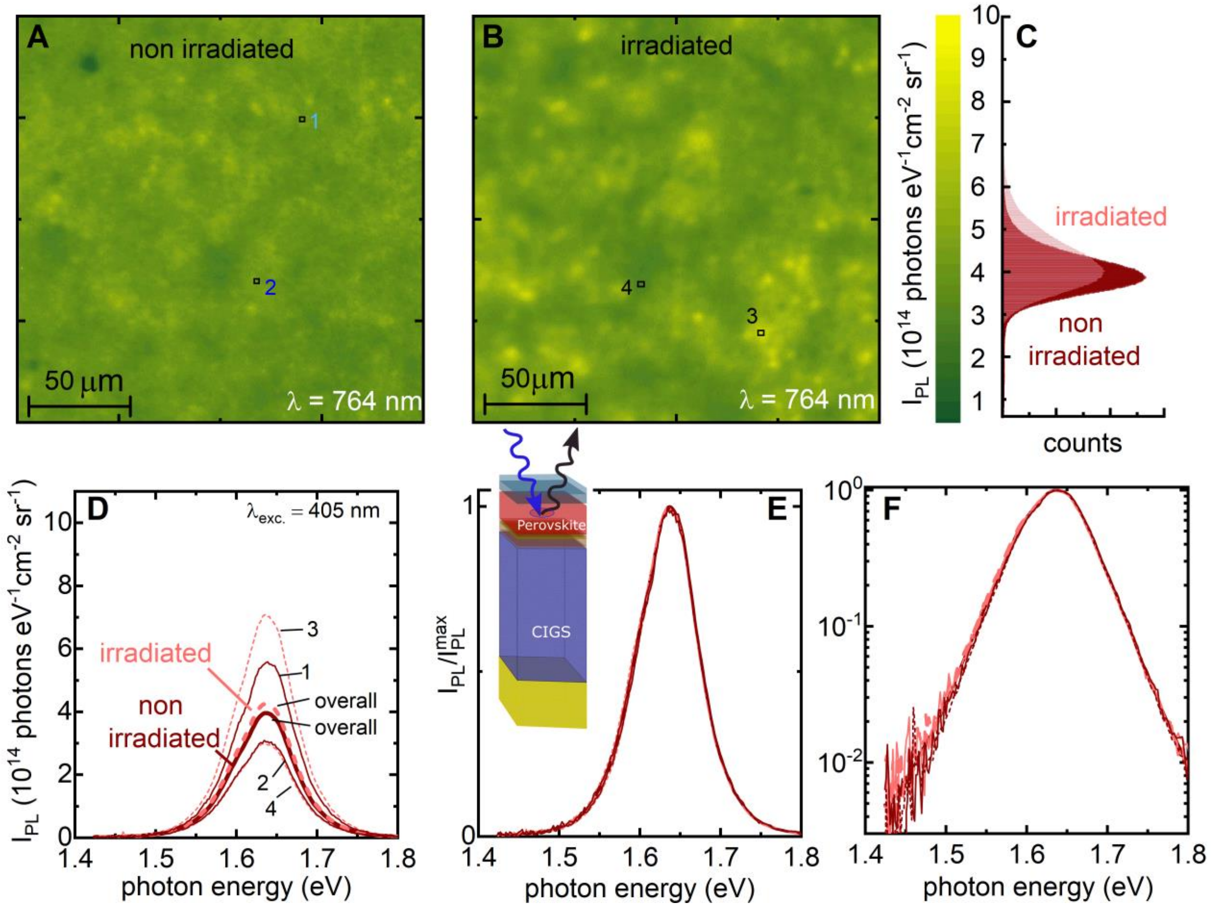

Figure S14: (A, B) Absolute photoluminescence images of non-irradiated and irradiated (E_p = 68 MeV, $\Phi = 2{\cdot}10^{12}$ p^+/cm^2) perovskite sub-cell in the perovskite/SHJ tandem solar cell at a wavelength of λ = 764 nm. **c** Corresponding photoluminescence histograms revealing an increase in heterogeneity. (D) Corresponding photoluminescence spectra. Data obtained on the non-irradiated and irradiated specimens are plotted as solid and dashed lines, respectively. Thick lines refer to the overall photoluminescence spectra while thin lines refer to bright and dark regions, as indicated in A Interestingly, bright regions feature a higher PL intensity after irradiation, while the PL intensity of dark regions remains unchanged. (E) and (F) depict the same data-set, now normalized and on a linear and semi-logarithmic scale. In contrast to the PL spectra of the perovskite/SHJ tandem, no low energy shoulder is observed. Related to Figure 4.

Spectral mismatch of mimicked AM0 and AM1.5G conditions

To estimate the spectral mismatch of the mimicked AM0 and AM1.5G spectra, we measured the irradiance as a function of wavelength and compare it in figure S11 to the ASTM G-173 and ASTM E-490 spectra. The upper panel further specifies the spectral mismatch in 100 nm intervals, calculated from the ratio of the incident irradiance integrated over the specified wavelength interval.

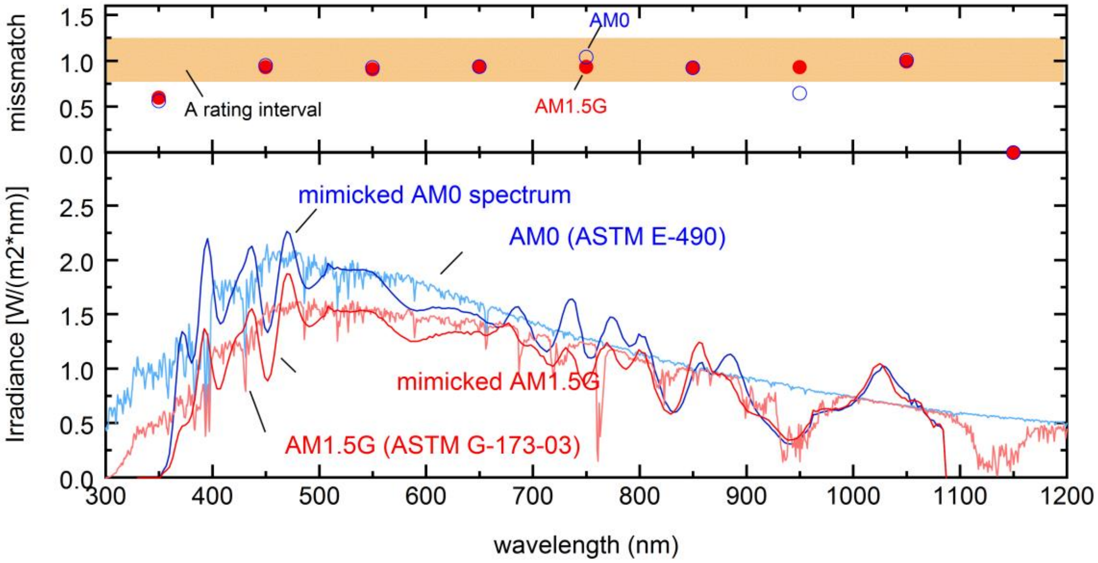

Figure S15: Mimicked AM 0 and AM 1.5G spectra, compared to the standard AM0 and AM1.5G spectra[42]. The top panel depicts the spectral mismatch in 100 nm intervals. Related to Figure 2.

Supplemental Experimental Procedures

Preparation of Perovskite/SHJ Tandem Solar Cells*:* Prepared perovskite/SHJ tandem solar cells comprise LiF/IZO/SnO_2/C_{60}/Perovskite/PTAA/ITO/(n^+) nc-SiOx/(i)a-Si:H/(n)-c-Si/(i)-a-Si:H/(p)-a-Si:H/Al:ZnO/Ag and feature an active area of 0.81 cm^2. A detailed description of the fabrication procedures is described elsewhere. [1] In here, we detail fabrication of the two absorber layers only: For the perovskite top absorber 1.5 M nominal PbI_2 and $PbBr_2$ in DMF:DMSO = 4:1 volume were first prepared as stock solutions and then added to FAI and MABr with 10 % PbX_2 excess, respectively (X = I or Br). The so obtained $FAPbI_3$ and $MAPbBr_3$ were then mixed in a 5:1 volume ratio to obtain a "double cation" perovskite. Finally, 5 % volume of 1.5 M nominal CsI in DMSO was added to form a "triple cation" perovskite and subsequently spun at 4000 rpm for 35 s. 25 s after the start of a spinning, 400 µl ethyl acetate anti-solvent drop was utilized, and finally, the films were annealed at 100°C for 1 h. For the SHJ bottom cell, a 260 µm thick polished FZ <100> n-type crystalline silicon (c-Si) wafer in a rear junction configuration was used. The front surface of the wafer was left polished to facilitate the perovskite top cell deposition, while the rear surface of the wafer was chemically textured to obtain random pyramids with <111> facets to improve the optical response of the bottom cell in the NIR region. Again, a more detailed description can be found elsewhere. [1] Fabricated Perovskite/SHJ tandem solar cells were then encapsulated under inert atmosphere using a quartz cover and epoxy resin. No intermediate index matching layers or light-management foils were used, as they typically darken upon proton irradiation due to the formation of radiation-induced color centers. As we found previously, such coloring can reduce the J_{SC} of perovskite solar cells dramatically and consequently dominate proton irradiation-induced degradation. [2]

Preparation of Perovskite/CIGS Tandem Solar Cells*:* Prepared perovskite/CIGS tandem solar cells comprise LiF/IZO/SnO_2/C_{60}/Perovskite/PTAA/NiO_x/ZnO/CdS/CIGS/Mo/glass and feature an active area of 0.81 cm^2. A detailed description of the involved preparation procedures is given elsewhere. [3] Here, we summarize the deposition of both absorber layers and the NiOx layer only: The perovskite absorber is prepared identically to the perovskite/SHJ tandem solar cells described above. For the CIGSe absorber, thermal evaporation from elemental sources using a processing sequence based on the well-known multi-stage process is used. [4] In this process, an $(In,Ga)_2Se_3$ precursor is deposited at a substrate temperature of 300 °C in the first stage, followed by the evaporation of Cu and Se at a substrate temperature of 530 °C until the absorber becomes overall copper-rich. The process is completed by the evaporation of In, Ga, and Se in the last stage to make the absorber copper poor again (CGI=0.9, GGI=0,3). The NiOx was deposited after a pre-deposition oxygen plasma treatment using atomic layer deposition from bismethylcyclopentadienyl-nickel ($Ni(MeCp)_2$), 97%, Sigma-Aldrich) as the precursor and an O_2 plasma as the co-reactant. A total of 350 ALD cycles were performed each consisting of 3 s $Ni(MeCp)_2$ dose, 4 s purge time , 3 s O_2 plasma exposure, and 1 s purge time. Details on the NiOx process development including the saturation curves are published elsewhere. [5] Similar to perovskite/SHJ tandem cell, the fabricated perovskite/CIGS tandem solar cells were then encapsulated under inert atmosphere using a quartz cover and epoxy resin.

Proton Irradiation*:* The tandetron-cyclotron combination of the Helmholtz-Zentrum Berlin[6,7] was used to accelerate protons to an energy of 68 ± 1 MeV. A thin scattering foil and appropriate aperture masks were used to provide a homogeneous irradiation over an area of 3.0 cm^2. The beam intensity was monitored online, utilizing a transmission ionization chamber. After irradiation, all devices were transferred to a lead cabinet until the activity of generated short-living isotopes in the irradiated samples dropped to a safe level of less than 10^3 Bq (~10 days of storage). To avoid the thermally-activated healing of radiation-induced defect states often observed in CIGS absorbers above 400 K, the devices were stored at RT in the dark. [8–10]

***In-operando* measurements**: *In-operando* measurements of the photovoltaic parameters were recorded during proton irradiation. Therefore, a Keithley 2400 was remotely controlled to record JV measurements from V_{OC}-to-J_{SC} every 3 seconds. This high repetition rate did not allow for reverse and forward measurements. Illumination was performed using two high-intensity LED's that are selectively absorbed in the high and low gap sub-cells (λ_{LED}^{1} = 450 nm and λ_{LED}^{2} = 850 nm). When set to 100%, both LED illuminated the entire active area homogenously at about ¼ AM0. Then the λ_{LED}^{1} = 450 nm LED was set to 0.7 mW/cm^2 (5 % of its initial value), thereby forcing the perovskite sub-cell to limit the overall current. Subsequently, the λ_{LED}^{2} = 850 nm LED was set to 1 mW/cm^2 (14% of its initial value), thereby forcing the SHJ or CIGS bottom cell to limit the overall current. The three used regimes were alternated every 10 s during proton irradiation using a homemade Arduino controlled circuit. Note, the low-intensity settings were adjusted to slightly different values to allow an unambiguous assignment of the employed regimes. Radiation-induced degradation of the employed LEDs and LED optics was avoided using focusing mirrors and keeping the LEDs at a safe distance from the proton beam.

Current-Voltage-Characteristics: AM1.5G and AM0 spectra were simulated using a Wavelabs Sinus 70 AAA LED sun simulator. The simulated and target spectra, as well as a mismatch calculation, are shown in Fig S11. The intensity was adjusted to 100 or 135 mW/cm² respectively by measuring the short-circuit current of a calibrated silicon solar cell (Fraunhofer ISE). Current-voltage scans were performed in forward and reverse direction with a voltage sweep of 85 mV/s. Additionally, we tracked the maximum power point (MPP) using homemade feedback software. In all cases, the temperature amounted to 25°C. In some cases, shadow masks (A = 0.778 cm^2) were used to avoid underestimation of the active area.

Suns-V_{OC} measurements: To estimate ideality factors, we varied the incident light intensity by modulating the LED intensity and measured the stabilized V_{OC}. In all cases, the temperature was set to 25°C using a temperature-controlled stage. In the case of single junction solar cells, the slope of the Suns-V_{OC} data can be used to estimate the ideality factor n using:

$$V_{OC,X} = \frac{k_B T}{q} \cdot n \cdot \ln(X) + V_{OC,X=1} \quad (1)$$

Here k_B is the Boltzmann constant, and T is the temperature. In the case of monolithic tandem solar cells, the V_{OC} equals the sum of the sub-cell open-circuit voltage $V_{OC,i}$. and hence, the intensity dependence of the V_{OC} can be approximated by:

$$V_{OC,X}^{tandem} = \frac{k_B T}{q} \cdot \sum_i n_i \cdot \ln(X) + V_{OC,X=1}^{tandem} \quad (2)$$

Here the n_i denotes the ideality factor of the individual sub-cell i. The ideality factor of a tandem solar cell n^{tandem} is thus given by the sum of the individual sub-cell ideality factors.

$$n^{tandem} = \sum_i n_i \quad (3)$$

An identical finding is obtained using the segmental approximation often employed to describe the current-voltage characteristics of multijunction solar cells, including InGaAs/GaAs and GaInP/GaInAs/Ge tandem solar cells. [11]

External quantum efficiencies (EQE): Appropriate light and voltage biases are required to record the spectral response of the perovskite/SHJ and perovskite/CIGS tandem cells. For this purpose, we employed LEDs at a wavelength of λ = 455 nm and λ = 855 nm that are selectively absorbed in the respective sub-cells. In case of the perovskite/SHJ tandem, the perovskite sub-cell was measured at an intensity of 2.3 mW/cm^2 (λ = 455 nm) and 2.9 mW/cm^2 (λ = 855 nm)) while the SHJ sub-cell was

measured using 14.5 mW/cm^2 (λ = 455 nm). In the case of the perovskite/CIGS tandem, an intensity of 6 mW/cm^2 (λ = 855 nm) and 16.4 mW/cm^2 (λ = 455 nm) was used for the perovskite and CIGS sub-cells, respectively. The CIGS bottom cell was also measured under bias light from a halogen lamp (109 mW/cm^2, 1.1sun (AM15) equivalent) equipped with a 750nm short pass filter. The EQE of both sub-cells was integrated over an AM1.5G or AM0 spectrum. Derived values were used to avoid overestimation of the J_{SC} measured under mimicked AM1.5G and AM0 illumination. Standardly, the monochromatic probe was chopped at 74 Hz. In the case of the perovskite/SHJ tandem solar cell, we additionally employed lower chopper frequencies as indicated. In the case of the perovskite/CIGS tandem solar cell, we further used higher chopper frequencies as indicated.

Absolute Photoluminescence imaging: Absolute photoluminescence maps were recorded using a hyperspectral widefield imager from Photon etc. at a magnification of 20x. Following literature [12], the setup was calibrated by combining a spectral calibration employing a calibrated halogen lamp that was coupled into an integrating sphere and an absolute calibration at one wavelength using a fiber-coupled laser. Excitation was performed using a 405 nm laser set to 1 sun (AM1.5G) equivalent fluence. The laser intensity was, therefore, adjusted to 2130 W/m^2, which corresponds to $4.3\cdot10^{21}$ photons m^{-2}s^{-1}. Taking into account the EQE at λ = 405 nm of 0.67 and 0.55 for the perovskite/Si and perovskite/CIGS tandem solar cells, respectively $n^{Pero/Si} = 2.92\cdot10^{21}$ m^{-2}s^{-1} and $n^{Pero/Si} = 2.37\cdot10^{21}$ m^{-2}s^{-1} charge carriers are generated. These values correspond well to the generation rate under AM1.5G of $n = \int_{300\,nm}^{800\,nm} AM1.5G(\lambda)d\lambda = 2.1\cdot10^{21}\,m^{-2}s^{-1}$. (Total number of photons in AM1.5 = $3.89\cdot10^{21}$ photons·m^{-2}·s^{-1}) To calculate the QFLS we assumed Lambertian emission and utilize Würfels generalized Planck law [13], which relates the spontaneous emission of photons in a direct semiconductor to the chemical potential of the non-equilibrium charge carrier concentration to the local temperature T providing that the specific absorptivity $\alpha(E)$ is known.

$$I_{PL}(E) = \frac{2\pi E^2 a(E)}{h^3 c^2}\cdot\frac{1}{\exp\left(\frac{E-QFLS}{k_B T}-1\right)} \quad (4)$$

Assuming that the spectral absorptivity approaches unity for photon energies above the bandgap this equation can be simplified to

$$\ln\left(\frac{I_{PL}(E)h^3c^2}{2\pi E^2}\right) = -\frac{E}{k_B T} + \frac{\mathrm{QFLS}}{k_B T} \quad (5)$$

Here I_{PL} is the measured absolute photoluminescence, E the photon energy, k_B the Boltzmann constant, T the temperature, c the speed of light, h the Planck constant. By fitting equation (5) to the high-energy slope of the PL emission, the QFLS and the local charge carrier temperature can be extracted. [14]

For the Suns-QFLS dependence, absolute photoluminescence maps were recorded while varying the excitation intensity between 1 sun and 0.08 sun using a hyperspectral widefield imager from Photon etc. at a magnification of 20x as described above. All maps were averaged to increase the signal-to-noise ratio, and the resulting spectra were fitted to equation (5) to extract the QFLS. In all cases, the local temperature of the probed emissive volume was found to be slightly elevated, and we thus assumed a k_BT of 0.029 eV for the calculation of the internal ideality factor.

Photoluminescence Lifetime Imaging: A confocal single-photon counting fluorescence microscope from Picoquant was used to record photoluminescence lifetime maps of the perovskite top cell. Excitation was performed at 636 nm using a 100x long working distance air objective (NA = 0.8), a repetition rate of 5 MHz, and an intensity of 380 nJ/cm^2/pulse. The photoluminescence was collected through a dichroic mirror, a 640 nm long-pass filter, and a 50 µm pinhole onto a single photon counting SPAD detector. Both the excitation and emission were raster-scanned using a galvo mirror system, where both the objective and sample remain at a fixed position.

(Macro) Photoluminescence Measurements*:* Photoluminescence spectra and transients of the SHJ bottom cell were recorded using a prism monochromator equipped with an InGaAs detector after excitation from a pulsed dye laser with a wavelength of λ_{exc} = 505 nm or 365 nm. The laser fluence amounted to 6 μJ/cm^2 for transient measurements and to 12 mJ/cm^2 in the case of spectral dependent measurements. A continuous-wave temperature controlled 910 nm laser (Thorlabs) was used to photo excite the CIGS bottom cell through the perovskite top cell for spectral dependent measurements. The emission was recorded using an Andor IDus DU490A InGaAs detector. The spot size was recorded using a Thorlabs beam profiler, where the size was set to be to where the intensity of the beam falls to 1/e^2. The laser fluence amounted to 6.1 W/cm^2.

(Macro) Transient Photoluminescence: Transient measurements of the CIGS sub-cell in the perovskite/CIGS tandem were recorded using a confocal single-photon counting microscope from Picoquant equipped with a 10x air objective. Excitation was performed using a pulsed 636 nm laser at a repetition rate of 20 MHz and a fluence of 160 mJ/cm^2. According to the spectral response measurements shown in Figure 2, 8 % of incident 636 nm light is absorbed in the CIGS bottom-cell. The effective excitation fluence of the CIGS absorber thus amounts to 13 mJ/cm^2. Photoluminescence of the CIGS absorber was collected through a dichroic mirror and two consecutive long-pass edge filters (cut-on wavelength of 950 nm and 1100 nm) onto a single photon counting SPAD detector. This allowed an effective suppression of the emission from the perovskite top-cell (maximum at λ = 765 nm).

SRIM Simulations: To estimate the non-elastic damage of monodirectional 68 MeV proton irradiation, a total of $5 \cdot 10^7$ protons were simulated using SRIM [15]. Employed densities were: ρ_{LiF} =2.63 g/cm^3, ρ_{IZO} =6.64 g/cm^3, ρ_{SnO2} =6.85 g/cm^3, ρ_{C60} =1.65 g/cm^3, ρ_{Pero} =4.59 g/cm^3, ρ_{PTAA} =1.2g/cm^3, ρ_{NiOX}=6.67 g/cm^3, ρ_{ZnO}=5.1 g/cm^3 ρ_{CdS}=4.82 g/cm^3, ρ_{CIGS}=5.7 g/cm^3, ρ_{Mo}=10.28 g/cm^3, $\rho_{nc\text{-}SiOX}$=2.3 g/cm^3, $\rho_{a\text{-}Si}$=2.285 g/cm^3, ρ_{Si}=2.285 g/cm^3, ρ_{ZnOAl}=5.1 g/cm^3, ρ_{quartz} =1.72 g/cm^3, ρ_{LiF} =2.63 g/cm^3, ρ_{ITO} =7.2 g/cm^3, and ρ_{Ag} =10.49 g/cm^3. To approximate the damage induced by omnidirectional, poly-energetic irradiation, the proton spectrum at an ISS orbit (see Fig S1) was used. Using Python[16] and PYSRIM[17] automated SRIM simulations were performed varying the incident proton energy as well as the incident angle between 0 and 90° in 10°steps. In case of the perovskite/CIGS tandem solar cell, the depth-averaged energy loss of monoenergetic 68 MeV proton irradiation amounted to 6.3×10^{-5} eV/(Å ion), while for the ISS orbit an average of 4.34×10^{-5} eV/(Å ion) is obtained. With a total flux of about 1×10^3 p^+ cm^{-2} s^{-1} in the ISS orbit, a dose of 2×10^{12} would be accumulated after around 90 years. While this is true for solar cells mounted on the satellite surface, free-standing solar arrays are exposed from both sides, and therefore a dose of 2×10^{12} p^+ cm^{-2} would be reached earlier after about 50 years.

Supplemental References

1. Köhnen, E., Jošt, M., Morales-Vilches, A.B., Tockhorn, P., Al-Ashouri, A., Macco, B., Kegelmann, L., Korte, L., Rech, B., Schlatmann, R., et al. (2019). Highly efficient monolithic perovskite silicon tandem solar cells: analyzing the influence of current mismatch on device performance. Sustain. Energy Fuels *3*, 1995–2005.

2. Lang, F., Nickel, N.H., Bundesmann, J., Seidel, S., Denker, A., Albrecht, S., Brus, V. V., Rappich, J., Rech, B., Landi, G., et al. (2016). Radiation Hardness and Self-Healing of Perovskite Solar Cells. Adv. Mater. *28*, 8726–8731.

3. Jošt, M., Bertram, T., Koushik, D., Marquez, J.A., Verheijen, M.A., Heinemann, M.D., Köhnen, E., Al-Ashouri, A., Braunger, S., Lang, F., et al. (2019). 21.6%-Efficient Monolithic Perovskite/Cu(In,Ga)Se 2 Tandem Solar Cells with Thin Conformal Hole Transport Layers for Integration on Rough Bottom Cell Surfaces. ACS Energy Lett. *4*, 583–590.

4. Repins, I., Contreras, M.A., Egaas, B., DeHart, C., Scharf, J., Perkins, C.L., To, B., and Noufi, R. (2008). 19·9%-efficient ZnO/CdS/CuInGaSe 2 solar cell with 81·2% fill factor. Prog. Photovoltaics Res. Appl. *16*, 235–239.

5. Koushik, D., Jošt, M., Dučinskas, A., Burgess, C., Zardetto, V., Weijtens, C., Verheijen, M.A., Kessels, W.M.M., Albrecht, S., and Creatore, M. (2019). Plasma-assisted atomic layer deposition of nickel oxide as hole transport layer for hybrid perovskite solar cells. J. Mater. Chem. C *7*, 12532–12543.

6. Denker, A., Rethfeldt, C., Röhrich, J., Berlin, H., Cordini, D., Heufelder, J., Stark, R., Weber, A., and Berlin, B.H. (2010). Status of the Hzb # Cyclotron : Eye Tumour Therapy in Berlin Patient Numbers and Special. Proc. CYCLOTRONS 2010, (Lanzhou, China), 75–77.

7. Röhrich, J., Damerow, T., Hahn, W., Müller, U., Reinholz, U., and Denker, A. (2012). A Tandetron^{TM} as proton injector for the eye tumor therapy in Berlin. Rev. Sci. Instrum. *83*, 02B903.

8. Jasenek, A., Boden, A., Weinert, K., Balboul, M.R., Schock, H.W., and Rau, U. (2001). High-Energy Electron and Proton Irradiation of Cu(In,Ga)Se 2 Heterojunction Solar Cells. MRS Proc. *668*, H3.2.

9. Khatri, I., Lin, T.-Y., Nakada, T., and Sugiyama, M. (2019). Proton Irradiation on Cesium-Fluoride-Free and Cesium-Fluoride-Treated Cu(In,Ga)Se 2 Solar Cells and Annealing Effects under Illumination. Phys. status solidi – Rapid Res. Lett., 1900519.

10. Jasenek, A., Schock, H.W., Werner, J.H., and Rau, U. (2001). Defect annealing in Cu(In,Ga)Se2 heterojunction solar cells after high-energy electron irradiation. Appl. Phys. Lett. *79*, 2922–2924.

11. Mintairov, M.A., Kalyuzhnyy, N.A., Evstropov, V. V., Lantratov, V.M., Mintairov, S.A., Shvarts, M.Z., Andreev, V.M., and Luque, A. (2015). The segmental approximation in multijunction solar cells. IEEE J. Photovoltaics *5*, 1229–1236.

12. Delamarre, A., Lombez, L., and Guillemoles, J.F. (2012). Contactless mapping of saturation currents of solar cells by photoluminescence. Appl. Phys. Lett. *100*, 2012–2015.

13. Würfel, P. (1982). The chemical potential of radiation. J. Phys. C Solid State Phys. *15*, 3967–3985.

14. Unold, T., and Gütay, L. (2011). Advanced Characterization Techniques for Thin Film Solar Cells (JohnWiley & Sons, Ltd).

15. Ziegler, J.F., Ziegler, M.D.D., and Biersack, J.P.P. (2010). SRIM - The stopping and range of ions in matter (2010). Nucl. Instruments Methods Phys. Res. Sect. B Beam Interact. with Mater. Atoms *268*, 1818–1823.

16. Jones, E., Oliphant, T., Peterson, P., and Al., E. (2001). SciPy: Open source scientific tools for Python. http://www.scipy.org/.

17. Ostrouchov, C., Zhang, Y., and J. Weber, W. (2018). pysrim: Automation, Analysis, and Plotting of SRIM Calculations. J. Open Source Softw. *3*, 829.

18. SPENVIS -- Space Environment Information System, funded by the European Space Agency (ESA) (2019). http://www.spenvis.ome.be/spenvis.

19. Babaro, J.P., West, K.G., and Hamadani, B.H. (2016). Spectral response measurements of multijunction solar cells with low shunt resistance and breakdown voltages. Energy Sci. Eng. *4*, 372–382.

20. Stolterfoht, M., Wolff, C.M., Márquez, J.A., Zhang, S., Hages, C.J., Rothhardt, D., Albrecht, S., Burn, P.L., Meredith, P., Unold, T., et al. (2018). Visualization and suppression of interfacial recombination for high-efficiency large-area pin perovskite solar cells. Nat. Energy *3*, 847–854.

21. Lang, F., Jošt, M., Bundesmann, J., Denker, A., Albrecht, S., Landi, G., Neitzert, H.-C.C., Rappich, J., and Nickel, N.H. (2019). Efficient minority carrier detrapping mediating the radiation hardness of triple-cation perovskite solar cells under proton irradiation. Energy Environ. Sci. *12*, 1634–1647.

22. Arutyunov, N., Emtsev, V., Krause-Rehberg, R., Elsayed, M., Kessler, C., Kozlovski, V., and Oganesyan, G. (2015). Positron probing of phosphorus-vacancy complexes in silicon irradiated with 15 MeV protons. J. Phys. Conf. Ser. *618*, 8–12.

23. Yamaguchi, M., Khan, A., Taylor, S.J., Ando, K., Yamaguchi, T., Matsuda, S., and Aburaya, T. (1999). Deep level analysis of radiation-induced defects in Si crystals and solar cells. J. Appl. Phys. *86*, 217–223.

24. Karazhanov, S.Z. (2000). Effect of radiation-induced defects on silicon solar cells. J. Appl. Phys. *88*, 3941–3947.

25. Watkins, G.D. (2000). Intrinsic defects in silicon. Mater. Sci. Semicond. Process. *3*, 227–235.

26. Crabb, R.L. (1994). Solar cell radiation damage. Radiat. Phys. Chem. *43*, 93–103.

27. Hu, Z., He, S., and Yang, D. (2004). Effect of <200 keV proton radiation on electric properties of silicon solar cells at 77 K. Nucl. Instruments Methods Phys. Res. Sect. B Beam Interact. with Mater. Atoms *217*, 321–326.

28. Srour, J.R., Marshall, C.J., and Marshall, P.W. (2003). Review of displacement damage effects in silicon devices. IEEE Trans. Nucl. Sci. *50*, 653–670.

29. Neitzert, H.C., Spinillo, P., Bellone, S., Licciardi, G.D., Tucci, M., Roca, F., Gialanella, L., and Romano, M. (2004). Investigation of the damage as induced by 1.7 MeV protons in an amorphous/crystalline silicon heterojunction solar cell. Sol. Energy Mater. Sol. Cells *83*, 435–446.

30. Neitzert, H.-C., Ferrara, M., Kunst, M., Denker, A., Kertész, Z., Limata, B., Gialanella, L., and Romano, M. (2008). Electroluminescence efficiency degradation of crystalline silicon solar cells after irradiation with protons in the energy range between 0.8 MeV and 65 MeV. Phys. status solidi *245*, 1877–1883.

31. Hisamatsu, T., Kawasaki, O., Matsuda, S., Nakao, T., and Wakow, Y. (1998). Radiation degradation of large fluence irradiated space silicon solar cells. Sol. Energy Mater. Sol. Cells *50*, 331–338.

32. Imaizumi, M., Sumita, T., Kawakita, S., Aoyama, K., Anzawa, O., Aburaya, T., Hisamatsu, T., and Matsuda, S. (2005). Results of flight demonstration of terrestrial solar cells in space. Prog. Photovoltaics Res. Appl. *13*, 93–102.

33. Yamaguchi, M., Taylor, S.J., Yang, M.J., Matsuda, S., Kawasaki, O., and Hisamatsu, T. (1996). High-energy and high-fluence proton irradiation effects in silicon solar cells. J. Appl. Phys. *80*,

4916–4920.

34. Sahli, F., Werner, J., Kamino, B.A., Bräuninger, M., Monnard, R., Paviet-Salomon, B., Barraud, L., Ding, L., Diaz Leon, J.J., Sacchetto, D., et al. (2018). Fully textured monolithic perovskite/silicon tandem solar cells with 25.2% power conversion efficiency. Nat. Mater. *17*, 820–826.

35. Bush, K.A., Palmstrom, A.F., Yu, Z.J., Boccard, M., Cheacharoen, R., Mailoa, J.P., McMeekin, D.P., Hoye, R.L.Z., Bailie, C.D., Leijtens, T., et al. (2017). 23.6%-efficient monolithic perovskite/silicon tandem solar cells with improved stability. Nat. Energy *2*, 17009.

36. Jošt, M., Köhnen, E., Morales-Vilches, A.B., Lipovšek, B., Jäger, K., Macco, B., Al-Ashouri, A., Krč, J., Korte, L., Rech, B., et al. (2018). Textured interfaces in monolithic perovskite/silicon tandem solar cells: advanced light management for improved efficiency and energy yield. Energy Environ. Sci. *11*, 3511–3523.

37. Adey, J., Jones, R., Palmer, D.W., Briddon, P.R., and Öberg, S. (2004). Degradation of boron-doped Czochralski-grown silicon solar cells. Phys. Rev. Lett. *93*, 1–4.

38. Schmidt, J., and Bothe, K. (2004). Structure and transformation of the metastable boron- and oxygen-related defect center in crystalline silicon. Phys. Rev. B *69*, 024107.

39. Mailoa, J.P., Bailie, C.D., Johlin, E.C., Hoke, E.T., Akey, A.J., Nguyen, W.H., McGehee, M.D., and Buonassisi, T. (2015). A 2-terminal perovskite/silicon multijunction solar cell enabled by a silicon tunnel junction. Appl. Phys. Lett. *106*, 121105.

40. Lang, F., Shargaieva, O., Brus, V. V., Neitzert, H.C., Rappich, J., and Nickel, N.H. (2018). Influence of Radiation on the Properties and the Stability of Hybrid Perovskites. Adv. Mater. *30*, 1702905.

41. Köppel, G., Eisenhauer, D., Rech, B., and Becker, C. (2017). Combining tailor-made textures for light in-coupling and light trapping in liquid phase crystallized silicon thin-film solar cells. Opt. Express *25*, A467.

42. American Society for Testing and Materials (ASTM) Terrestrial Reference Spectra for Photovoltaic Performance Evaluation (2017). Reference Solar Spectral Irradiance Air Mass 1.5 ASTM G173. http://rredc.nrel.gov/solar/spectra/am1.5/.
