# Supplementary material for: Proton Radiation Hardness of Perovskite Tandem Photovoltaics
Source: Joule. 2020 May 20;4(5):1054–69. doi: 10.1016/j.joule.2020.03.006 (PMC7238692; doi:10.1016/j.joule.2020.03.006)
Supplement: Document S2. Article plus Supplemental Information [file mmc2.pdf]

Joule

CellPress

Article

Proton Radiation Hardness of Perovskite Tandem Photovoltaics

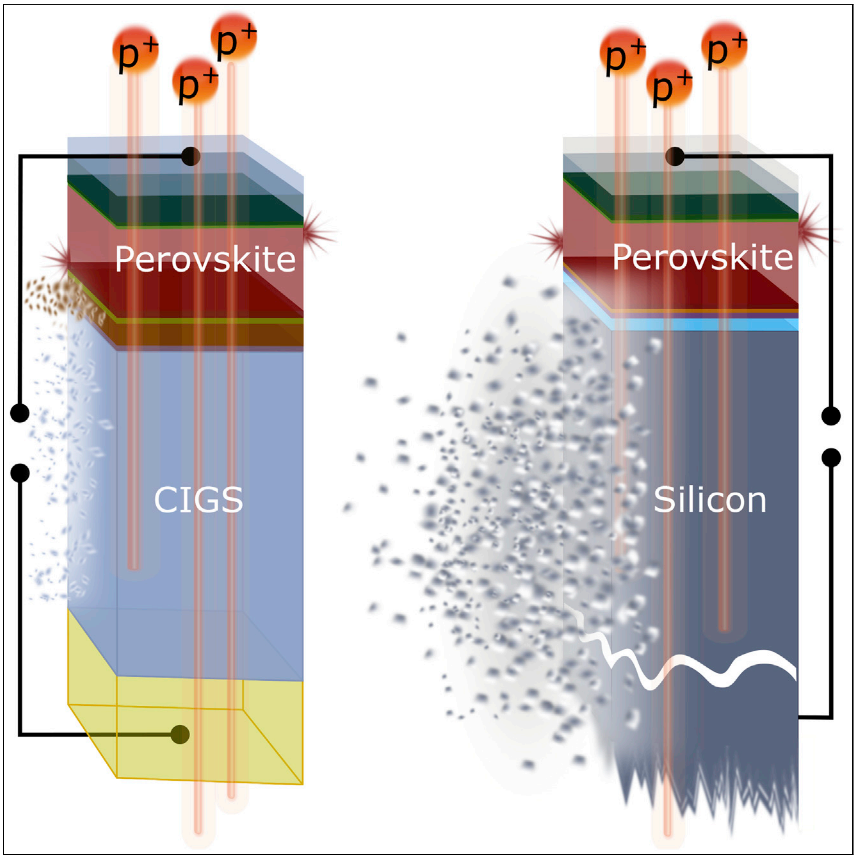

We propose and test monolithic perovskite/CIGS tandem solar cells for readily stowable, ultra-lightweight space photovoltaics. We design *operando* and *ex situ* measurements to show that perovskite/CIGS tandem solar cells retain over 85% of their initial power-conversion efficiency after high-energy proton irradiation. While the perovskite sub-cell is unaffected after this bombardment, we identify increased non-radiative recombination in the CIGS bottom cell and nickel-oxide-based recombination layer. By contrast, monolithic perovskite/silicon-heterojunction cells degrade to 1% of their initial efficiency due to radiation-induced defects in silicon.

Felix Lang, Marko Jošt, Kyle Frohna, ..., Heinz-Christoph Neitzert, Norbert H. Nickel, Samuel D. Stranks

fl396@cam.ac.uk (F.L.)
steve.albrecht@helmholtz-berlin.de (S.A.)
sds65@cam.ac.uk (S.D.S.)

HIGHLIGHTS

Halide perovskite sub-cells exhibit strong proton irradiation resiliency

Novel *operando* characterization distinguishes degradation of individual sub-cells

Perovskite/CIGS tandem solar cells retain 85% of their initial efficiency after irradiation

Perovskite/SHJ tandem solar cells degrade to 1% of their initial efficiency after irradiation

Lang et al., Joule 4, 1054–1069
May 20, 2020 © 2020 The Authors. Published by Elsevier Inc.
https://doi.org/10.1016/j.joule.2020.03.006

CellPress OPEN ACCESS

Joule

Article

Proton Radiation Hardness of Perovskite Tandem Photovoltaics

Felix Lang,[1,*] Marko Jošt,[2] Kyle Frohna,[1] Eike Köhnen,[2] Amran Al-Ashouri,[2] Alan R. Bowman,[1] Tobias Bertram,[3] Anna Belen Morales-Vilches,[3] Dibyashree Koushik,[4] Elizabeth M. Tennyson,[1] Krzysztof Galkowski,[1,5] Giovanni Landi,[6] Mariadriana Creatore,[4] Bernd Stannowski,[3] Christian A. Kaufmann,[3] Jürgen Bundesmann,[7] Jörg Rappich,[8] Bernd Rech,[8,9] Andrea Denker,[7,10] Steve Albrecht,[2,9,*] Heinz-Christoph Neitzert,[6] Norbert H. Nickel,[8] and Samuel D. Stranks[1,11,12,*]

SUMMARY

Monolithic $[Cs_{0.05}(MA_{0.17}FA_{0.83})_{0.95}]Pb(I_{0.83}Br_{0.17})_3/Cu(In,Ga)Se_2$ (perovskite/CIGS) tandem solar cells promise high performance and can be processed on flexible substrates, enabling cost-efficient and ultra-lightweight space photovoltaics with power-to-weight and power-to-cost ratios surpassing those of state-of-the-art III-V semiconductor-based multijunctions. However, to become a viable space technology, the full tandem stack must withstand the harsh radiation environments in space. Here, we design tailored *operando* and *ex situ* measurements to show that perovskite/CIGS cells retain over 85% of their initial efficiency even after 68 MeV proton irradiation at a dose of 2×10^{12} p^+/cm^2. We use photoluminescence microscopy to show that the local quasi-Fermi-level splitting of the perovskite top cell is unaffected. We identify that the efficiency losses arise primarily from increased recombination in the CIGS bottom cell and the nickel-oxide-based recombination contact. These results are corroborated by measurements of monolithic perovskite/silicon-heterojunction cells, which severely degrade to 1% of their initial efficiency due to radiation-induced recombination centers in silicon.

Context & Scale

Monolithic perovskite/silicon and perovskite/CIGS tandem solar cells could facilitate large-scale decarbonization of the power sector, provided their long-term stability is proven. In this work, we test the stability of both technologies under high-energy proton irradiation. While this mimics the radiation environment in space, our versatile *operando* and *ex situ* methodology is also suitable for studying the long-term stability of multijunction solar cells for terrestrial applications. We find that perovskite/silicon tandem solar cells are unsuitable for space, whereas perovskite/CIGS tandems are radiation-hard, promising cheap, flexible, and ultra-lightweight space photovoltaics. Both the growing demand for smaller, cheaper satellites and the privatization of space exploration are revolutionizing space economics, providing an ideal niche for the commercialization of this new technology until the levelized cost-of-electricity can compete with current terrestrial photovoltaics.

INTRODUCTION

Multijunction solar cells that combine complementary absorber materials to selectively harvest the available solar spectrum with minimal thermalization losses power modern energy demanding satellites, spacecraft, and exploration rovers.[1] While terrestrial photovoltaic (PV) systems require high-power-area (W/m^2) ratios, space PV systems also require high specific power (W/g) to minimize the stowed volume, weight, inertia, and atmospheric drag of the spacecraft.[2] In addition, the cost of space PV modules ($/W) is becoming increasingly important given the growing demand for smaller, cheaper satellites[3] and the emerging privatization of space exploration,[4] both of which are revolutionizing space economics. Furthermore, only lower costs will allow large-scale space explorations, including planned habitats on the Moon and Mars.[4] Triple- and quadruple-junction solar cells comprised of GaInP/GaAs/Ge or AlInGaP/AlInGaAs/InGaAs/Ge absorbers are today's state-of-the-art commercially available systems, reaching power-conversion efficiencies of 32%[5,6] under space solar illumination conditions (AM0). However, slow epitaxial absorber growth and high material costs render such III-V-based multijunction solar cells expensive and their mass production challenging.[7] Less expensive space-tested single-junction technologies based on crystalline silicon (c-Si),[8,9] $Cu(In,Ga)Se_2$ (CIGS),[10]

 © 2020 The Authors. Published by Elsevier Inc.
This is an open access article under the CC BY license (http://creativecommons.org/licenses/by/4.0/).

CellPress
OPEN ACCESS

halide perovskites,[11–13] or organic absorbers[14] do not meet the performance requirements of sufficiently high W/g and W/m^2 to compete with the III-V multijunction technologies. Compositionally engineered perovskites, with a band gap (E_G) of 1.6–1.8 eV can be processed on top of c-Si (E_G = 1.1 eV)[15–19] and CIGS (E_G ~1.1 eV)[20–22] absorbers, enabling monolithic tandem solar cells with efficiencies that surpass the limiting values of individual sub-cells. The first technology is close to commercialization for terrestrial applications,[23] while the latter technology can be processed on flexible foils to enable high W/g and W/m^2 values at low cost. Thus, perovskite-based multijunction PV has the potential to be a disruptive technology both on the Earth and in Space.

A crucial requirement for adoption is that the cells can withstand the harsh radiation environment of space without additional engineering solutions that add cost and compromise performance metrics. Accelerated by coronal mass ejections and solar flares, solar energetic particles consist mainly of protons (p^+) and electrons with kinetic energies ranging from keV to GeV.[24] High-energetic protons are about two orders of magnitude more damaging than highly energetic electrons.[12] Moreover, protons with energies above 1 MeV cannot be easily shielded and consequently damage electronic devices,[24] such as solar cells, which eventually leads to device failure. Promising test results from perovskite-based single-junction solar cells have revealed that devices under proton irradiation retained over 90% of their initial performance even after high proton fluences of 10^{12} p^+/cm^2 [13] and $10^{14} p^+/cm^2$ [12] with proton energies of 0.05–68 MeV.[11–13,25,26] However, monolithic tandem solar cells are connected in series and, hence, radiation-induced damage in just one of the sub-cells can degrade the performance of the entire tandem solar cell. Therefore, to validate these technologies, studies are required on the entire tandem systems during operation. Here, we reveal the suitability of state-of-the-art monolithic perovskite/CIGS tandem solar cells to power satellites and spacecraft by testing their radiation hardness *in operando* under 68 MeV proton (p^+) irradiation.

RESULTS

Perovskite/CIGS and Perovskite/Silicon Tandem Solar Cells

The investigated perovskite/CIGS and perovskite/silicon tandem solar cells utilize triple cation perovskite absorber layers $[Cs_{0.05}(MA_{0.17}FA_{0.83})_{0.95}]Pb(I_{0.83}Br_{0.17})_3$ with a band gap of E_G = 1.62 eV (Figures 1A and 1B). In both cases, we employ an inverted p-i-n configuration and sandwich the perovskite absorber between poly[bis(4-phenyl)(2,4,6-trimethylphenyl)amine] (PTAA) and C_{60} layers that act as hole- and electron-selective layers, respectively. To avoid the influence of oxygen and moisture[18] all tandems were air-to-N_2 encapsulated using a radiation-hard quartz substrate, which leads to additional reflection losses of ~7% that could be ultimately removed using more suitable encapsulation techniques. The stabilized efficiency and power output of the quartz-encapsulated perovskite/CIGS solar cells here, thus, amounts to 18% and ~180 W/m^2, respectively, under irradiation with a terrestrial solar spectrum AM1.5G (1,000 W/m^2). The stabilized power output increases to ~202 W/m^2 with an efficiency of 15.1% under space AM0 spectral conditions (1,350 W/m^2). The perovskite/CIGS tandem solar cells have a combined active layer thickness of 4.38 μm and a very low specific weight of just 2.8 mg/cm^2, yielding an excellent specific-power of 7.4 W/g. We note that these values do not account for commonly employed encapsulation glasses and substrates. Assuming a 25-μm thick substrate and encapsulation foil often used for flexible CIGS and perovskite solar cells,[27] the specific power is 2.1 W/g, a factor of ~3 times larger than those of typically used GaInP/GaAs/Ge absorbers at 0.8 W/g[5] and expected improvements in

[1]Cavendish Laboratory, Department of Physics, University of Cambridge, Cambridge CB30HE, UK
[2]Young Investigator Group Perovskite Tandem Solar Cells, Helmholtz-Zentrum Berlin für Materialien und Energie GmbH, Berlin 12489, Germany
[3]PVcomB, Helmholtz-Zentrum Berlin für Materialien und Energie GmbH, Berlin 12489, Germany
[4]Plasma and Materials Processing, Department of Applied Physics, Eindhoven University of Technology, (TU/e), Eindhoven, 5600 MB, the Netherlands
[5]Institute of Physics, Faculty of Physics, Astronomy and Informatics, Nicolaus Copernicus University, Toruń, 87-100, Poland
[6]Department of Industrial Engineering, (DIIn), Salerno University, Fisciano, SA 84084, Italy
[7]Helmholtz-Zentrum Berlin für Materialien und Energie GmbH, und Energie GmbH, Protonen für die Therapie, Berlin 14109, Germany
[8]Institute for Silicon Photovoltaics, Helmholtz-Zentrum Berlin für Materialien und Energie GmbH, Berlin 12489, Germany
[9]Technical University Berlin, Faculty IV, Electrical Engineering and Computer Science, Berlin, Germany
[10]Beuth Hochschule für Technik Berlin, Fachbereich II – Mathematik – Physik – Chemie, Luxemburgerstr. 10, Berlin 13353, Germany
[11]Department of Chemical Engineering & Biotechnology, University of Cambridge, Cambridge CB3 0AS, UK
[12]Lead Contact
*Correspondence: fl396@cam.ac.uk (F.L.), steve.albrecht@helmholtz-berlin.de (S.A.), sds65@cam.ac.uk (S.D.S.)
https://doi.org/10.1016/j.joule.2020.03.006

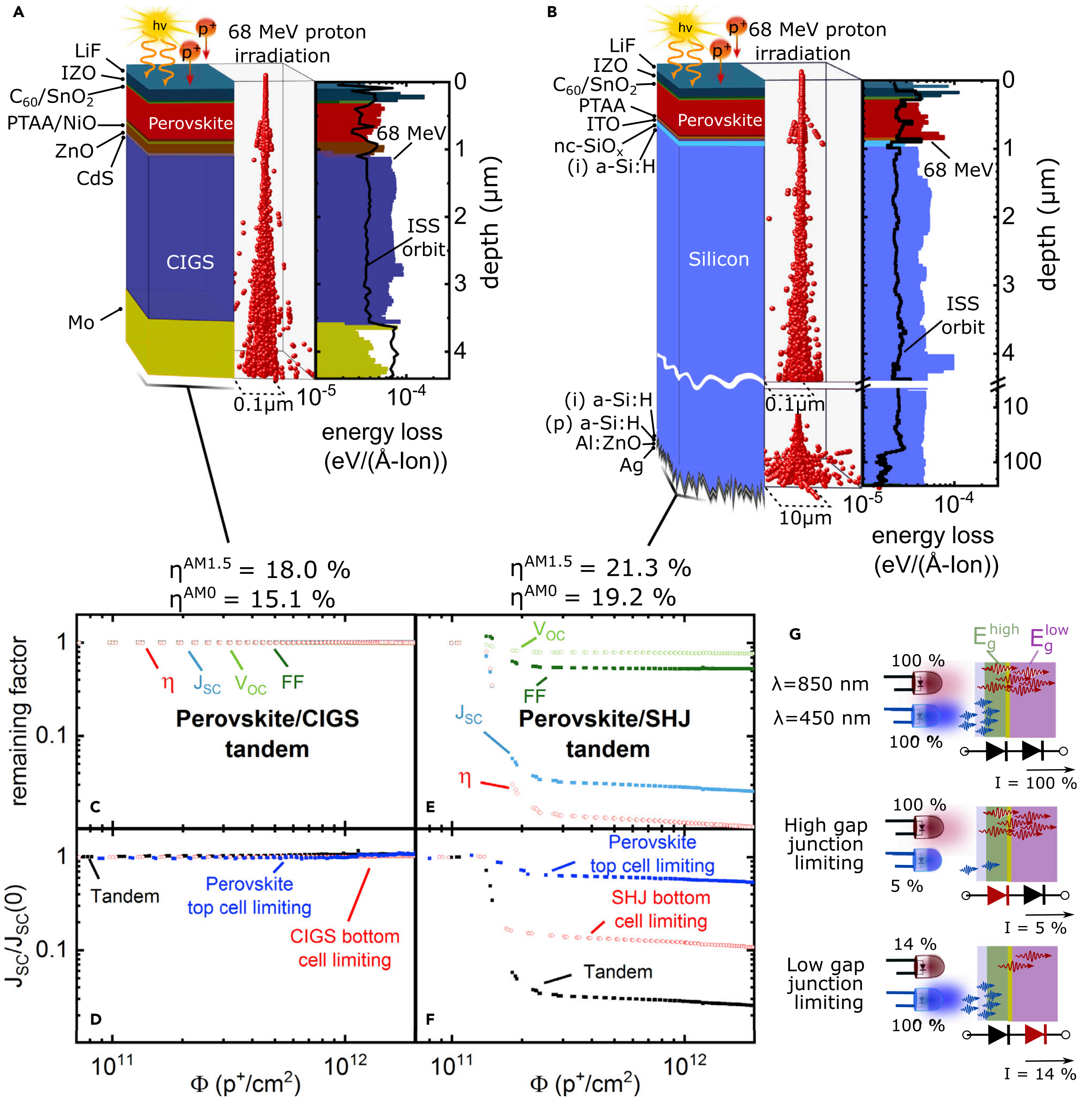

Figure 1. Probing the Radiation Hardness of Perovskite/SHJ and Perovskite/CIGS Tandem Solar Cells *In Operando* during Proton Irradiation

(A and B) 3D scatter plots of the straggling of 68 MeV protons within the perovskite/CIGS (A) and perovskite/SHJ (B) tandem solar cells. The corresponding energy loss of the incident 68 MeV protons to recoils is plotted as a function of depth based on SRIM simulations with a total of 5×10^7 protons. The damage of a real space environment at the orbit of the international space station (ISS) is shown as black line considering polyenergetic and omnidirectional proton irradiation (see Supplemental Information for further details).

(C and E) *Operando* measurements of V_{OC}, J_{SC}, FF, and η of the investigated perovskite/CIGS (C) and perovskite/SHJ (E) tandem solar cell as a function of the accumulated proton dose Φ. All values are normalized to their initial value. The proton energy amounted to 68 MeV.

(D–G) Normalized short-circuit current of perovskite/CIGS (D) and perovskite/SHJ (F) tandem solar cell under illumination with NIR (λ = 850 nm) and blue LEDs (λ = 450 nm) that were alternatingly set to either 100% or 5/14% (see Supplemental Information for further details) to mimic current matching under AM0 or forcing one sub-cell into limitation as illustrated in (G).

CellPress
OPEN ACCESS

efficiency will increase this factor further. For the monolithic perovskite/silicon tandem solar cells, we utilize a rear emitter c-Si (n) silicon heterojunction (SHJ) with planar front and textured backside. The stabilized efficiency and power output of the quartz-encapsulated perovskite/SHJ cells reaches 21.3% and ~213 W/m^2, respectively, under AM1.5G irradiation, increasing to ~257 W/m^2 with an efficiency of 19.2% under AM0. The perovskite/SHJ tandem solar cell is based on an active layer with a combined thickness of 261.5 μm and a specific weight of 61 mg/cm^2, yielding a specific-power of 0.42 W/g (excluding encapsulation glass), which is comparable to the triple-junction technologies in terms of specific power while also promising much lower power module costs ($/W), albeit without the flexible form factor that CIGS and perovskites offer.

Proton-Irradiation-Induced Damage Profile

We employ 68 MeV proton irradiation to represent a key problematic energy range that will penetrate through sufficiently inexpensive encapsulating engineering solutions, standardly employed to shield low-energy protons (E < 1 MeV), including the quartz substrate used here. To ascertain damage in the layer stacks due to electronic and nuclear scattering, we used a Monte Carlo based simulation of the stopping and range of ions in matter (SRIM).[28] Electronic scattering of the incident proton ionizes the target material and can lead to the disruption of C–H and N–H bonds in the organic and hybrid perovskite layers in the device stacks.[29,30] Nuclear scattering of the incident proton causes the target nuclei to recoil and be displaced, which instigates a cascade of damage events that generate defects in the material. In Figures 1A and 1B, we plot the simulated straggling as well as the energy transferred to the recoiling nuclei that is typically used as a measure for the degradation of PV parameters.[31] In both the investigated perovskite/SHJ and perovskite/CIGS stacks, we observe a uniform damage profile throughout the layer stacks, therefore, allowing us to probe the impact of irradiation on the entire tandem stack with this proton energy. We also compare the damage profile of our monoenergetic and monodirectional 68 MeV proton irradiation to the damage expected from poly-energetic omnidirectional proton irradiation in the orbit of the International Space Station (ISS) (Figures 1A and 1B, black line; see Figure S1 in the Supplemental Information for details), revealing a similarly uniform profile. Thus, these experiments replicate a real space environment in which both sub-cells and all involved interlayers are damaged comparably. Non-uniform damage confined to the topmost layers from low-energy proton testing (E< 1 MeV) will not be seen in orbit,[32] while as we identify later, some of the most problematic layers are buried recombination contacts. Our SRIM simulations further suggest that the proton dose of 2 × 10^{12}p$^+$/cm^2 is equivalent to the accumulated dose on the tandem solar cells after more than 50 years in an ISS orbit (see Supplemental Information for details).

Probing the Radiation Hardness *In Operando*

To assess the radiation hardness of the investigated tandem solar cells, we tracked *in operando* the evolution of the PV parameters during 68 MeV proton irradiation as a function of the accumulated proton dose Φ. Using an incident fluence of ~7 × 10^8 p$^+$/cm^2/s the highest dose of Φ = 2 × 10^{12} p$^+$/cm^2 was reached after ~1 h of operation. For the *operando* measurements, the active area of 0.81 cm^2 was homogeneously illuminated by two high-intensity LEDs equivalent to ~¼ AM0 with wavelengths tailored to selectively illuminate the high gap or the low gap sub-cells, respectively (λ^1_{LED} = 450 nm and λ^2_{LED} = 850 nm). In contrast to conventional white light LEDs, this allowed us to mimic current matching between the two sub-cells equivalent to them being under AM1.5G or AM0 conditions (Figure 1G, top). In Figure 1C, we display the evolution of the open-circuit voltage (V_{OC}), fill factor (FF),

short-circuit current density (J_{SC}), and power-conversion efficiency (η) of the perovskite/CIGS tandem solar cell, with each metric normalized to its initial value (see Figures S3 and S4 for un-normalized JV characteristics). The *operando* data reveal only minor degradation in V_{OC} to about 99% of its initial value and no significant degradation in FF, J_{SC}, or η for a proton dose of 2×10^{12} p^+/cm^2. By contrast, we observe that the perovskite/SHJ tandem solar cell (Figure 1E) degrades rapidly to an efficiency of only 1% of its initial value after an accumulated proton dose of only $\Phi \sim 10^{11}$ p^+/cm^2. Notably, the degradation of the perovskite/SHJ tandem is dominated by the J_{SC} that decreases to about 2% of its initial value, and the performance parameters do not recover after removing the proton irradiation (Figure S2).

To further investigate each sub-cell during proton irradiation, we periodically decreased the intensity of one LED to ~5% or 14% of its output power such that one of the sub-cells limits the overall current (Figure 1G). In the case of the perovskite/SHJ tandem solar cell limited by the SHJ bottom cell, the J_{SC} decreases rapidly (Figure 1F), strongly suggesting that the degradation of the perovskite/SHJ tandem is dominated by the SHJ sub-cell. However, the data also suggest some losses within the perovskite sub-cell. By contrast, neither the perovskite nor the CIGS sub-cell degrades under the same irradiation dose in the case of the perovskite/CIGS tandem solar cell (Figure 1D).

Radiation-Hard Perovskite/CIGS Tandem Solar Cells

In order to understand the behavior of the irradiated cells, the devices were characterized prior to and after the proton irradiation. We note that the post-irradiation measurements were performed after the activity of generated short-living isotopes in the irradiated samples dropped to a safe level of less than 10^3 Bq (~10 days of storage). In Figure 2A, we show current-voltage (JV) measurements of the perovskite/CIGS tandem under AM0 and AM1.5G conditions taken prior to and after 68 MeV proton irradiation. Notably, the power output remains high, and the perovskite/CIGS tandem solar cell retains ~85% of its initial performance under AM0 illumination, with the power output decreasing from ~202 to ~173 W/m^2. Under AM1.5G illumination, the perovskite/CIGS tandem solar cell retains over ~83% of its initial performance (see Table S1 for parameters). The JV curves show that the small but detectable radiation-induced performance losses primarily originate from a reduction in V_{OC} and FF. Notably, J_{SC} remains high. The findings are corroborated by measuring the spectral response of both sub-cells using appropriate light and voltage biases (Figure 2B). The external quantum efficiency (EQE) of the perovskite sub-cell increases slightly, from a maximum value of 83% before irradiation to 85% after proton irradiation, which we discuss further below. On the other hand, the EQE of the CIGS sub-cell decreases from a maximum value of 84% before irradiation (solid red line) to 77% after proton irradiation (red dashed line) when measured under identical light biasing conditions. By employing stronger light biasing and higher chopper frequencies, however, the CIGS sub-cell EQE could be recovered up to a maximum value of 86% (red dotted lines). We propose that this dependence on the frequency and light biasing is a combination of radiation-induced trap states and a low shunt resistance of the CIGS bottom cell (see Figures S19 and S4 for JV characteristics in the dark and under 14% 850 nm, 100% 450 nm LED illumination), and we also note that spectral response measurements of multijunction cells are challenging after particle irradiation or when one sub-cell under test features non-ideal properties.[33,34] This is exemplified by the fact there is no loss in EQE in identically prepared and irradiated CIGS single-junction solar cells (Figure S11). The radiation-induced trap states in the perovskite/CIGS tandem solar cell lead to a decrease in the open-circuit voltage of ΔV_{OC} = 0.02 V.

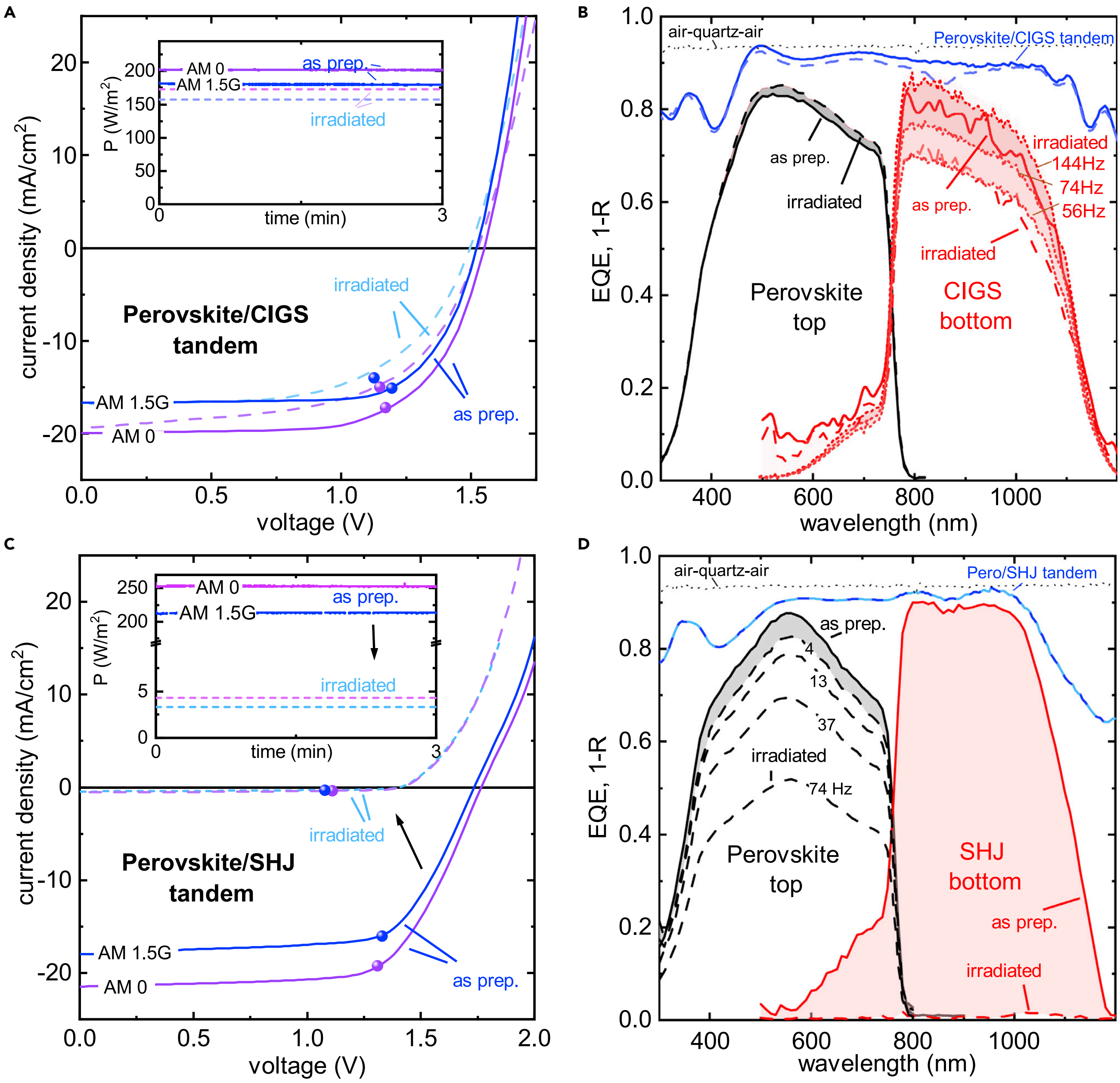

Figure 2. Proton-Irradiated Perovskite/SHJ and Perovskite/CIGS Tandem Solar Cells

(A and C) Current-voltage characteristics of as-prepared (solid lines) and proton irradiated (dashed lines, $\Phi = 2 \times 10^{12}$ p^+/cm^2, $E_p = 68$ MeV) perovskite/CIGS (A) and perovskite/SHJ (C) tandem solar cell under AM1.5G and AM0 illumination. The full circles indicate the mean maximum power point (MPP), and the inset depicts the power output at MPP as a function of time.

(B and D) External quantum efficiency of the perovskite and the CIGS sub-cell (B) before (solid lines) and after proton irradiation (dashed lines). The EQE was measured using a chopper frequency of 74 Hz and appropriate LEDs to light bias the tandem. In the case of the CIGS bottom cell, EQE measurements were also performed employing higher chopper frequencies and stronger light biasing from a halogen lamp equipped with appropriate filters as indicated. In case of the perovskite/SHJ tandem (D), the irradiated perovskite top cell was also measured employing lower chopper frequencies as indicated. In both cases, the reflection of the tandem solar cells is shown by the blue solid (as-prepared) and dashed (irradiated) lines. The dotted lack line depicts the reflection of the used air-quartz-air encapsulation.

By contrast, the JV measurements of the perovskite/SHJ cell shown in Figure 2C reveal a large decrease in all PV parameters upon proton irradiation. While the V_{OC} and FF decrease by ~20% relative to their initial value, it is the short-circuit current density

J_{SC} that shows a dramatic reduction from 21.5 to only 0.3 mA/cm^2 (under AM0 conditions) and, consequently, the stabilized power output is reduced from ~257 to only 4 W/m^2. These findings are also reflected in the changes of the maximum EQE of the SHJ sub-cell (Figure 2D), which drops from 90% to 1.5% after proton irradiation. This shows unambiguously that the SHJ bottom cell is heavily damaged, consistent with previous works on single-junction c-Si technologies,[9,35–37] with the SHJ cell thereby limiting the overall perovskite/SHJ tandem solar cell. However, we observe that the maximum EQE of the perovskite sub-cell also drops from 87% to 50%, though the maximum EQE can be recovered to 82% as the optical chopper frequency for the measurement is reduced from 74 to 4 Hz. We propose that this frequency dependence is an artifact generated by the heavily damaged SHJ sub-cell, which limits the overall current and, thus, the spectral response of the perovskite sub-cell despite using appropriate light biases during fast measurements, but this effect can be mitigated during slower (lower frequency) measurements. Indeed, the photoconductivity of silicon can be sensitive to the emptying and filling of shallow and deep traps, occurring on timescales between 10^{-2} and 10^{1} s, respectively.[38] Further evidence from selectively probed photoluminescence measurements that allows one to exclude the radiation-induced degradation of the perovskite sub-cell in both the perovskite/CIGS and perovskite/SHJ tandem is discussed further below and summarized in Figure 5.

Optical Characterization of the Radiation-Induced Damage

To assess the V_{OC} losses of the perovskite/CIGS tandem solar cell, we selectively probed both sub-cells using steady-state and time-resolved photoluminescence (PL and TRPL) measurements that are sensitive to unwanted non-radiative recombination power loss pathways, for example, due to the presence of radiation-induced recombination centers. These optical measurements were carried out on proton-irradiated regions and non-irradiated regions on the same devices. The PL from the CIGS sub-cell, measured by selectively photo-exciting the CIGS layer using a NIR excitation at λ = 910 nm that passes through the perovskite top cell as displayed in Figure 3C, shows a lower PL intensity than before irradiation (Figure 3A), consistent with the presence of radiation-induced defects within the CIGS sub-cell. This is corroborated by a slightly faster TRPL decay after irradiation, which we measure using a pulsed λ = 636 nm excitation and appropriate long-pass filters to selectively detect PL from the CIGS sub-cell (Figure 3B). The perovskite top-cell PL and TRPL were probed using excitation wavelengths of λ = 405 nm and λ = 636 nm, respectively, that are predominantly absorbed in the perovskite sub-cell, as illustrated in Figure 3F. Surprisingly, we observe a slight increase in PL intensity (Figure 3E) and a prolongation of the TRPL decay (Figure 3D) after proton irradiation. Hence, the collective optical and device data suggest that the degradation of the perovskite/CIGS tandem under proton irradiation originates from damage in the CIGS sub-cell. We performed similar optical measurements on perovskite/SHJ tandem solar cells and found a vast decrease of PL intensity paired with a quenched TRPL decay in the SHJ sub-cell after proton irradiation (Figure S9), reaffirming that proton irradiation primarily causes damage in the SHJ bottom cell.

Photoluminescence Mapping of the Perovskite Sub-cell

In contrast to single-crystalline absorbers, spatial heterogeneities in composition, crystallinity, defect density, and optoelectronic properties pervade solution-processed perovskites.[39] To assess the impact of proton irradiation on the local properties of the perovskite sub-cell, we employed confocal photoluminescence lifetime mapping with high spatial resolution (~300 nm). In Figure 4B, we depict a lifetime map of a non-irradiated cell under pulsed (~10 sun equivalent) excitation, revealing an average lifetime of τ = 7.5 $\pm$ 1.0 ns and heterogeneous features at length scales on the order of 1 μm, a length scale about 2–3 times larger compared to typical grain

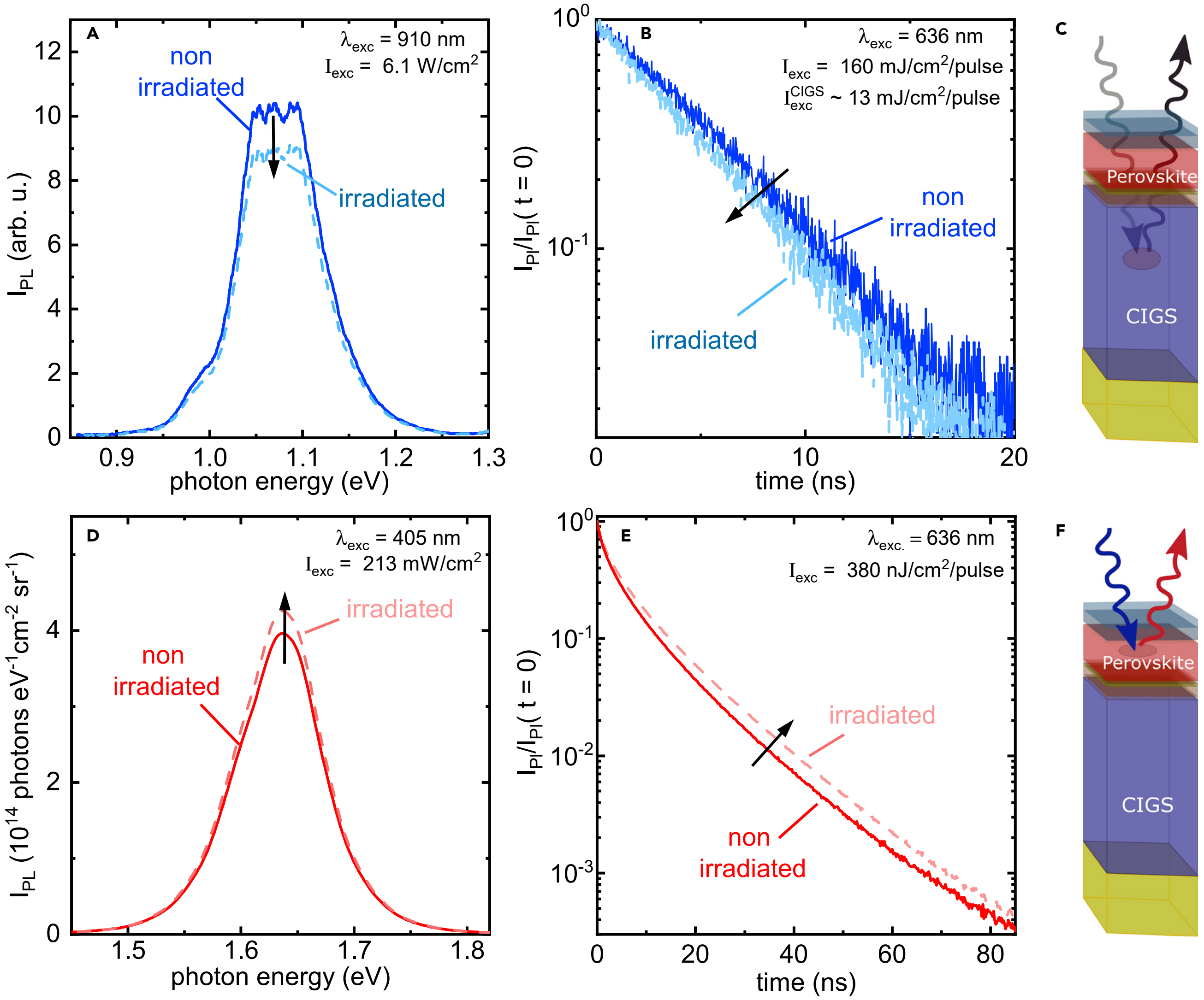

Figure 3. Identification of Radiation-Induced Recombination Pathways in Perovskite/CIGS Tandem Solar Cells after Proton Irradiation
(A–C) Photoluminescence spectra (A) and decay (B) of the non-irradiated and irradiated CIGS bottom absorber.
As sketched in (C), selective excitation in the CIGS layer was performed through the perovskite top absorber employing either a NIR cw laser at λ = 910 nm (in A) or a pulsed λ = 636 nm laser (in B) at a fluence of 160 mJ/cm^2 of which 13 mJ/cm^2 are absorbed within the CIGS in combination with appropriate long-pass filters to detect the emission.
(D–F) Photoluminescence spectra (D) and decay (E) of the non-irradiated and irradiated perovskite top absorber. Excitation was performed using cw 405 nm (in D) or pulsed 636 nm (in E) illumination at 380 nJ/cm^2, as shown in (F).

sizes estimated from scanning electron micrographs.[20] In accordance with TRPL data shown in Figure 3E, we identify a prolongation of the mean lifetime after irradiation to τ = 9.9 $\pm$ 1.3 ns accompanied by a slight increase in heterogeneity (Figure 4C), as seen in the right-skewed section of the histogram (Figures 4A and S13C). As the PL decay of halide perovskites is strongly quenched when sandwiched between charge selective contacts, the observed prolongations likely arise from radiation-induced changes at these interfaces, which we discuss further below.[40]

The spontaneous emission of photons from a direct semiconductor is, according to Würfels' generalized Planck law,[41] a function of the chemical potential of the non-equilibrium charge carrier concentration. This concentration corresponds to

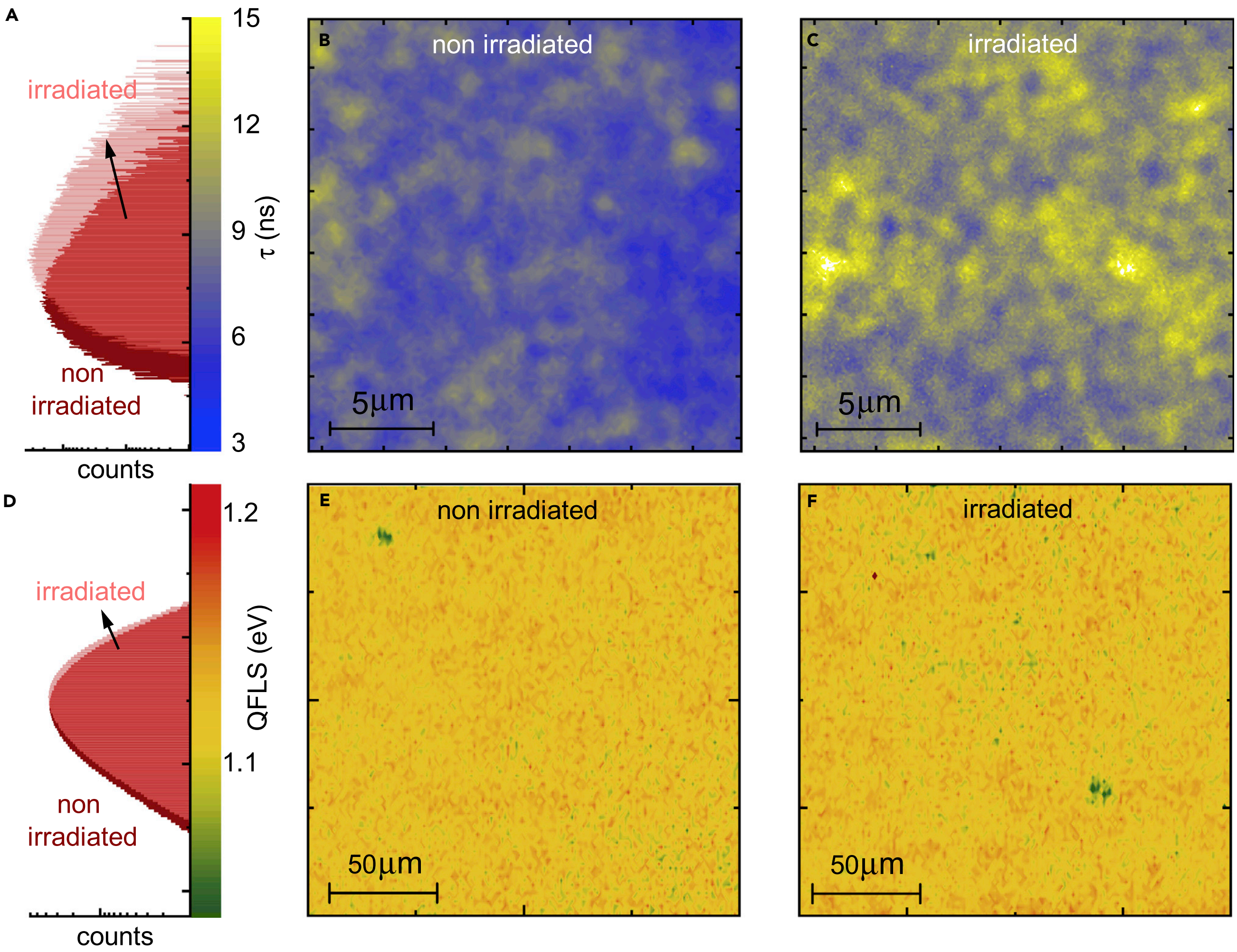

Figure 4. Photoluminescence Lifetime and Quasi-Fermi-Level-Splitting Mapping of the Perovskite

(A–C) (A) Photoluminescence lifetime histogram and (B and C) TRPL lifetime maps of the perovskite top absorber in the as-prepared and proton-irradiated perovskite/CIGS tandem solar cell under excitation with a 636 nm pulsed laser (5 MHz repetition rate, 380 nJ/cm^2/pulse fluence). Lifetimes were extracted using single-exponential fitting.

(D–F) (D) QFLS histogram and (E and F) QFLS maps of the perovskite top absorber in the as-prepared and proton-irradiated perovskite/CIGS tandem solar cell measured under 405 nm cw laser illumination with an intensity equivalent to 1 sun (see Supplemental Information for details).

the quasi-Fermi-level splitting (QFLS) of photo-excited electrons and holes, a quantity that translates to open-circuit voltage in a solar cell. By probing the absolute PL spectrum, we can thus extract the QFLS.[42] Here, we employ absolute hyperspectral PL imaging with high spatial resolution (~1 μm) to record the local QFLS of the perovskite sub-cell under equivalent excitation carrier densities to 1 sun (AM1.5G, see Supplemental Information for further details). In Figures 4E and 4F, we show QFLS maps of an irradiated and a non-irradiated device, respectively, along with their associated number histograms in Figure 4D (see Figure S14 for the corresponding PL maps and local PL spectra of selected locations). We find that there is an insignificant shift of the mean QFLS from 1.120 ± 0.022 eV (non-irradiated) to 1.124 ± 0.024 eV (irradiated), reiterating the excellent radiation hardness of the perovskite sub-cell.

Origin of Radiation-Induced V_{OC} Losses

To connect these results with the device measurements, we compare in Figure 5A the perovskite sub-cell QFLS to measured device V_{OC} values of the perovskite/CIGS

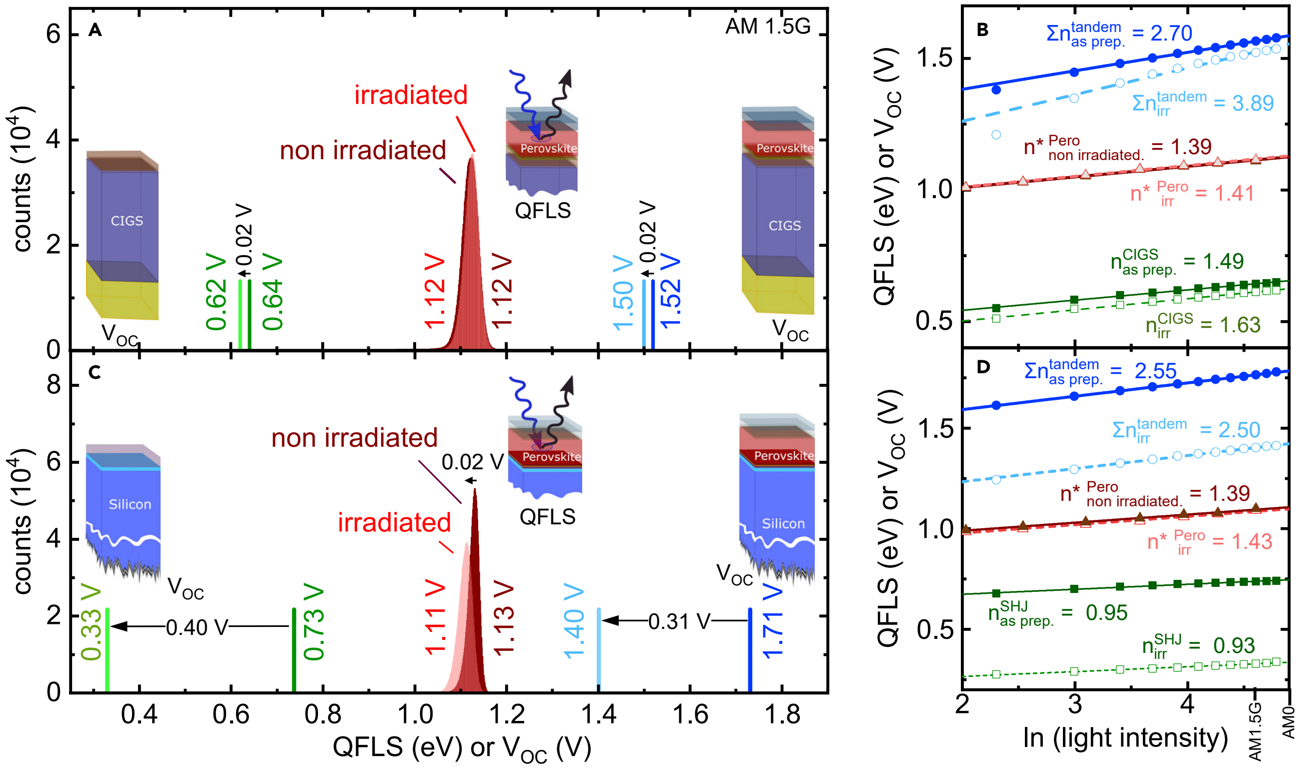

Figure 5. Radiation-Induced V_{OC} Losses in Perovskite/CIGS and Perovskite/SHJ Tandem Solar Cells

(A and C) Comparison of perovskite top cell QFLS statistics with the V_{OC} of perovskite/CIGS (A) and perovskite/SHJ (C) tandem and identically prepared CIGS and SHJ single-junction solar cells before and after irradiation, respectively.

(B and D) V_{OC} as a function of light intensity for as-prepared and proton-irradiated perovskite/CIGS (B) and perovskite/SHJ (D) tandem solar cells, as well as CIGS and SHJ single-junction solar cells, respectively. Open and closed triangles depict the QFLS of the perovskite sub-cell as a function of excitation fluence for the perovskite/CIGS and perovskite/SHJ tandem solar cells. n* denotes the internal ideality factor derived from Suns-QFLS statistics.

tandem and a CIGS single-junction cell; both measured before and after proton irradiation. The V_{OC} loss of the perovskite/CIGS tandem of ΔV_{OC} = 0.02 V matches the V_{OC} loss observed in a CIGS single-junction device. This comparison confirms that the V_{OC} losses in the tandem arise from losses in the CIGS bottom cell, consistent with the decrease in PL intensity (cf. Figure 3A), reiterating the resilience of the perovskite sub-cell to proton irradiation. To investigate this further, we perform intensity-dependent-V_{OC} measurements (Suns-V_{OC}) of the perovskite/CIGS tandem and the CIGS single-junction cells and extract the ideality factor n that is indicative of the dominant recombination mechanisms, i.e., $n = 1$ for ideal band-to-band recombination, $n = 2$ in the presence of deep recombination centers causing Shockley-Read-Hall recombination and $n < 1$ for Auger recombination under high injection conditions.[43] The Suns-V_{OC} slope of a monolithic tandem solar cell approximately equals the sum of the sub-cell ideality factors, $S = \sum_i n_i k_B T_i \approx k_B T \sum_i n_i$, assuming the individual sub-cells are at the same temperature of T = 300 K as set using a temperature-controlled stage (see Supplemental Experimental Procedures for details). Here k_B is the Boltzmann constant, and T is the temperature. As shown in Figure 5B, we estimate an $\sum_i n_i$ of 2.7 and 3.89 on as-prepared and proton-irradiated perovskite/CIGS tandem solar cells, respectively. This significant increase in the summed ideality factor suggests the presence of radiation-induced recombination centers in at least one of the sub-cells. Intensity-dependent-V_{OC} measurements on identically prepared and irradiated CIGS single-junction solar cells indicate an increase in the

ideality factor from 1.43 to 1.69 after proton irradiation. This observation indicates the presence of radiation-induced recombination centers, which is consistent with the observed decrease in V_{OC} and PL intensity (see Figures 2A and 3A). However, this increase in ideality factor is still somewhat smaller compared to the increase observed in the perovskite/CIGS tandem solar cell, which indicates the existence of an additional recombination pathway in the perovskite/CIGS tandem leading to FF loss after irradiation that is not present in the equivalent single-junction cells. We performed absolute PL measurements of the perovskite sub-cell while varying the excitation intensity, which allows us to extract the intensity-dependent QFLS (Suns-QFLS). Following Caprioglio et al.,[44] an internal ideality factor, which is dominated by the perovskite bulk and mostly unaffected by interfacial losses, can be derived. As shown in Figure 5B, the internal ideality of the perovskite sub-cell increases slightly from 1.39 to 1.41. This again highlights the minimal impact of radiation-induced defects on the perovskite bulk properties.

To generate an overall picture, we compare insights derived on perovskite/CIGS tandems to the perovskite/SHJ case, which we show in Figure 5C (see similar analyses to produce these values in Figures S5–S7). Similar to the perovskite on CIGS, the mean QFLS of the perovskite on SHJ remains high and reduces only slightly from 1.127 $\pm$ 0.013 eV (non-irradiated) to 1.109 $\pm$ 0.016 eV (irradiated). The QFLS reduction of ΔQFLS = 0.02 eV is an order of magnitude smaller than the V_{OC} loss observed in the perovskite/SHJ tandem of ΔV_{OC} = 0.31 V, clearly showing that the perovskite/SHJ tandem is limited by the SHJ bottom cell after irradiation. Surprisingly, we observe only minor changes in the ideality factor of the perovskite/SHJ tandem solar cell with irradiation (Figure 5D). In as-prepared devices, we estimate $\sum_i n_i$ = 2.55 for the perovskite/SHJ tandem and n_{SHJ} = 0.95 for an identically prepared SHJ single junction, the latter being due to Auger recombination typically dominating recombination in silicon cells.[45] The value remains <1 after irradiation (n_{SHJ} = 0.93), indicating that Auger processes still dominate the recombination mechanisms. In the perovskite/SHJ tandem, we estimate $\sum_i n_i$ = 2.50, which is close to the value before irradiation, and once again corroborates the radiation hardness of the perovskite top cell. This conclusion is further supported by Suns-QFLS measurements of the perovskite sub-cell that indicate an unchanged internal ideality factor of 1.39 (non-irradiated) and 1.41 (irradiated).

DISCUSSION

Taking both the electrical and optical characterization results into account, it is clear that proton-irradiation-induced trap states are formed within the CIGS bottom cell. Consequently, the CIGS bottom cell features a reduced V_{OC} and FF, which leads to reduced performance of the overall tandem. From the FF and V_{OC} losses of the CIGS single junction, we estimate that this constitutes ~60% of the performance loss observed in the perovskite/CIGS tandem solar cell after proton irradiation. Thus, the radiation hardness could be improved further by utilizing a more radiation-hard bottom cell, such as $Cu_2ZnSn(S,Se)_4$ (CZTSSe). Recent experiments revealed a promising radiation hardness for CZTSSe outperforming CIGS absorbers by a factor of two, albeit with their power-conversion efficiency still being a factor of two smaller than those of typical CIGS single-junction devices.[10] Other promising candidates are low-band-gap perovskite cells based on Sn-Pb mixtures[46–48]; however, their radiation hardness has not been investigated yet.

We have further established an additional recombination pathway that impacts FF and constitutes ~40% of the performance loss of the entire perovskite/CIGS tandem

CellPress
OPEN ACCESS

solar cell. Our optical and electrical measurements on the perovskite/SHJ tandem solar cell, as well as previously published perovskite single-junction results,[13] allow us to exclude degradation of the perovskite absorber layer itself. The unaffected values for QFLS and internal ideality factor of the perovskite sub-cell in the perovskite/CIGS tandem further suggest that the perovskite bulk is largely unaffected while pointing toward increased interfacial recombination. In contrast to perovskite/SHJ and perovskite single-junction solar cells, perovskite/CIGS tandem solar cells, require a 10-nm thick NiO layer conformally grown by plasma-assisted atomic layer deposition (ALD) between ZnO and PTAA to mitigate shunting of the perovskite top cell on the rough CIGS bottom cell.[20] Proton, γ- and UV-irradiation is known to induce defects in NiO that increase its conductivity while also leading to conversion from p-type to n-type behavior.[49–51] This deteriorates the energetic alignment between PTAA and ALD NiO, leading to less efficient extraction of charge carriers, thereby affecting FF and maximum power point (MPP) of the perovskite top cell. The luminescence of perovskite absorbers sandwiched between two selective contacts is known to be heavily influenced by surface recombination and charge carrier extraction into the selective contacts.[40,52] Changes in energetic alignment of PTAA/NiO will hence simultaneously impact charge extraction & luminescence properties explaining the observed PL enhancements and TRPL prolongations of the perovskite sub-cell (reduced PL quenching) in the perovskite/CIGS tandem after proton irradiation (see Figures 3 and 4); in perovskite/SHJ tandem solar cells that do not require an ALD NiO interlayer, we observe a slight reduction of the PL and shortening of the TRPL lifetime of the perovskite sub-cell (Figures S7 and S8). We note that the problematic NiO layer, as well as the degradation in the CIGS and SHJ bottom cells, would have been overlooked using low-energy proton irradiation ($E < 1$ MeV) that only impinges the topmost layers.

All in all, the perovskite/CIGS tandems possess a high radiation hardness and retain over ~85% of their initial performance even after 68 MeV proton irradiation and a dose of $\Phi = 2 \times 10^{12}$ p^+/cm^2, which is comparable to conventional GaInP/GaAs/Ge absorbers that retain ~82% of their initial performance at an identical displacement damage.[53] These irradiation conditions correspond to more than 50 years in space at the ISS orbit, and consequently, perovskite-based multijunction PV has the potential to become a disruptive space PV technology. Assuming further improvements in power-conversion efficiency approaching $\eta = 30\%$, the specific-power of perovskite/CIGS would be increased to 14 W/g (4 W/g if assuming a 25 μm thick substrate and encapsulation foil). Both values vastly exceed those of conventional used GaInP/GaAs/Ge absorbers at 0.8 W/g.[5] Perovskite/CIGS tandem solar cells are currently optimized for terrestrial PVs and have shown rapid progress,[20,22] with recent demonstration of flexible perovskite/CIGS tandem solar cells,[54] thereby rendering the above outlook achievable. Promising results by Barbé et al.[55] and Brown et al.[56] further suggest that perovskite single-junction devices can tolerate extreme temperature changes and operate well under low-intensity and low-temperature environments, both of which can be found in some space environments, albeit this needs to be verified for perovskite/CIGS tandems and their more complex layer stack. Thermo-mechanical stress from temperature cycling, diurnal cycles, and/or partial shading can cause additional degradation pathways, conditions that are equally present in space, high-altitude, and terrestrial environments. We therefore encourage dedicated investigations of emerging and established PV technologies using our described operando methodology to decouple degradation of the individual sub-cells by sequentially forcing one sub-cell into limitation conditions.

Conclusions

In summary, we have evaluated perovskite/CIGS and perovskite/SHJ tandem solar cells for their suitability to withstand the harsh radiation environment in space using tailored *in-operando* and *ex-situ* measurements during and after high-energetic proton irradiation. Our results show that perovskite/SHJ tandem solar cells degrade severely to 1% of their initial efficiency while perovskite/CIGS tandem solar cells retain over 85% of their initial efficiency under AM0 solar illumination even after 68 MeV proton irradiation at a dose of 2×10^{12} p^+/cm^2. Using high spatial resolution photoluminescence microscopy, we further showed that the open-circuit voltage potential of the perovskite top cell is unaffected after high-dose proton irradiation. Combining insights from selectively probed photoluminescence and intensity-dependent V_{OC} measurements, we isolated the layers responsible for the efficiency losses of the tandem solar cells. We find that the losses primarily arise from increased recombination in the CIGS bottom cell, and the atomic layer deposited nickel-oxide-based recombination contact. With a radiation hardness that rivals state-of-the-art III-V semiconductor-based space PV, our work identifies perovskite/CIGS tandem solar cells that can be processed on flexible foils, as a cheap, readily stowable and ultra-lightweight space PV technology with power-to-weight and power-to-cost ratios surpassing those of state-of-the-art III-V semiconductor-based triple- and quadruple-junction absorbers. While our proton irradiation mimics the damage in space and high-altitude environments, our insights and *in operando* methodology provide a new perspective to improve and investigate the long-term stability of emerging tandem solar cell technologies for terrestrial, high-altitude, and space applications.

EXPERIMENTAL PROCEDURES

Full details of experimental procedures can be found in the Supplemental Information.

SUPPLEMENTAL INFORMATION

Supplemental Information can be found online at https://doi.org/10.1016/j.joule.2020.03.006.

ACKNOWLEDGMENTS

F.L. acknowledges financial support from the Alexander Von Humboldt Foundation via the Feodor Lynen program and thanks Prof. Sir R. Friend for supporting his Fellowship at the Cavendish Laboratory. This work was supported by the European Research Council (ERC) under the European Union's Horizon 2020 research and innovation programme (HYPERION, grant agreement number 756962). M.J., A.A.A., E.K., and S.A. acknowledge financial support from the German Federal Ministry of Education and Research (BMBF) via program "Materialforschung für die Energiewende" (grant no. 03SF0540), by the German Federal Ministry for Economic Affairs and Energy (BMWi) through the 'PersiST' project (grant no. 0324037C). T.B. and C.A.K. acknowledge funding by BMWi through the speed-CIGS (grant no. 0324095E). D.K. and M.C. acknowledge financial support from the Dutch Ministry of Economic Affairs, via the Top-consortia Knowledge and Innovation (TKI) Program "Photovoltaic modules based on a p-i-n stack, manufactured on a roll-to-roll line featuring high efficiency, stability and strong market perspective" (PVPRESS) (TEUE118010) and "Bridging the voltage gap" (BRIGHT) (1721101). K.F. acknowledges the George and Lilian Schiff Fund, the Engineering and Physical Sciences Research Council (EPSRC), the Winton Sustainability Fellowship, and the Cambridge Trust for funding. S.D.S. acknowledges the Royal Society

CellPress
OPEN ACCESS

and Tata Group (UF150033). The authors acknowledge the EPSRC for funding (EP/R023980/1). E.M.T. has received funding from the European Union's Horizon 2020 research and innovation program under the Marie Skłodowska-Curie grant agreement no. 841265. A.R.B. acknowledges funding from Winton Studentship, Oppenheimer Studentship, and Engineering and Physical Sciences Research Council (EPSRC) Doctoral Training Centre in Photovoltaics (CDT-PV). K.G. acknowledges the Polish Ministry of Science and Higher Education within the Mobilnosc Plus program (grant no. 1603/MOB/V/2017/0).

AUTHOR CONTRIBUTIONS

F.L. initiated the research and planned the experiments with input from H.-C.N., B.R., N.H.N., and S.D.S.; M.J., E.K., and A.A.A. prepared and optimized the perovskite/CIGS and perovskite/SHJ tandem solar cells under guidance from S.A.; T.B. and A.B.M.-V. prepared and optimized the CIGS and SHJ bottom cells under guidance from C.A.K. and B.S., respectively. D.K. deposited the NiO by ALD under guidance from M.C.; F.L., J.B., and A.D. performed the proton irradiation experiments and recorded the *operando* data using a tailored illumination and measurement setup developed by F.L.; F.L. and M.J. performed the PV characterizations; F.L. and E.M.T. performed the hyperspectral PL measurements; K.F. calculated the QFLS maps; A.R.B. and F.L. recorded the CIGS PL under IR excitation; J.R. and F.L. recorded the SHJ PL; F.L. and K.G. recorded PL lifetime maps of the perovskite sub-cell. F.L. simulated the energy loss using SRIM. F.L. analyzed all data and took the lead in drafting the manuscript; F.L., M.J., H.-C.N., and S.D.S. wrote the paper with input from other authors. All authors contributed to the discussion of the results.

DECLARATION OF INTERESTS

S.D.S. is a co-founder of Swift Solar, Inc., a company commercializing high-power, lightweight perovskite solar panels.

Received: December 27, 2019
Revised: February 13, 2020
Accepted: March 6, 2020
Published: April 6, 2020

REFERENCES

1. Iles, P. (2001). Evolution of space solar cells. Sol. Energy Mater. Sol. Cells *68*, 1–13.

2. Raja Reddy, M. (2003). Space solar cells—tradeoff analysis. Sol. Energy Mater. Sol. Cells *77*, 175–208.

3. Sweeting, M.N. (2018). Modern small satellites-changing the economics of space. Proc. IEEE *106*, 343–361.

4. Genta, G. (2014). Private space exploration: a new way for starting a spacefaring society? Acta Astronaut *104*, 480–486.

5. Azur Space Solar Power GmbH (2019). http://www.azurspace.com/index.php/en/.

6. Spectrolab (2019). https://www.spectrolab.com/index.html.

7. Garcia, I., France, R.M., Geisz, J.F., McMahon, W.E., Steiner, M.A., Johnston, S., and Friedman, D.J. (2016). Metamorphic III-V solar cells: recent progress and potential. IEEE J. Photovoltaics *6*, 366–373.

8. Imaizumi, M., Sumita, T., Kawakita, S., Aoyama, K., Anzawa, O., Aburaya, T., Hisamatsu, T., and Matsuda, S. (2005). Results of flight demonstration of terrestrial solar cells in space. Prog. Photovoltaics Res. Appl. *13*, 93–102.

9. Yamaguchi, M. (2001). Radiation-resistant solar cells for space use. Sol. Energy Mater. Sol. Cells *68*, 31–53.

10. Suvanam, S.S., Larsen, J., Ross, N., Kosyak, V., Hallén, A., and Björkman, C.P. (2018). Extreme radiation hard thin film CZTSSe solar cell. Sol. Energy Mater. Sol. Cells *185*, 16–20.

11. Lang, F., Nickel, N.H., Bundesmann, J., Seidel, S., Denker, A., Albrecht, S., Brus, V.V., Rappich, J., Rech, B., Landi, G., and Neitzert, H.C. (2016). Radiation hardness and self-healing of perovskite solar cells. Adv. Mater. *28*, 8726–8731.

12. Miyazawa, Y., Ikegami, M., Chen, H.-W., Ohshima, T., Imaizumi, M., Hirose, K., and Miyasaka, T. (2018). Tolerance of perovskite solar cell to high-energy particle irradiations in space environment. iScience *2*, 148–155.

13. Lang, F., Jošt, M., Bundesmann, J., Denker, A., Albrecht, S., Landi, G., Neitzert, H.-C.C., Rappich, J., and Nickel, N.H. (2019). Efficient minority carrier detrapping mediating the radiation hardness of triple-cation perovskite solar cells under proton irradiation. Energy Environ. Sci. *12*, 1634–1647.

14. Cardinaletti, I., Vangerven, T., Nagels, S., Cornelissen, R., Schreurs, D., Hruby, J., Vodnik, J., Devisscher, D., Kesters, J., D'Haen, J., et al. (2018). Organic and perovskite solar cells for space applications. Sol. Energy Mater. Sol. Cells *182*, 121–127.

15. Sahli, F., Werner, J., Kamino, B.A., Bräuninger, M., Monnard, R., Paviet-Salomon, B., Barraud, L., Ding, L., Diaz Leon, J.J., Sacchetto, D., et al.

(2018). Fully textured monolithic perovskite/silicon tandem solar cells with 25.2% power conversion efficiency. Nat. Mater. *17*, 820–826.

16. Hou, F., Yan, L., Shi, B., Chen, J., Zhu, S., Ren, Q., An, S., Zhou, Z., Ren, H., Wei, C., et al. (2019). Monolithic perovskite/silicon-heterojunction tandem solar cells with open-circuit voltage of over 1.8 V. ACS Appl. Energy Mater. *2*, 243–249.

17. Albrecht, S., Saliba, M., Correa Baena, J.P., Lang, F., Kegelmann, L., Mews, M., Steier, L., Abate, A., Rappich, J.J.J., Korte, L., et al. (2016). Monolithic perovskite/silicon-heterojunction tandem solar cells processed at low temperature. Energy Environ. Sci. *9*, 81–88.

18. Jošt, M., Köhnen, E., Morales-Vilches, A.B., Lipovšek, B., Jäger, K., Macco, B., Al-Ashouri, A., Krč, J., Korte, L., Rech, B., et al. (2018). Textured interfaces in monolithic perovskite/silicon tandem solar cells: advanced light management for improved efficiency and energy yield. Energy Environ. Sci. *11*, 3511–3523.

19. Köhnen, E., Jošt, M., Morales-Vilches, A.B., Tockhorn, P., Al-Ashouri, A., Macco, B., Kegelmann, L., Korte, L., Rech, B., Schlatmann, R., et al. (2019). Highly efficient monolithic perovskite silicon tandem solar cells: analyzing the influence of current mismatch on device performance. Sustain. Energy Fuels *3*, 1995–2005.

20. Jošt, M., Bertram, T., Koushik, D., Marquez, J.A., Verheijen, M.A., Heinemann, M.D., Köhnen, E., Al-Ashouri, A., Braunger, S., Lang, F., et al. (2019). 21.6%-efficient monolithic perovskite/Cu(in,Ga)Se 2 tandem solar cells with thin conformal hole transport layers for integration on rough bottom cell surfaces. ACS Energy Lett *4*, 583–590.

21. Han, Q., Hsieh, Y.T., Meng, L., Wu, J.L., Sun, P., Yao, E.P., Chang, S.Y., Bae, S.H., Kato, T., Bermudez, V., and Yang, Y. (2018). High-performance perovskite/Cu(In,Ga)Se 2 monolithic tandem solar cells. Science *361*, 904–908.

22. Al-Ashouri, A., Magomedov, A., Roß, M., Jošt, M., Talaikis, M., Chistiakova, G., Bertram, T., Márquez, J.A., Köhnen, E., Kasparavičius, E., et al. (2019). Conformal monolayer contacts with lossless interfaces for perovskite single junction and monolithic tandem solar cells. Energy Environ. Sci. *12*, 3356–3369.

23. Oxford PV (2019). Oxford PV perovskite solar cell achieves 28% efficiency, Oxford PV, 20 December, 2018. https://www.oxfordpv.com/news/oxford-pv-perovskite-solar-cell-achieves-28-efficiency.

24. Daly, E.J., Drolshagen, G., Hilgers, A., and Evans, H.D.R. (1996). Space environment analysis: experience and trends. In Space environment analysis: Experience and trends, Environment Modelling for Space-based Applications, Symposium Proceedings (ESA SP-392), W. Burke and T.-.D. Guyenne, eds., pp. 15–22.

25. Miyazawa, Y., Ikegami, M., Miyasaka, T., Ohshima, T., Imaizumi, M., and Hirose, K. (2015). Evaluation of radiation tolerance of perovskite solar cell for use in space. In 2015 IEEE 42nd Photovoltaic Specialist Conference (PVS), pp. 1–4.

26. Barbé, J., Hughes, D., Wei, Z., Pockett, A., Lee, H.K.H., Heasman, K.C., Carnie, M.J., Watson, T.M., and Tsoi, W.C. (2019). Radiation hardness of perovskite solar cells based on aluminum-doped zinc oxide electrode under proton irradiation. Sol. RRL *3*, 1900219.

27. Otte, K., Makhova, L., Braun, A., and Konovalov, I. (2006). Flexible Cu(In,Ga)Se2 thin-film solar cells for space application. Thin Solid Films *511–512*, 613–622.

28. Ziegler, J.F., Ziegler, M.D.D., and Biersack, J.P.P. (2010). SRIM - the stopping and range of ions in matter (2010). Nucl. Instrum. Methods Phys. Res. B *268*, 1818–1823.

29. Street, R.A., Northrup, J.E., and Krusor, B.S. (2012). Radiation induced recombination centers in organic solar cells. Phys. Rev. B *85*, 205211.

30. Lang, F., Shargaieva, O., Brus, V.V., Neitzert, H.C., Rappich, J., and Nickel, N.H. (2018). Influence of radiation on the properties and the stability of hybrid perovskites. Adv. Mater. *30*, 29152795.

31. Messenger, S.R., Burke, E.A., Walters, R.J., Warner, J.H., Summers, G.P., and Morton, T.L. (2006). Effect of omnidirectional proton irradiation on shielded solar cells. IEEE Trans. Nucl. Sci. *53*, 3771–3778.

32. Walters, R.J., Warner, J.H., Messenger, S.R., Lorentzen, J.R., and Summers, G.P. (2006). On the need for low energy proton testing of space solar cells. In IEEE 4th World Conference on Photovoltaic Energy Conference (IEEE), *2006*IEEE 4th World Conference on Photovoltaic Energy Conference (IEEE), pp. 1899–1902.

33. Meusel, M., Baur, C., Létay, G., Bett, A.W., Warta, W., and Fernandez, E. (2003). Spectral response measurements of monolithic GaInP/Ga(In)as/Ge triple-junction solar cells: measurement artifacts and their explanation. Prog. Photovolt. Res. Appl. *11*, 499–514.

34. Hongliang, G., Yiyong, W., Jie, W., Bin, G., Jingdong, X., Qiang, S., and Hui, Y. (2018). External quantum efficiency artifacts in partial-irradiated GaInP/GaAs/Ge solar cells by protons and electrons. Energy Sci. Eng. *6*, 144–153.

35. Alurralde, M., Tamasi, M.J.L., Bruno, C.J., Martnez Bogado, M.G., Plá, J., Fernández Vázquez, J., Durán, J., Schuff, J., Burlon, A.A., and Stoliar, P. (2004). Experimental and theoretical radiation damage studies on crystalline silicon solar cells. Sol. Energy Mater. Sol. Cells *82*, 531–542.

36. Neitzert, H.-C., Ferrara, M., Kunst, M., Denker, A., Kertész, Z., Limata, B., Gialanella, L., and Romano, M. (2008). Electroluminescence efficiency degradation of crystalline silicon solar cells after irradiation with protons in the energy range between 0.8 MeV and 65 MeV. Phys. Stat. Sol. (b) *245*, 1877–1883.

37. Neitzert, H.C., Spinillo, P., Bellone, S., Licciardi, G.D., Tucci, M., Roca, F., Gialanella, L., and Romano, M. (2004). Investigation of the damage as induced by 1.7 MeV protons in an amorphous/crystalline silicon heterojunction solar cell. Sol. Energy Mater. Sol. Cells *83*, 435–446.

38. Hornbeck, J.A., and Haynes, J.R. (1955). Trapping of minority carriers in silicon. I. P-type silicon. Phys. Rev. *97*, 311–321.

39. Tennyson, E.M., Doherty, T.A.S., and Stranks, S.D. (2019). Heterogeneity at multiple length scales in halide perovskite semiconductors. Nat. Rev. Mater. *4*, 573–587.

40. Stolterfoht, M., Wolff, C.M., Márquez, J.A., Zhang, S., Hages, C.J., Rothhardt, D., Albrecht, S., Burn, P.L., Meredith, P., Unold, T., and Neher, D. (2018). Visualization and suppression of interfacial recombination for high-efficiency large-area pin perovskite solar cells. Nat. Energy *3*, 847–854.

41. Würfel, P. (1982). The chemical potential of radiation. J. Phys. C Solid State Phys. *15*, 3967–3985.

42. Katahara, J.K., and Hillhouse, H.W. (2014). Quasi-Fermi level splitting and sub-bandgap absorptivity from semiconductor photoluminescence. J. Appl. Phys. *116*, 173504.

43. Cowan, S.R., Roy, A., and Heeger, A.J. (2010). Recombination in polymer-fullerene bulk heterojunction solar cells. Phys. Rev. B *82*, 245207.

44. Caprioglio, P., Stolterfoht, M., Wolff, C.M., Unold, T., Rech, B., Albrecht, S., and Neher, D. (2019). On the relation between the open-circuit voltage and quasi-Fermi level splitting in efficient perovskite solar cells. Adv. Energy Mater. *9*, 1901631.

45. Green, M.A. (1984). Limits on the open-circuit voltage and efficiency of silicon solar cells imposed by intrinsic Auger processes. IEEE Trans. Electron Devices *31*, 671–678.

46. Tong, J., Song, Z., Kim, D.H., Chen, X., Chen, C., Palmstrom, A.F., Ndione, P.F., Reese, M.O., Dunfield, S.P., Reid, O.G., et al. (2019). Carrier lifetimes of >1 µs in Sn-Pb perovskites enable efficient all-perovskite tandem solar cells. Science *364*, 475–479.

47. Palmstrom, A.F., Eperon, G.E., Leijtens, T., Prasanna, R., Habisreutinger, S.N., Nemeth, W., Gaulding, E.A., Dunfield, S.P., Reese, M., Nanayakkara, S., et al. (2019). Enabling flexible all-perovskite tandem solar cells. Joule *3*, 2193–2204.

48. Lin, R., Xiao, K., Qin, Z., Han, Q., Zhang, C., Wei, M., Saidaminov, M.I., Gao, Y., Xu, J., Xiao, M., et al. (2019). Monolithic all-perovskite tandem solar cells with 24.8% efficiency exploiting comproportionation to suppress Sn(ii) oxidation in precursor ink. Nat. Energy *4*, 864–873.

49. Simnad, M.T., Smoluchowski, R., and Spilners, A. (1958). Effect of proton irradiation upon hydrogen reduction of NiO. J. Appl. Phys. *29*, 1630–1632.

50. Yamashina, T., Nagamatsuya, T., and Sano, M. (1968). Changes in catalytic activity and surface properties of nickel oxide with gamma-irradiation. Bull. Chem. Soc. Jpn. *41*, 2257–2263.

51. Gupta, P., Dutta, T., Mal, S., and Narayan, J. (2012). Controlled p -type to n -type conductivity transformation in NiO thin films by ultraviolet-laser irradiation. J. Appl. Phys. *111*, 013706.

52. Wolff, C.M., Zu, F., Paulke, A., Toro, L.P., Koch, N., and Neher, D. (2017). Reduced interface-mediated recombination for high open-circuit voltages in CH3NH3PbI3 solar cells. Adv. Mater. *29*, 1700159.

53. Messenger, S.R., Burke, E.A., Walters, R.J., Warner, J.H., and Summers, G.P. (2005). Using SRIM to calculate the relative damage coefficients for solar cells. Prog. Photovolt. Res. Appl. *13*, 115–123.

54. Fu, F., Nishiwaki, S., Werner, J., Feurer, T., Pisoni, S., Jeangros, Q., Buecheler, S., Ballif, C., and Tiwari, A.N. (2019). Flexible perovskite/Cu(In,Ga)Se2 monolithic tandem solar cells. arXiv. https://arxiv.org/abs/1907.10330v1.

55. Barbé, J., Pockett, A., Stoichkov, V., Hughes, D., Lee, H.K.H., Carnie, M., Watson, T., and Tsoi, W.C. (2020). In situ investigation of perovskite solar cells' efficiency and stability in a mimic stratospheric environment for high-altitude pseudo-satellites. J. Mater. Chem. C *8*, 1715–1721.

56. Brown, C.R., Eperon, G.E., Whiteside, V.R., and Sellers, I.R. (2019). Potential of high-stability perovskite solar cells for low-intensity–low-temperature (LILT) outer planetary space missions. ACS Appl. Energy Mater. *2*, 814–821.

JOUL, Volume *4*

Supplemental Information

Proton Radiation Hardness

of Perovskite Tandem Photovoltaics

Felix Lang, Marko Jošt, Kyle Frohna, Eike Köhnen, Amran Al-Ashouri, Alan R. Bowman, Tobias Bertram, Anna Belen Morales-Vilches, Dibyashree Koushik, Elizabeth M. Tennyson, Krzysztof Galkowski, Giovanni Landi, Mariadriana Creatore, Bernd Stannowski, Christian A. Kaufmann, Jürgen Bundesmann, Jörg Rappich, Bernd Rech, Andrea Denker, Steve Albrecht, Heinz-Christoph Neitzert, Norbert H. Nickel, and Samuel D. Stranks

Supplemental Data

Proton fluence at ISS orbit

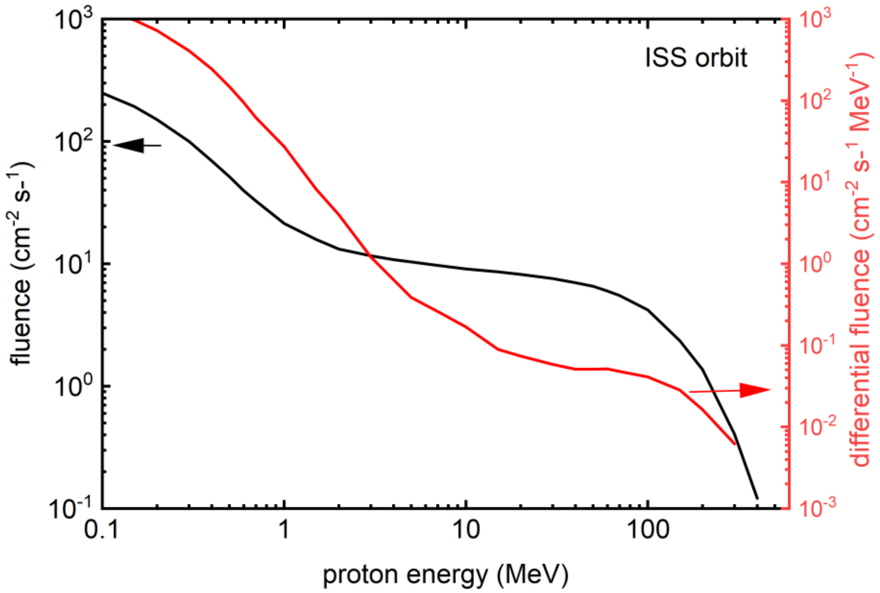

Figure S1: Proton spectrum in ISS orbit: Fluence and differential fluence of the proton spectrum in an ISS orbit as simulated using SPENVIS [18] This spectrum was used as input to simulate the damage from polyenergetic omnidirectional proton irradiation in the orbit of the ISS, as shown in Figure 1. See supplementary materials for computational details.

In-operando measurements after stopping the proton irradiation

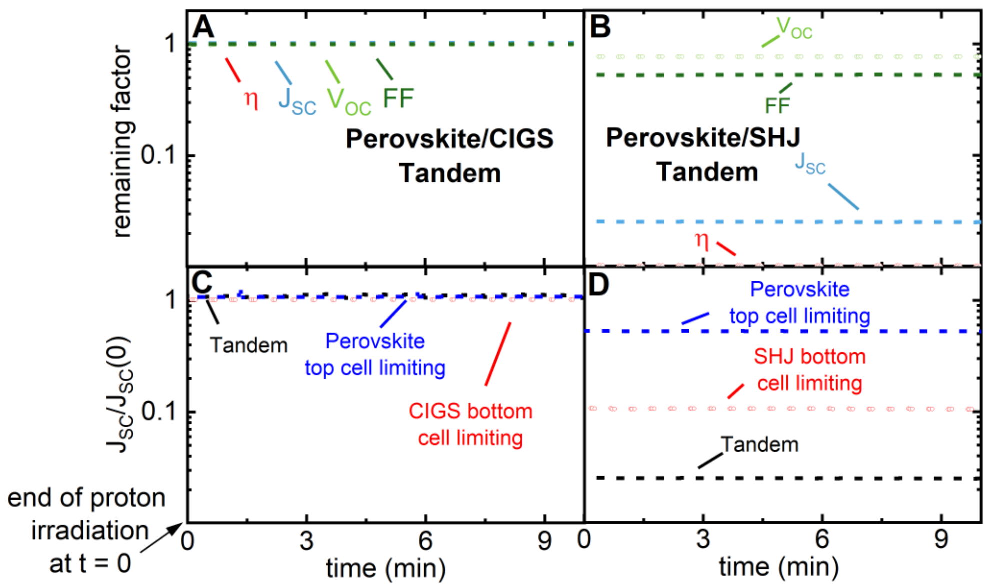

Figure S*2*: (A**,** B) *In-operando* measurements of V_{OC}, J_{SC}, FF, and η normalized to their initial value of the perovskite/CIGS and perovskite/SHJ tandem solar cells, related to Figure 1. Measurements were started directly after stopping the proton irradiation (after $\Phi = 2 \cdot 10^{12}$ p^{+}/cm^{2}). (C, D) Normalized short-circuit current of the perovskite/CIGS and perovskite/SHJ tandem solar cell under illumination with NIR and blue LED's at a wavelength of λ = 450 nm/850 nm alternatingly set to either 100 % or 14/5 %, again directly after stopping the proton irradiation. The employed illumination regimes are described in the main manuscript in more detail. (Fig 1G)

Operando Perovskite/SHJ JV characteristics

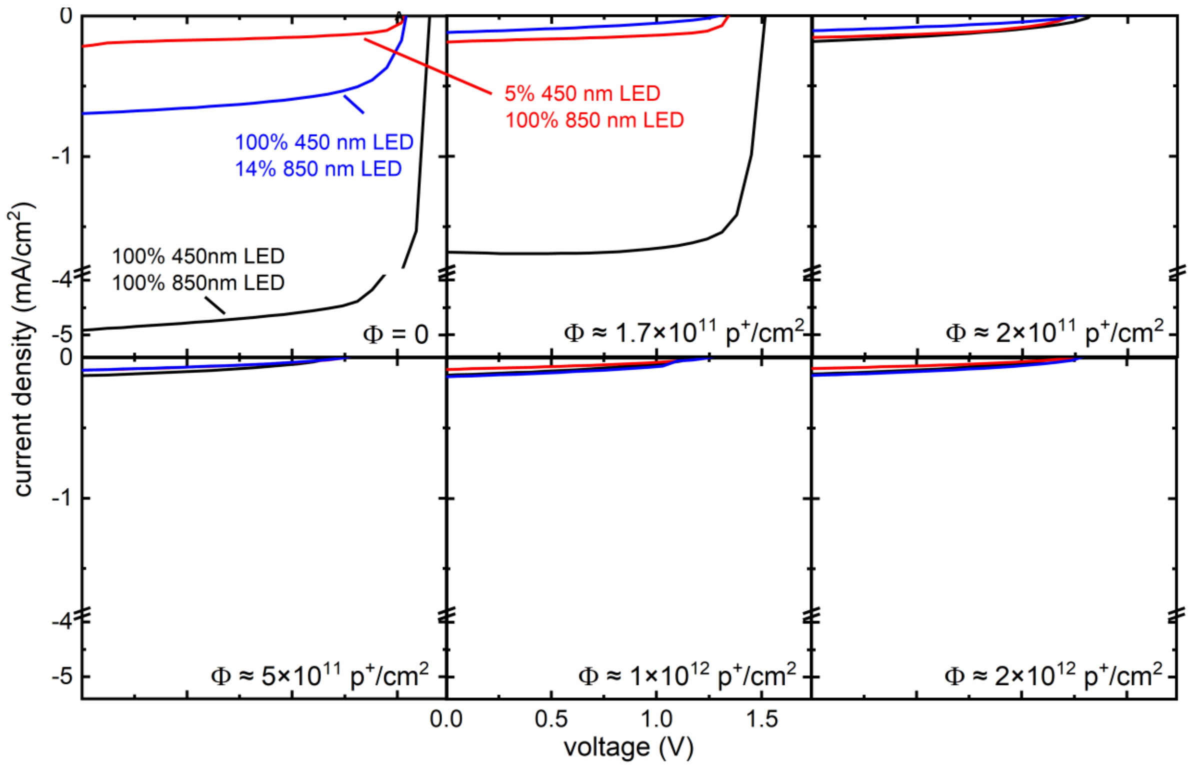

Figure S3: *Operando* JV characteristics under 450 and 850 nm LED illumination set to $I^{450nm} = 100\%$ & $I^{850nm} = 100\%$ (black), $I^{450nm} = 100\%$ & $I^{850nm} = 5\%$ (blue), $I^{450nm} = 14\%$ & $I^{850nm} = 100\%$ (red) of the perovskite/SHJ tandem solar cells under 68 MeV proton irradiation for accumulated doses of $\Phi \approx 0$, 1.7×10^{11}, 2×10^{11}, 5×10^{11}, 1×10^{12}, and 2×10^{12} p^+/cm^2., related to Figure 1.

Operando Perovskite/CIGS JV characteristics

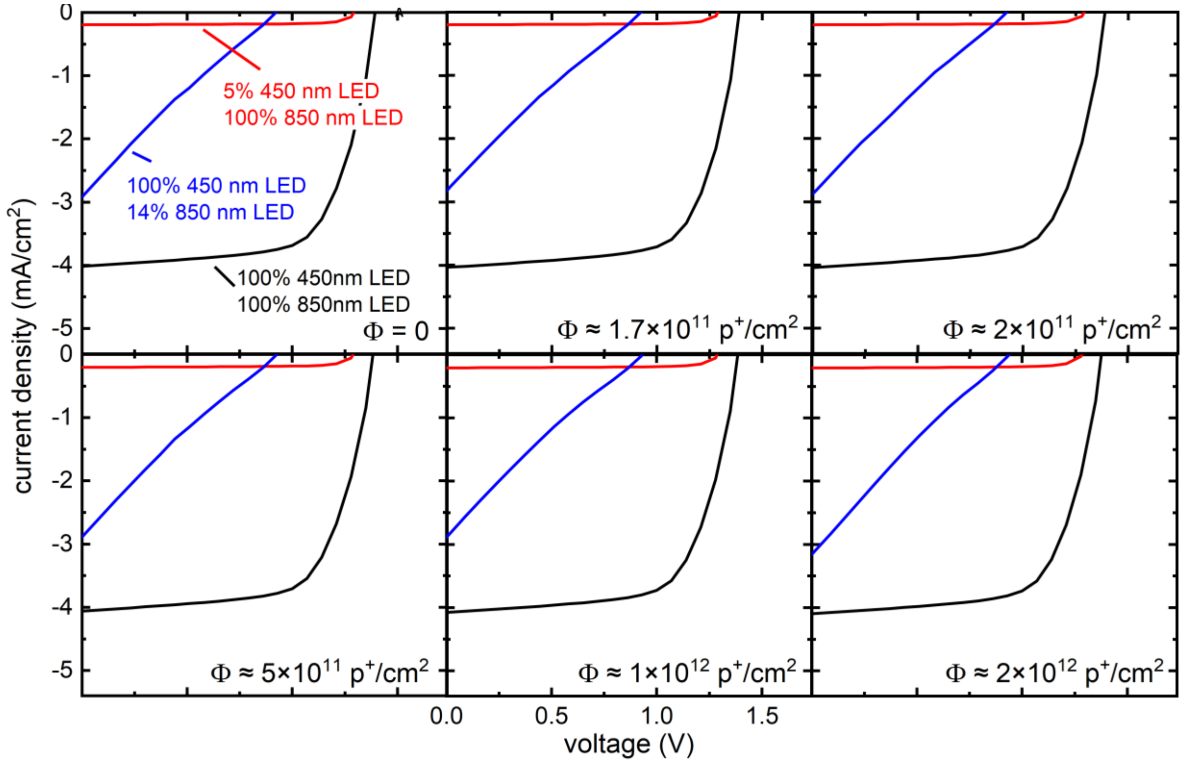

Figure S4: *Operando* JV characteristics under 450 and 850 nm LED illumination set to I^{450nm} = 100% & I^{850nm} = 100% (black), I^{450nm} = 100% & I^{850nm} = 5% (blue), I^{450nm} = 14% & I^{850nm} = 100% (red) of the perovskite/CIGS tandem solar cells under 68 MeV proton irradiation for accumulated doses of $\Phi \approx 0$, 1.7×10^{11}, 2×10^{11}, 5×10^{11}, 1×10^{12}, and 2×10^{12} p^+/cm^2, related to Figure 1.

Interestingly, the perovskite/SHJ and perovskite/CIGS tandem solar cells behave quite differently when the bottom cell is limiting the overall current (under illumination with 14% 850nm, 100% 450nm). We believe that this is caused by the lower shunt resistance of the CIGS bottom cell compared to the SHJ bottom cell. Evidence for this is found in Figure S10 and S19, where we provide the dark JV characteristics and derive a 10x higher shunt resistance in the case of SHJ bottom cells. A similar situation is often seen in commercial InGaP/GaAs/Ge triple-junction solar cells, where the InGaP and Ge sub-cells exhibit JV characteristics that are similar to the CIGS case here when forced into limitation by appropriate light biasing. Often this then complicates spectral response measurements. [19]

Summarized photovoltaic parameters

Table S1: Summarized photovoltaic parameters. In all cases, JV measurements were recorded from J_{SC}-to-V_{OC} and V_{OC}-to-J_{SC}. Given that the MPP tracking indicates similar results, we summarize parameters extracted from J_{SC}-to-V_{OC} scans only.

		V_{OC} (V)	J_{SC} mA/cm^2	FF %	η %	$η_{MPP}$ %	P_{MPP} W/m^2	J_{SC-EQE}^{top} mA/cm^2	J_{SC-EQE}^{bottom} mA/cm^2
**Perovskite/SHJ**									
as prep	AM1.5G	1.73	18.0	67.9	21.1	21.3	213	21.9	18.0
as prep	AM0	1.76	21.5	66.7	18.8	19.2	257	23.5	21.5
irradiated	AM1.5G	1.40	0.26	50.8	0.18	0.33	3.3	17.9	0.26
irradiated	AM0	1.41	0.3	52.8	0.16	0.31	4.0	22.0*	0.30
								*f = 4Hz	
**SHJ single**									
as prep	AM1.5G	0.74	36.3	77.9	20.9	-	-	36.3	
irradiated	AM1.5G	0.33	1.0	52.7	0.18	-	-	0.35	
**Perovskite/CIGS**									
as prep	AM1.5G	1.52	16.7	70.8	18.0	18.0	180	17.9	16.7
as prep	AM0	1.55	20.0	65.7	15.2	15.1	202	21.9	20.0
irradiated	AM1.5G	1.50	16.8	59.4	14.9	15.77	158	18.3	15.2/16.6*
irradiated	AM0	1.52	19.4	57.4	12.6	12.90	173	22.5	18.1/19.9*
									*f = 144Hz
**CIGS single**									
as prep	AM1.5G	0.64	34.2	70.9	15.5	-	-	34.2	
irradiated	AM1.5G	0.61	34.5	65.4	13.8	-	-	34.3	

Proton Irradiation of Identically Prepared SHJ Single Junction Solar Cells

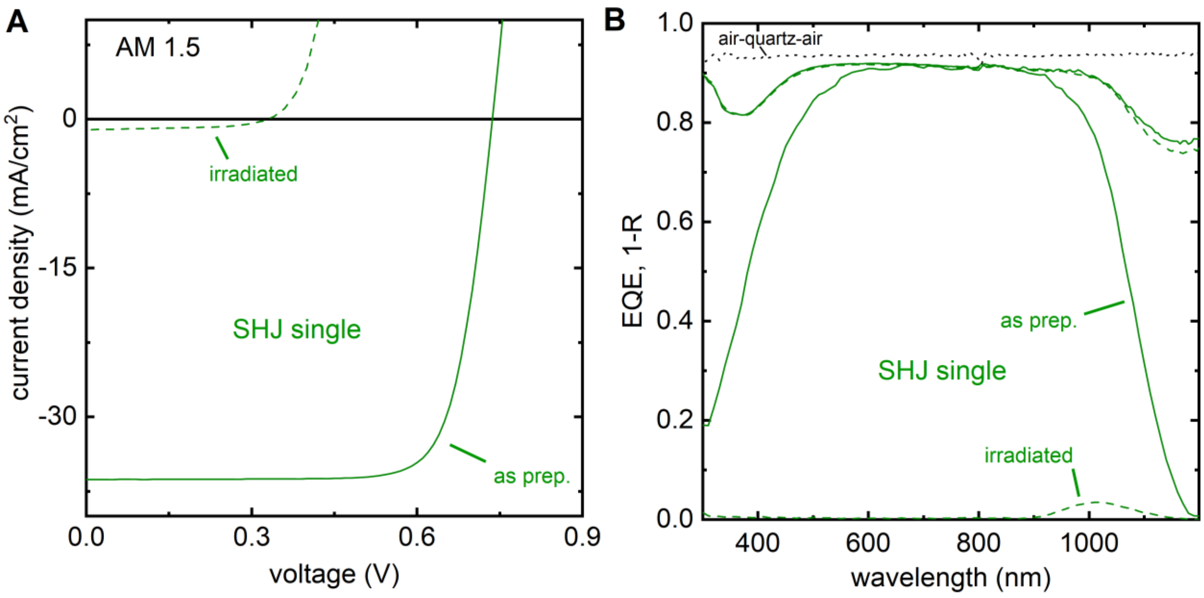

Figure S5: (A) Current-voltage characteristics of SHJ single junction solar cells under AM1.5G illumination conditions. Solid lines refer to measurements performed on as prepared solar cells, while dashed lines refer to measurements after 68 MeV proton irradiation at a total dose of $\Phi = 2 \cdot 10^{12}$ p^+/cm^2. (B) External quantum efficiency of the SHJ single junction solar cells before and after proton irradiation. The reflection is shown as 1-R. Related to Figures 2 and 5.

Perovskite/SHJ tandem solar cells

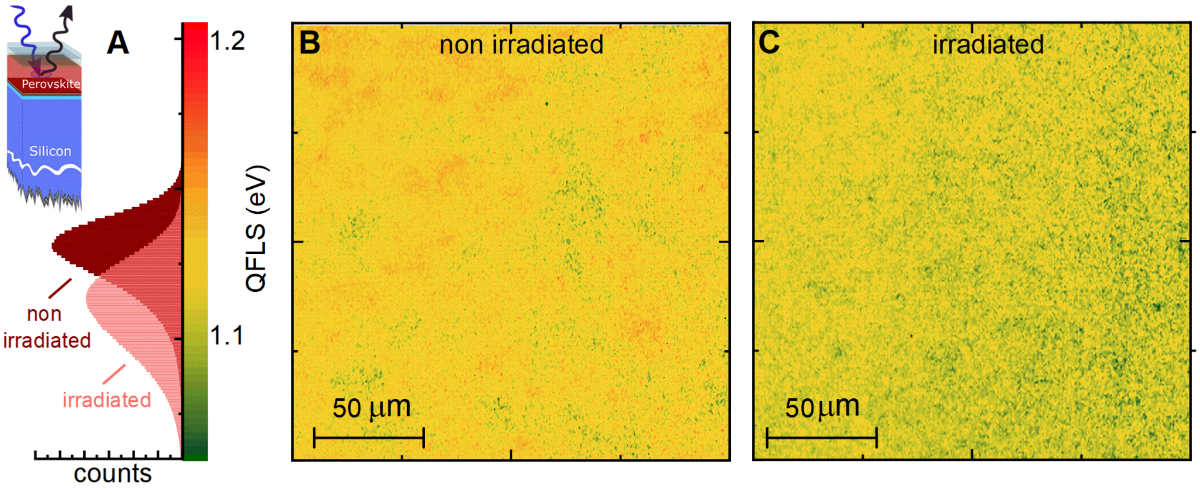

Figure S6: (A) Quasi-Fermi-Level-Splitting (QFLS) histogram and (B, C) QFLS maps of the perovskite top absorber in the as-prepared and proton-irradiated perovskite/CIGS tandem solar cell measured under 405 nm laser illumination with an intensity equivalent to 1 sun (see SM for details). Related to Figure 4.

Absolute photoluminescence imaging with high spatial resolution of the perovskite sub-cell in perovskite/SHJ tandem solar cells

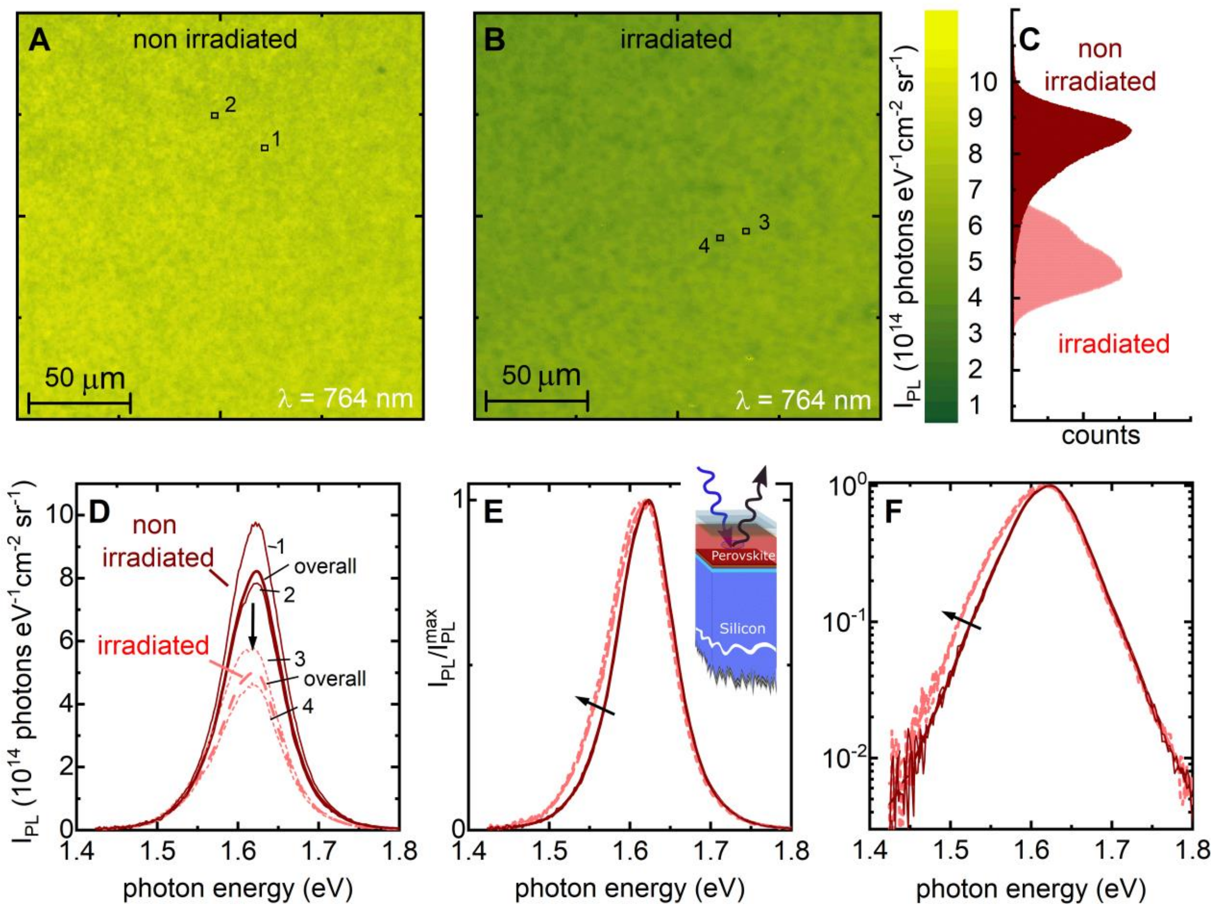

Figure S7: (A, B) Absolute photoluminescence images of the non-irradiated and irradiated (E_p = 68 MeV, $\Phi = 2 \cdot 10^{12}$ p^+/cm^2) perovskite sub-cell in the perovskite/SHJ tandem solar cell at a wavelength of λ = 764 nm. (C) Corresponding photoluminescence histogram. (D) Corresponding photoluminescence spectra. Data obtained on the non-irradiated and irradiated specimens are plotted as solid and dashed lines, respectively. Thick lines refer to the overall photoluminescence spectra, while thin lines refer to bright and dark regions, as indicated in (A, B) As described in the main text, the overall PL intensity reduces by a factor of two after proton irradiation. (E) and (F) depict the same data-set, now normalized, on a linear and semi-logarithmic scale. Evidently, both bright and dark regions feature a red-shifted shoulder after proton irradiation. Related to Figure 3.

Photoluminescence lifetime mapping with high spatial resolution of the perovskite/SHJ tandem solar cells

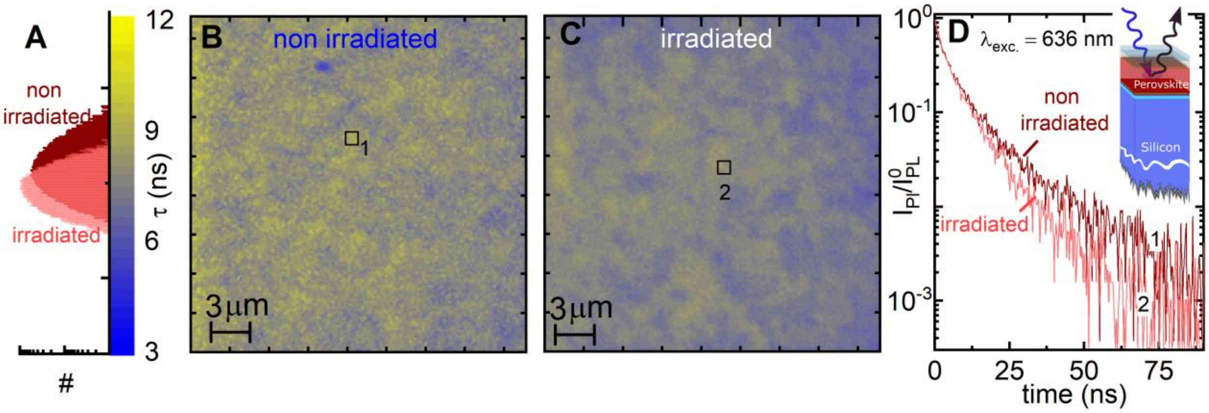

Figure S8: (A, B, C) Photoluminescence lifetime histogram and maps of the non-irradiated and irradiated (E_p = 68 MeV, Φ = 2·10^{12} p^+/cm^2) perovskite/SHJ tandem solar cells under excitation with a wavelength of λ_{exc} = 636 nm. Lifetimes were obtained by fitting the photoluminescence decay on each pixel with a bi-exponential decay. In general, slightly shorter lifetimes are observed after proton irradiation. Moreover, heterogeneity increases similar to the trend derived from absolute PL maps shown in Fig S3. (D) Selected TRPL decays of areas indicated in B and C. All measurements were performed on the complete device stack. Selective contacts are known to quench the PL decay in hybrid perovskites significantly, due to rapid charge carrier extraction into the contact layers and/or additional surface recombination at the interface between perovskite and the employed selective contacts. [20] Related to Figure 3.

Optical spectroscopy of the SHJ bottom cell

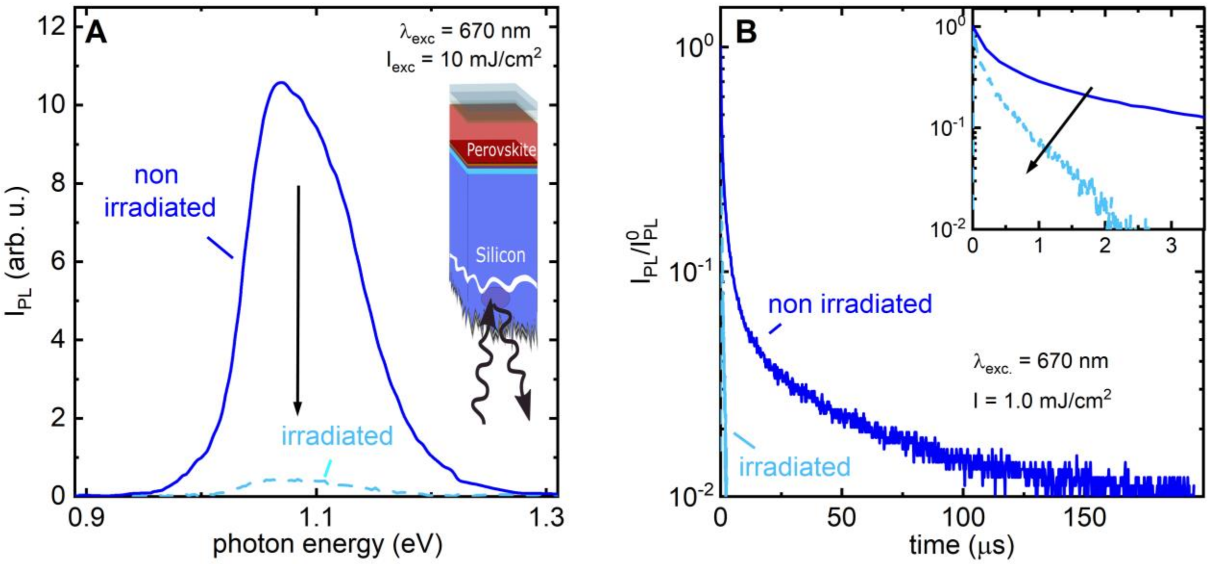

Figure S9: Photoluminescence spectra (A) and transient photoluminescence (TRPL) (B) of the SHJ bottom absorber before and after proton irradiation. In both cases, excitation was performed from the backside on regions that were not covered with Ag. Related to Figure 3.

Dark current-voltage characteristics of the perovskite/SHJ tandem solar cells

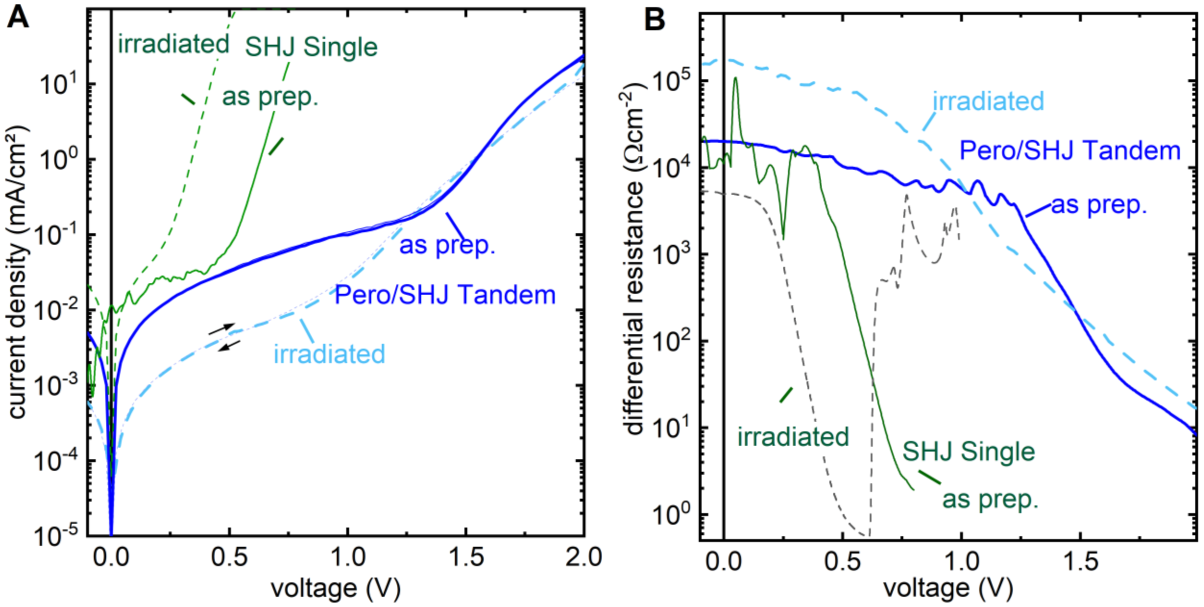

Figure S10: (A) Dark current-voltage and (B) differential-resistance-voltage characteristics of investigated Perovskite/SHJ Tandem and SHJ Single junction solar cells. Solid and dashed lines refer to as prepared and irradiated specimens, respectively. (E_p = 68 MeV, $\Phi = 2 \cdot 10^{12}$ p^+/cm^2) Interestingly, we observe an increase in rectification and differential resistance for the perovskite/SHJ tandem solar cells. Similar increases have been found for perovskite single junction solar cells.[21] SHJ single junction solar cells feature a substantial decrease of the built-in-voltage after irradiation that corroborates the V_{OC} losses after irradiation. Related to Figure 2.

Supplementary Note 1

To further assess the impact of the proton irradiation on the perovskite sub-cell, we selectively probed the perovskite top cell using steady-state and time-resolved photoluminescence (PL and TRPL) measurements. Similar to the perovskite/CIGS tandem solar cells, we employ absolute hyperspectral PL imaging with high spatial resolution to record the local QFLS of the perovskite sub-cell with 405 nm excitation generating equivalent current densities to 1 sun. In Figure S3, we show QFLS maps of an irradiated (E) and a non-irradiated device (D), along with the number histograms from the measured pixels (C). The mean QFLS remains high and reduces only slightly from 1.13 ± 0.01 eV (non-irradiated) to 1.11 ± 0.02 eV (irradiated). Similar to the perovskite/CIGS case, an increase in heterogeneity can be identified, as seen in the slightly negatively skewed histogram. Interestingly, we observe a low energy shoulder in PL emission throughout the entire specimen after irradiation, which indicates the presence of radiation-induced tail states. (Figure S7 E & F)) Concomitant TRPL maps reveal an increase in heterogeneity as well as an overall decreased lifetime (Figure S8), consistent with the generation of radiation-induced tail states. Note that we do not observe similar changes of the perovskite PL when deposited on CIGS (Figure S14 E), which we attribute to radiation-induced changes of NiO that increases and prolongs the PL and TRPL of the perovskite sub-cell in the perovskite/CIGS tandem stack. (see the main manuscript for further details). To compare these subtle changes in perovskite sub-cell luminescence to the SHJ bottom-cell, we selectively probed the SHJ PL and TRPL in the tandem stack by excitation from the bottom. PL and TRPL data plotted in Figure S9B & C reveal large differences. Most importantly, the PL intensity is quenched by an order of magnitude after proton irradiation. Furthermore, the PL lifetime is drastically reduced after irradiation. Both effects indicate the presence of radiation-induced recombination centers in the SHJ bottom cell. This is corroborated by identically prepared and irradiated SHJ single junction devices, which show a vast reduction in J_{SC} and EQE from 36.3 mA/cm^2 and 92 % (max) to 1 mA/cm^2 and 3.4 % (max) after proton irradiation. (Note that the change in J_{SC} of 36.3 to 0.34 mA/cm^2 and 19.9 to 0.26 mA/cm^2 is larger for the SHJ single junction device compared to the SHJ bottom cell, resulting in a larger radiation-induced V_{OC} loss in SHJ single junctions compared to SHJ bottom cells)

Radiation-induced Si-vacancies (V_{Si}) and di-vacancies are known to diminish the minority carrier lifetime of crystalline silicon [22–28], and the associated gradual decrease in performance during proton irradiation has been studied extensively. [26,27,29–33] Abrupt degradation of the short-circuit current and performance of silicon solar cells during high-energy high-fluence proton irradiation has further been observed and explained by an exponential decrease in majority carrier concentration, which provokes an abrupt increase in base resistance. [23] The perovskite/SHJ tandem solar cell tested here relies - like the majority of perovskite/SHJ tandem solar cells to date [1,34–36] - on a phosphorous doped n-type float zone (FZ) Si base. Positron annihilation spectroscopy has recently shown that high energetic proton irradiation of n-type silicon forms thermally stable phosphorous-divacancy complexes that are majority carrier traps. [22]

This can de-activate the phosphorous doping and reduce the J_{SC} reduces rapidly. While dissociation of Si di-vacancies and phosphorous-divacancy complexes can restore the minority carrier lifetime and the majority carrier concentration, the required temperatures of 450 K and above would inevitably deteriorate the perovskite sub-cell with its organic contact layers. [23,25] Optimized Si space solar cells, often rely on a boron-doped p-type base and a very thin wafer thickness of 50 µm to withstand about 10^{14} p/cm^2 at 10 MeV. [23] Radiation- and light-induced boron-oxygen complexes, however, are efficient minority carrier traps and, hence, it will be crucial to use float zone silicon wafers with low oxygen content. [37,38] Using a p-type FZ silicon base for perovskite/SHJ tandem solar cells is not straight forward as the required regular perovskite top cell, often relies on high-temperature TiO_2 contact layers which would deteriorate employed hydrogenated amorphous silicon layers. [39] Further, inverted perovskite top cells, as used in this study, are often found to be more reliable and robust against UV, oxygen, and moisture-induced degradation. [40] Using a thin silicon base, ultimately, will require advanced light management strategies to ensure the efficient collection of NIR light. [41]

The most promising measures to strengthen the radiation hardness of perovskite/SHJ tandem solar cells, therefore, comprise an increased perovskite bandgap (forcing the tandem into limitation by the more radiation hard perovskite, thereby masking radiation-induced degradation of the SHJ bottom cell) and/or changes of the SHJ architecture (to become more robust to the inevitable radiation-induced lifetime loss, i.e., by employing a thinner silicon base). Both measures will, however, sacrifice some of the excellent initial performance for improved radiation hardness. Further, the vast and inherent impact of radiation-induced defects on the electrical properties of crystalline silicon makes such approaches challenging and ultimately more expensive.

Proton Irradiation of Identically Prepared CIGS Single Junctions Solar Cells

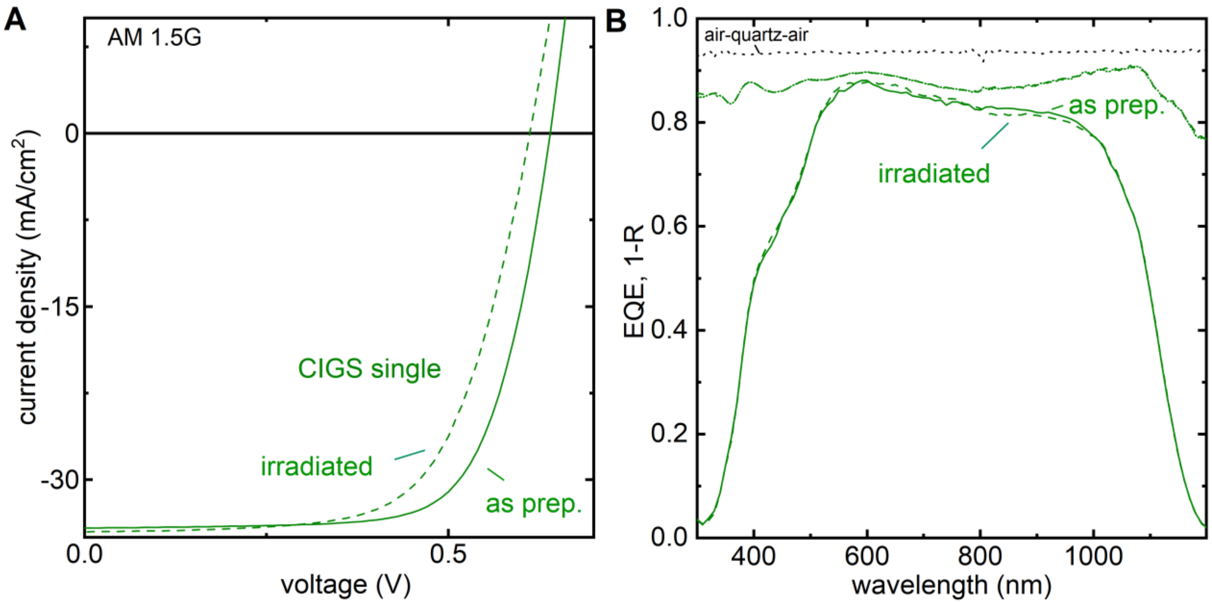

Figure S11: (A) Current-voltage characteristics of CIGS single junction solar cells under AM1.5G illumination conditions. Solid lines refer to measurements performed on as prepared solar cells, while dashed lines refer to measurements after 68 MeV proton irradiation at a total dose of $\Phi = 2 \cdot 10^{12}$ p^+/cm^2. (B) External quantum efficiency of the CIGS single junction solar cells before and after proton irradiation. The reflection is shown as 1-R. Related to Figure 2.

Dark current-voltage characteristics for perovskite/CIGS tandem solar cells

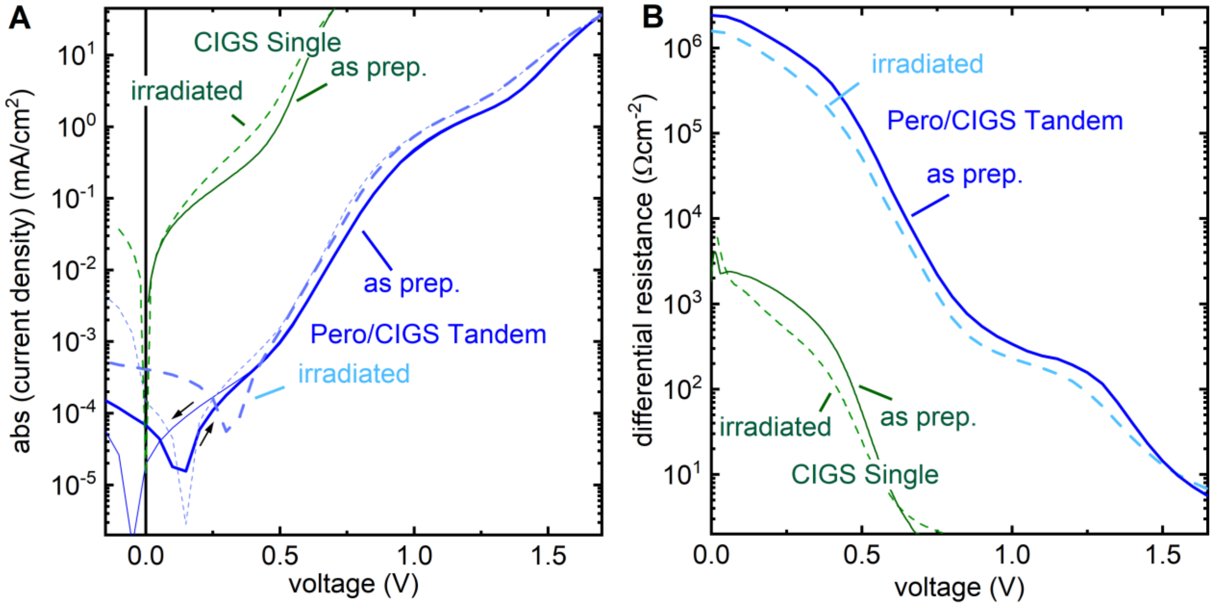

Figure S12: (A) Dark current-voltage and (B) differential resistance-voltage characteristics of investigated perovskite/CIGS tandem and CIGS single junction solar cells. Solid and dashed lines refer to as prepared and irradiated specimens, respectively. (E_p = 68 MeV, $\Phi = 2{\cdot}10^{12}$ p^+/cm^2) In contrast to the perovskite/SHJ tandem solar cell no increase in rectification and differential resistance visible. Interestingly, the perovskite/CIGS tandem solar cell features some hysteresis, which is visible only in the dark JV curves and not under AM0 or AM1.5G illumination. Related to Figure 2.

Photoluminescence lifetime mapping with high spatial resolution of the perovskite sub-cell in the perovskite/CIGS tandem solar cell

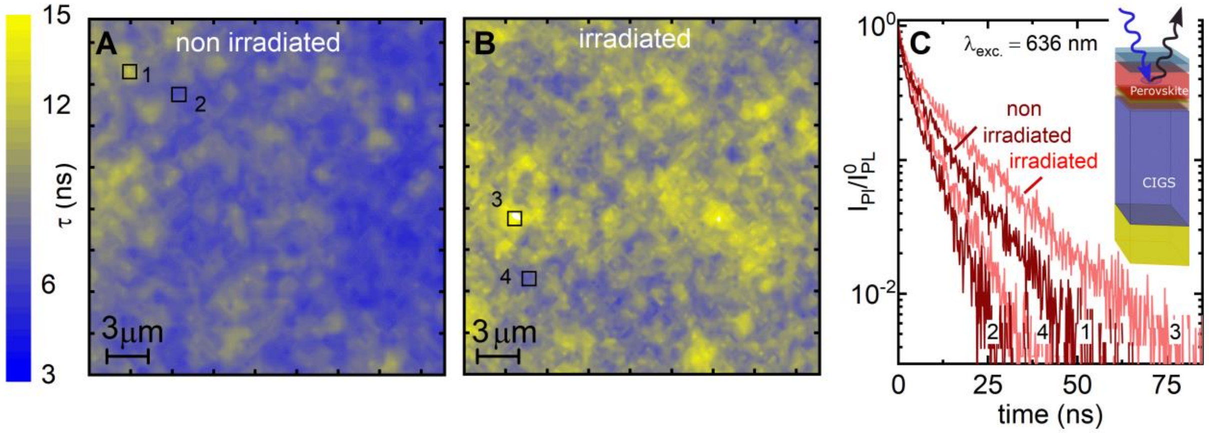

Figure S13: (A, B) Photoluminescence lifetime mapping of the non-irradiated and irradiated (E_p = 68 MeV, $\Phi = 2 \cdot 10^{12}$ p^+/cm^2) perovskite sub-cell in the perovskite/CIGS tandem solar cell under excitation with a wavelength of $\lambda_{ex.}$ = 636 nm, based on a single exponential fitting of the photoluminescence decay on each pixel. (C) Photoluminescence decay of selected short and long lifetime regions on the non-irradiated and irradiated specimens. The PL lifetime maps show an increased heterogeneity and more long-lifetime areas after irradiation. Overall this results in a longer lifetime. Related to Figure 4.

Absolute photoluminescence imaging with high spatial resolution of the perovskite sub-cell in the perovskite/CIGS tandem solar cell

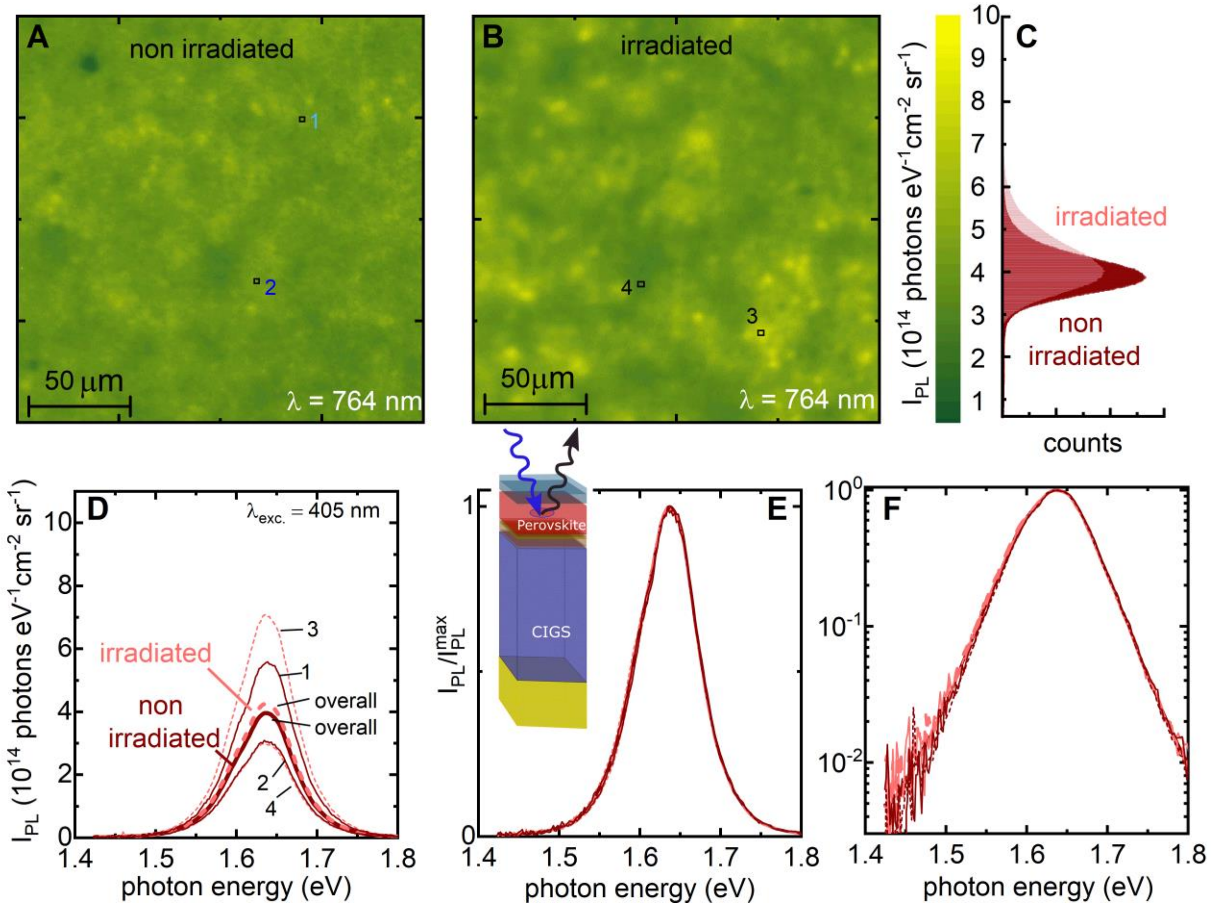

Figure S14: (A, B) Absolute photoluminescence images of non-irradiated and irradiated (E_p = 68 MeV, $\Phi = 2\cdot10^{12}$ p^+/cm^2) perovskite sub-cell in the perovskite/SHJ tandem solar cell at a wavelength of λ = 764 nm. **c** Corresponding photoluminescence histograms revealing an increase in heterogeneity. (D) Corresponding photoluminescence spectra. Data obtained on the non-irradiated and irradiated specimens are plotted as solid and dashed lines, respectively. Thick lines refer to the overall photoluminescence spectra while thin lines refer to bright and dark regions, as indicated in A Interestingly, bright regions feature a higher PL intensity after irradiation, while the PL intensity of dark regions remains unchanged. (E) and (F) depict the same data-set, now normalized and on a linear and semi-logarithmic scale. In contrast to the PL spectra of the perovskite/SHJ tandem, no low energy shoulder is observed. Related to Figure 4.

Spectral mismatch of mimicked AM0 and AM1.5G conditions

To estimate the spectral mismatch of the mimicked AM0 and AM1.5G spectra, we measured the irradiance as a function of wavelength and compare it in figure S11 to the ASTM G-173 and ASTM E-490 spectra. The upper panel further specifies the spectral mismatch in 100 nm intervals, calculated from the ratio of the incident irradiance integrated over the specified wavelength interval.

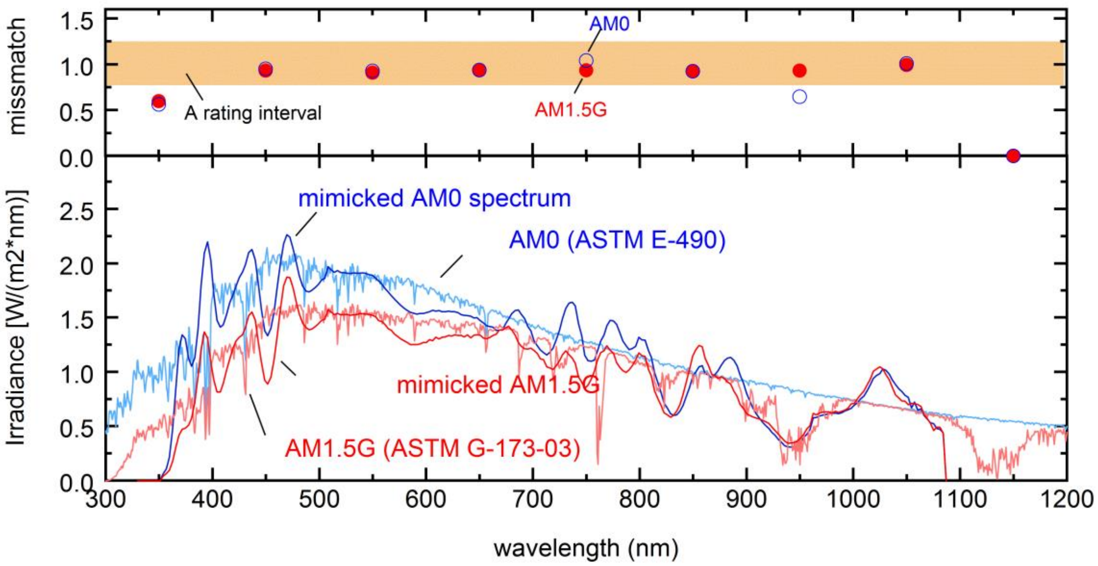

Figure S15: Mimicked AM 0 and AM 1.5G spectra, compared to the standard AM0 and AM1.5G spectra[42]. The top panel depicts the spectral mismatch in 100 nm intervals. Related to Figure 2.

Supplemental Experimental Procedures

Preparation of Perovskite/SHJ Tandem Solar Cells*:* Prepared perovskite/SHJ tandem solar cells comprise LiF/IZO/SnO_2/C_{60}/Perovskite/PTAA/ITO/(n^+) nc-SiOx/(i)a-Si:H/(n)-c-Si/(i)-a-Si:H/(p)-a-Si:H/Al:ZnO/Ag and feature an active area of 0.81 cm^2. A detailed description of the fabrication procedures is described elsewhere. [1] In here, we detail fabrication of the two absorber layers only: For the perovskite top absorber 1.5 M nominal PbI_2 and $PbBr_2$ in DMF:DMSO = 4:1 volume were first prepared as stock solutions and then added to FAI and MABr with 10 % PbX_2 excess, respectively (X = I or Br). The so obtained $FAPbI_3$ and $MAPbBr_3$ were then mixed in a 5:1 volume ratio to obtain a “double cation” perovskite. Finally, 5 % volume of 1.5 M nominal CsI in DMSO was added to form a “triple cation” perovskite and subsequently spun at 4000 rpm for 35 s. 25 s after the start of a spinning, 400 µl ethyl acetate anti-solvent drop was utilized, and finally, the films were annealed at 100°C for 1 h. For the SHJ bottom cell, a 260 µm thick polished FZ <100> n-type crystalline silicon (c-Si) wafer in a rear junction configuration was used. The front surface of the wafer was left polished to facilitate the perovskite top cell deposition, while the rear surface of the wafer was chemically textured to obtain random pyramids with <111> facets to improve the optical response of the bottom cell in the NIR region. Again, a more detailed description can be found elsewhere. [1] Fabricated Perovskite/SHJ tandem solar cells were then encapsulated under inert atmosphere using a quartz cover and epoxy resin. No intermediate index matching layers or light-management foils were used, as they typically darken upon proton irradiation due to the formation of radiation-induced color centers. As we found previously, such coloring can reduce the J_{SC} of perovskite solar cells dramatically and consequently dominate proton irradiation-induced degradation. [2]

Preparation of Perovskite/CIGS Tandem Solar Cells*:* Prepared perovskite/CIGS tandem solar cells comprise LiF/IZO/SnO_2/C_{60}/Perovskite/PTAA/NiO_x/ZnO/CdS/CIGS/Mo/glass and feature an active area of 0.81 cm^2. A detailed description of the involved preparation procedures is given elsewhere. [3] Here, we summarize the deposition of both absorber layers and the NiOx layer only: The perovskite absorber is prepared identically to the perovskite/SHJ tandem solar cells described above. For the CIGSe absorber, thermal evaporation from elemental sources using a processing sequence based on the well-known multi-stage process is used. [4] In this process, an $(In,Ga)_2Se_3$ precursor is deposited at a substrate temperature of 300 °C in the first stage, followed by the evaporation of Cu and Se at a substrate temperature of 530 °C until the absorber becomes overall copper-rich. The process is completed by the evaporation of In, Ga, and Se in the last stage to make the absorber copper poor again (CGI=0.9, GGI=0,3). The NiOx was deposited after a pre-deposition oxygen plasma treatment using atomic layer deposition from bismethylcyclopentadienyl-nickel ($Ni(MeCp)_2$), 97%, Sigma-Aldrich) as the precursor and an O_2 plasma as the co-reactant. A total of 350 ALD cycles were performed each consisting of 3 s $Ni(MeCp)_2$ dose, 4 s purge time , 3 s O_2 plasma exposure, and 1 s purge time. Details on the NiOx process development including the saturation curves are published elsewhere. [5] Similar to perovskite/SHJ tandem cell, the fabricated perovskite/CIGS tandem solar cells were then encapsulated under inert atmosphere using a quartz cover and epoxy resin.

Proton Irradiation*:* The tandetron-cyclotron combination of the Helmholtz-Zentrum Berlin[6,7] was used to accelerate protons to an energy of 68 ± 1 MeV. A thin scattering foil and appropriate aperture masks were used to provide a homogeneous irradiation over an area of 3.0 cm^2. The beam intensity was monitored online, utilizing a transmission ionization chamber. After irradiation, all devices were transferred to a lead cabinet until the activity of generated short-living isotopes in the irradiated samples dropped to a safe level of less than 10^3 Bq (~10 days of storage). To avoid the thermally-activated healing of radiation-induced defect states often observed in CIGS absorbers above 400 K, the devices were stored at RT in the dark. [8–10]

***In-operando* measurements**: *In-operando* measurements of the photovoltaic parameters were recorded during proton irradiation. Therefore, a Keithley 2400 was remotely controlled to record JV measurements from V_{OC}-to-J_{SC} every 3 seconds. This high repetition rate did not allow for reverse and forward measurements. Illumination was performed using two high-intensity LED's that are selectively absorbed in the high and low gap sub-cells (λ_{LED}^{1} = 450 nm and λ_{LED}^{2} = 850 nm). When set to 100%, both LED illuminated the entire active area homogenously at about ¼ AM0. Then the λ_{LED}^{1} = 450 nm LED was set to 0.7 mW/cm^2 (5 % of its initial value), thereby forcing the perovskite sub-cell to limit the overall current. Subsequently, the λ_{LED}^{2} = 850 nm LED was set to 1 mW/cm^2 (14% of its initial value), thereby forcing the SHJ or CIGS bottom cell to limit the overall current. The three used regimes were alternated every 10 s during proton irradiation using a homemade Arduino controlled circuit. Note, the low-intensity settings were adjusted to slightly different values to allow an unambiguous assignment of the employed regimes. Radiation-induced degradation of the employed LEDs and LED optics was avoided using focusing mirrors and keeping the LEDs at a safe distance from the proton beam.

Current-Voltage-Characteristics*:* AM1.5G and AM0 spectra were simulated using a Wavelabs Sinus 70 AAA LED sun simulator. The simulated and target spectra, as well as a mismatch calculation, are shown in Fig S11. The intensity was adjusted to 100 or 135 mW/cm² respectively by measuring the short-circuit current of a calibrated silicon solar cell (Fraunhofer ISE). Current-voltage scans were performed in forward and reverse direction with a voltage sweep of 85 mV/s. Additionally, we tracked the maximum power point (MPP) using homemade feedback software. In all cases, the temperature amounted to 25°C. In some cases, shadow masks (A = 0.778 cm^2) were used to avoid underestimation of the active area.

Suns-V_{OC} measurements: To estimate ideality factors, we varied the incident light intensity by modulating the LED intensity and measured the stabilized V_{OC}. In all cases, the temperature was set to 25°C using a temperature-controlled stage. In the case of single junction solar cells, the slope of the Suns-V_{OC} data can be used to estimate the ideality factor n using:

$$V_{OC,X} = \frac{k_B T}{q} \cdot n \cdot \ln(X) + V_{OC,X=1} \quad (1)$$

Here k_B is the Boltzmann constant, and T is the temperature. In the case of monolithic tandem solar cells, the V_{OC} equals the sum of the sub-cell open-circuit voltage $V_{OC,i}$. and hence, the intensity dependence of the V_{OC} can be approximated by:

$$V_{OC,X}^{tandem} = \frac{k_B T}{q} \cdot \sum_i n_i \cdot \ln(X) + V_{OC,X=1}^{tandem} \quad (2)$$

Here the n_i denotes the ideality factor of the individual sub-cell i. The ideality factor of a tandem solar cell n^{tandem} is thus given by the sum of the individual sub-cell ideality factors.

$$n^{tandem} = \sum_i n_i \quad (3)$$

An identical finding is obtained using the segmental approximation often employed to describe the current-voltage characteristics of multijunction solar cells, including InGaAs/GaAs and GaInP/GaInAs/Ge tandem solar cells. [11]

External quantum efficiencies (EQE): Appropriate light and voltage biases are required to record the spectral response of the perovskite/SHJ and perovskite/CIGS tandem cells. For this purpose, we employed LEDs at a wavelength of λ = 455 nm and λ = 855 nm that are selectively absorbed in the respective sub-cells. In case of the perovskite/SHJ tandem, the perovskite sub-cell was measured at an intensity of 2.3 mW/cm^2 (λ = 455 nm) and 2.9 mW/cm^2 (λ = 855 nm)) while the SHJ sub-cell was

measured using 14.5 mW/cm^2 (λ = 455 nm). In the case of the perovskite/CIGS tandem, an intensity of 6 mW/cm^2 (λ = 855 nm) and 16.4 mW/cm^2 (λ = 455 nm) was used for the perovskite and CIGS sub-cells, respectively. The CIGS bottom cell was also measured under bias light from a halogen lamp (109 mW/cm^2, 1.1sun (AM15) equivalent) equipped with a 750nm short pass filter. The EQE of both sub-cells was integrated over an AM1.5G or AM0 spectrum. Derived values were used to avoid overestimation of the J_{SC} measured under mimicked AM1.5G and AM0 illumination. Standardly, the monochromatic probe was chopped at 74 Hz. In the case of the perovskite/SHJ tandem solar cell, we additionally employed lower chopper frequencies as indicated. In the case of the perovskite/CIGS tandem solar cell, we further used higher chopper frequencies as indicated.

Absolute Photoluminescence imaging: Absolute photoluminescence maps were recorded using a hyperspectral widefield imager from Photon etc. at a magnification of 20x. Following literature [12], the setup was calibrated by combining a spectral calibration employing a calibrated halogen lamp that was coupled into an integrating sphere and an absolute calibration at one wavelength using a fiber-coupled laser. Excitation was performed using a 405 nm laser set to 1 sun (AM1.5G) equivalent fluence. The laser intensity was, therefore, adjusted to 2130 W/m^2, which corresponds to $4.3\cdot10^{21}$ photons m^{-2}s^{-1}. Taking into account the EQE at λ = 405 nm of 0.67 and 0.55 for the perovskite/Si and perovskite/CIGS tandem solar cells, respectively $n^{Pero/Si} = 2.92\cdot10^{21}$ m^{-2}s^{-1} and $n^{Pero/Si} = 2.37\cdot10^{21}$ m^{-2}s^{-1} charge carriers are generated. These values correspond well to the generation rate under AM1.5G of $n = \int_{300\,nm}^{800\,nm} AM1.5G(\lambda)d\lambda = 2.1\cdot10^{21}\,m^{-2}s^{-1}$. (Total number of photons in AM1.5 = $3.89\cdot10^{21}$ photons·m^{-2}·s^{-1}) To calculate the QFLS we assumed Lambertian emission and utilize Würfels generalized Planck law [13], which relates the spontaneous emission of photons in a direct semiconductor to the chemical potential of the non-equilibrium charge carrier concentration to the local temperature T providing that the specific absorptivity α(E) is known.

$$I_{PL}(E) = \frac{2\pi E^2 a(E)}{h^3c^2}\cdot\frac{1}{\exp\left(\frac{E-QFLS}{k_BT}-1\right)} \tag{4}$$

Assuming that the spectral absorptivity approaches unity for photon energies above the bandgap this equation can be simplified to

$$\ln\left(\frac{I_{PL}(E)h^3c^2}{2\pi E^2}\right) = -\frac{E}{k_BT}+\frac{\mathrm{QFLS}}{k_BT} \tag{5}$$

Here I_{PL} is the measured absolute photoluminescence, E the photon energy, k_B the Boltzmann constant, T the temperature, c the speed of light, h the Planck constant. By fitting equation (5) to the high-energy slope of the PL emission, the QFLS and the local charge carrier temperature can be extracted. [14]

For the Suns-QFLS dependence, absolute photoluminescence maps were recorded while varying the excitation intensity between 1 sun and 0.08 sun using a hyperspectral widefield imager from Photon etc. at a magnification of 20x as described above. All maps were averaged to increase the signal-to-noise ratio, and the resulting spectra were fitted to equation (5) to extract the QFLS. In all cases, the local temperature of the probed emissive volume was found to be slightly elevated, and we thus assumed a k_BT of 0.029 eV for the calculation of the internal ideality factor.

Photoluminescence Lifetime Imaging: A confocal single-photon counting fluorescence microscope from Picoquant was used to record photoluminescence lifetime maps of the perovskite top cell. Excitation was performed at 636 nm using a 100x long working distance air objective (NA = 0.8), a repetition rate of 5 MHz, and an intensity of 380 nJ/cm^2/pulse. The photoluminescence was collected through a dichroic mirror, a 640 nm long-pass filter, and a 50 µm pinhole onto a single photon counting SPAD detector. Both the excitation and emission were raster-scanned using a galvo mirror system, where both the objective and sample remain at a fixed position.

(Macro) Photoluminescence Measurements*:* Photoluminescence spectra and transients of the SHJ bottom cell were recorded using a prism monochromator equipped with an InGaAs detector after excitation from a pulsed dye laser with a wavelength of λ_{exc} = 505 nm or 365 nm. The laser fluence amounted to 6 µJ/cm^2 for transient measurements and to 12 mJ/cm^2 in the case of spectral dependent measurements. A continuous-wave temperature controlled 910 nm laser (Thorlabs) was used to photo excite the CIGS bottom cell through the perovskite top cell for spectral dependent measurements. The emission was recorded using an Andor IDus DU490A InGaAs detector. The spot size was recorded using a Thorlabs beam profiler, where the size was set to be to where the intensity of the beam falls to 1/e^2. The laser fluence amounted to 6.1 W/cm^2.

(Macro) Transient Photoluminescence: Transient measurements of the CIGS sub-cell in the perovskite/CIGS tandem were recorded using a confocal single-photon counting microscope from Picoquant equipped with a 10x air objective. Excitation was performed using a pulsed 636 nm laser at a repetition rate of 20 MHz and a fluence of 160 mJ/cm^2. According to the spectral response measurements shown in Figure 2, 8 % of incident 636 nm light is absorbed in the CIGS bottom-cell. The effective excitation fluence of the CIGS absorber thus amounts to 13 mJ/cm^2. Photoluminescence of the CIGS absorber was collected through a dichroic mirror and two consecutive long-pass edge filters (cut-on wavelength of 950 nm and 1100 nm) onto a single photon counting SPAD detector. This allowed an effective suppression of the emission from the perovskite top-cell (maximum at λ = 765 nm).

SRIM Simulations: To estimate the non-elastic damage of monodirectional 68 MeV proton irradiation, a total of $5 \cdot 10^7$ protons were simulated using SRIM [15]. Employed densities were: ρ_{LiF} =2.63 g/cm^3, ρ_{IZO} =6.64 g/cm^3, ρ_{SnO2} =6.85 g/cm^3, ρ_{C60} =1.65 g/cm^3, ρ_{Pero} =4.59 g/cm^3, ρ_{PTAA} =1.2g/cm^3, ρ_{NiOX}=6.67 g/cm^3, ρ_{ZnO}=5.1 g/cm^3 ρ_{CdS}=4.82 g/cm^3, ρ_{CIGS}=5.7 g/cm^3, ρ_{Mo}=10.28 g/cm^3, $\rho_{nc\text{-}SiOX}$=2.3 g/cm^3, $\rho_{a\text{-}Si}$=2.285 g/cm^3, ρ_{Si}=2.285 g/cm^3, ρ_{ZnOAl}=5.1 g/cm^3, ρ_{quartz} =1.72 g/cm^3, ρ_{LiF} =2.63 g/cm^3, ρ_{ITO} =7.2 g/cm^3, and ρ_{Ag} =10.49 g/cm^3. To approximate the damage induced by omnidirectional, poly-energetic irradiation, the proton spectrum at an ISS orbit (see Fig S1) was used. Using Python[16] and PYSRIM[17] automated SRIM simulations were performed varying the incident proton energy as well as the incident angle between 0 and 90° in 10°steps. In case of the perovskite/CIGS tandem solar cell, the depth-averaged energy loss of monoenergetic 68 MeV proton irradiation amounted to 6.3×10^{-5} eV/(Å ion), while for the ISS orbit an average of 4.34×10^{-5} eV/(Å ion) is obtained. With a total flux of about 1×10^3 p^+ cm^{-2} s^{-1} in the ISS orbit, a dose of 2×10^{12} would be accumulated after around 90 years. While this is true for solar cells mounted on the satellite surface, free-standing solar arrays are exposed from both sides, and therefore a dose of 2×10^{12} p^+ cm^{-2} would be reached earlier after about 50 years.

Supplemental References

1. Köhnen, E., Jošt, M., Morales-Vilches, A.B., Tockhorn, P., Al-Ashouri, A., Macco, B., Kegelmann, L., Korte, L., Rech, B., Schlatmann, R., et al. (2019). Highly efficient monolithic perovskite silicon tandem solar cells: analyzing the influence of current mismatch on device performance. Sustain. Energy Fuels *3*, 1995–2005.

2. Lang, F., Nickel, N.H., Bundesmann, J., Seidel, S., Denker, A., Albrecht, S., Brus, V. V., Rappich, J., Rech, B., Landi, G., et al. (2016). Radiation Hardness and Self-Healing of Perovskite Solar Cells. Adv. Mater. *28*, 8726–8731.

3. Jošt, M., Bertram, T., Koushik, D., Marquez, J.A., Verheijen, M.A., Heinemann, M.D., Köhnen, E., Al-Ashouri, A., Braunger, S., Lang, F., et al. (2019). 21.6%-Efficient Monolithic Perovskite/Cu(In,Ga)Se 2 Tandem Solar Cells with Thin Conformal Hole Transport Layers for Integration on Rough Bottom Cell Surfaces. ACS Energy Lett. *4*, 583–590.

4. Repins, I., Contreras, M.A., Egaas, B., DeHart, C., Scharf, J., Perkins, C.L., To, B., and Noufi, R. (2008). 19·9%-efficient ZnO/CdS/CuInGaSe 2 solar cell with 81·2% fill factor. Prog. Photovoltaics Res. Appl. *16*, 235–239.

5. Koushik, D., Jošt, M., Dučinskas, A., Burgess, C., Zardetto, V., Weijtens, C., Verheijen, M.A., Kessels, W.M.M., Albrecht, S., and Creatore, M. (2019). Plasma-assisted atomic layer deposition of nickel oxide as hole transport layer for hybrid perovskite solar cells. J. Mater. Chem. C *7*, 12532–12543.

6. Denker, A., Rethfeldt, C., Röhrich, J., Berlin, H., Cordini, D., Heufelder, J., Stark, R., Weber, A., and Berlin, B.H. (2010). Status of the Hzb # Cyclotron : Eye Tumour Therapy in Berlin Patient Numbers and Special. Proc. CYCLOTRONS 2010, (Lanzhou, China), 75–77.

7. Röhrich, J., Damerow, T., Hahn, W., Müller, U., Reinholz, U., and Denker, A. (2012). A Tandetron^{TM} as proton injector for the eye tumor therapy in Berlin. Rev. Sci. Instrum. *83*, 02B903.

8. Jasenek, A., Boden, A., Weinert, K., Balboul, M.R., Schock, H.W., and Rau, U. (2001). High-Energy Electron and Proton Irradiation of Cu(In,Ga)Se 2 Heterojunction Solar Cells. MRS Proc. *668*, H3.2.

9. Khatri, I., Lin, T.-Y., Nakada, T., and Sugiyama, M. (2019). Proton Irradiation on Cesium-Fluoride-Free and Cesium-Fluoride-Treated Cu(In,Ga)Se 2 Solar Cells and Annealing Effects under Illumination. Phys. status solidi – Rapid Res. Lett., 1900519.

10. Jasenek, A., Schock, H.W., Werner, J.H., and Rau, U. (2001). Defect annealing in Cu(In,Ga)Se2 heterojunction solar cells after high-energy electron irradiation. Appl. Phys. Lett. *79*, 2922–2924.

11. Mintairov, M.A., Kalyuzhnyy, N.A., Evstropov, V. V., Lantratov, V.M., Mintairov, S.A., Shvarts, M.Z., Andreev, V.M., and Luque, A. (2015). The segmental approximation in multijunction solar cells. IEEE J. Photovoltaics *5*, 1229–1236.

12. Delamarre, A., Lombez, L., and Guillemoles, J.F. (2012). Contactless mapping of saturation currents of solar cells by photoluminescence. Appl. Phys. Lett. *100*, 2012–2015.

13. Würfel, P. (1982). The chemical potential of radiation. J. Phys. C Solid State Phys. *15*, 3967–3985.

14. Unold, T., and Gütay, L. (2011). Advanced Characterization Techniques for Thin Film Solar Cells (JohnWiley & Sons, Ltd).

15. Ziegler, J.F., Ziegler, M.D.D., and Biersack, J.P.P. (2010). SRIM - The stopping and range of ions in matter (2010). Nucl. Instruments Methods Phys. Res. Sect. B Beam Interact. with Mater. Atoms *268*, 1818–1823.

16. Jones, E., Oliphant, T., Peterson, P., and Al., E. (2001). SciPy: Open source scientific tools for Python. http://www.scipy.org/.

17. Ostrouchov, C., Zhang, Y., and J. Weber, W. (2018). pysrim: Automation, Analysis, and Plotting of SRIM Calculations. J. Open Source Softw. *3*, 829.

18. SPENVIS -- Space Environment Information System, funded by the European Space Agency (ESA) (2019). http://www.spenvis.ome.be/spenvis.

19. Babaro, J.P., West, K.G., and Hamadani, B.H. (2016). Spectral response measurements of multijunction solar cells with low shunt resistance and breakdown voltages. Energy Sci. Eng. *4*, 372–382.

20. Stolterfoht, M., Wolff, C.M., Márquez, J.A., Zhang, S., Hages, C.J., Rothhardt, D., Albrecht, S., Burn, P.L., Meredith, P., Unold, T., et al. (2018). Visualization and suppression of interfacial recombination for high-efficiency large-area pin perovskite solar cells. Nat. Energy *3*, 847–854.

21. Lang, F., Jošt, M., Bundesmann, J., Denker, A., Albrecht, S., Landi, G., Neitzert, H.-C.C., Rappich, J., and Nickel, N.H. (2019). Efficient minority carrier detrapping mediating the radiation hardness of triple-cation perovskite solar cells under proton irradiation. Energy Environ. Sci. *12*, 1634–1647.

22. Arutyunov, N., Emtsev, V., Krause-Rehberg, R., Elsayed, M., Kessler, C., Kozlovski, V., and Oganesyan, G. (2015). Positron probing of phosphorus-vacancy complexes in silicon irradiated with 15 MeV protons. J. Phys. Conf. Ser. *618*, 8–12.

23. Yamaguchi, M., Khan, A., Taylor, S.J., Ando, K., Yamaguchi, T., Matsuda, S., and Aburaya, T. (1999). Deep level analysis of radiation-induced defects in Si crystals and solar cells. J. Appl. Phys. *86*, 217–223.

24. Karazhanov, S.Z. (2000). Effect of radiation-induced defects on silicon solar cells. J. Appl. Phys. *88*, 3941–3947.

25. Watkins, G.D. (2000). Intrinsic defects in silicon. Mater. Sci. Semicond. Process. *3*, 227–235.

26. Crabb, R.L. (1994). Solar cell radiation damage. Radiat. Phys. Chem. *43*, 93–103.

27. Hu, Z., He, S., and Yang, D. (2004). Effect of <200 keV proton radiation on electric properties of silicon solar cells at 77 K. Nucl. Instruments Methods Phys. Res. Sect. B Beam Interact. with Mater. Atoms *217*, 321–326.

28. Srour, J.R., Marshall, C.J., and Marshall, P.W. (2003). Review of displacement damage effects in silicon devices. IEEE Trans. Nucl. Sci. *50*, 653–670.

29. Neitzert, H.C., Spinillo, P., Bellone, S., Licciardi, G.D., Tucci, M., Roca, F., Gialanella, L., and Romano, M. (2004). Investigation of the damage as induced by 1.7 MeV protons in an amorphous/crystalline silicon heterojunction solar cell. Sol. Energy Mater. Sol. Cells *83*, 435–446.

30. Neitzert, H.-C., Ferrara, M., Kunst, M., Denker, A., Kertész, Z., Limata, B., Gialanella, L., and Romano, M. (2008). Electroluminescence efficiency degradation of crystalline silicon solar cells after irradiation with protons in the energy range between 0.8 MeV and 65 MeV. Phys. status solidi *245*, 1877–1883.

31. Hisamatsu, T., Kawasaki, O., Matsuda, S., Nakao, T., and Wakow, Y. (1998). Radiation degradation of large fluence irradiated space silicon solar cells. Sol. Energy Mater. Sol. Cells *50*, 331–338.

32. Imaizumi, M., Sumita, T., Kawakita, S., Aoyama, K., Anzawa, O., Aburaya, T., Hisamatsu, T., and Matsuda, S. (2005). Results of flight demonstration of terrestrial solar cells in space. Prog. Photovoltaics Res. Appl. *13*, 93–102.

33. Yamaguchi, M., Taylor, S.J., Yang, M.J., Matsuda, S., Kawasaki, O., and Hisamatsu, T. (1996). High-energy and high-fluence proton irradiation effects in silicon solar cells. J. Appl. Phys. *80*,

4916–4920.

34. Sahli, F., Werner, J., Kamino, B.A., Bräuninger, M., Monnard, R., Paviet-Salomon, B., Barraud, L., Ding, L., Diaz Leon, J.J., Sacchetto, D., et al. (2018). Fully textured monolithic perovskite/silicon tandem solar cells with 25.2% power conversion efficiency. Nat. Mater. *17*, 820–826.

35. Bush, K.A., Palmstrom, A.F., Yu, Z.J., Boccard, M., Cheacharoen, R., Mailoa, J.P., McMeekin, D.P., Hoye, R.L.Z., Bailie, C.D., Leijtens, T., et al. (2017). 23.6%-efficient monolithic perovskite/silicon tandem solar cells with improved stability. Nat. Energy *2*, 17009.

36. Jošt, M., Köhnen, E., Morales-Vilches, A.B., Lipovšek, B., Jäger, K., Macco, B., Al-Ashouri, A., Krč, J., Korte, L., Rech, B., et al. (2018). Textured interfaces in monolithic perovskite/silicon tandem solar cells: advanced light management for improved efficiency and energy yield. Energy Environ. Sci. *11*, 3511–3523.

37. Adey, J., Jones, R., Palmer, D.W., Briddon, P.R., and Öberg, S. (2004). Degradation of boron-doped Czochralski-grown silicon solar cells. Phys. Rev. Lett. *93*, 1–4.

38. Schmidt, J., and Bothe, K. (2004). Structure and transformation of the metastable boron- and oxygen-related defect center in crystalline silicon. Phys. Rev. B *69*, 024107.

39. Mailoa, J.P., Bailie, C.D., Johlin, E.C., Hoke, E.T., Akey, A.J., Nguyen, W.H., McGehee, M.D., and Buonassisi, T. (2015). A 2-terminal perovskite/silicon multijunction solar cell enabled by a silicon tunnel junction. Appl. Phys. Lett. *106*, 121105.

40. Lang, F., Shargaieva, O., Brus, V. V., Neitzert, H.C., Rappich, J., and Nickel, N.H. (2018). Influence of Radiation on the Properties and the Stability of Hybrid Perovskites. Adv. Mater. *30*, 1702905.

41. Köppel, G., Eisenhauer, D., Rech, B., and Becker, C. (2017). Combining tailor-made textures for light in-coupling and light trapping in liquid phase crystallized silicon thin-film solar cells. Opt. Express *25*, A467.

42. American Society for Testing and Materials (ASTM) Terrestrial Reference Spectra for Photovoltaic Performance Evaluation (2017). Reference Solar Spectral Irradiance Air Mass 1.5 ASTM G173. http://rredc.nrel.gov/solar/spectra/am1.5/.
